# Sentence comprehension in Lewy body diseases: a functional magnetic resonance imaging study

**DOI:** 10.1093/braincomms/fcaf423

**Published:** 2025-10-30

**Authors:** Lubomira Novakova, Martin Gajdoš, Daniel Carbol, Irena Rektorova

**Affiliations:** Applied Neuroscience Research Group, Central European Institute of Technology – CEITEC, Masaryk University, Brno 62500, Czech Republic; Applied Neuroscience Research Group, Central European Institute of Technology – CEITEC, Masaryk University, Brno 62500, Czech Republic; Applied Neuroscience Research Group, Central European Institute of Technology – CEITEC, Masaryk University, Brno 62500, Czech Republic; Faculty of Medicine, Masaryk University, Brno 62500, Czech Republic; Applied Neuroscience Research Group, Central European Institute of Technology – CEITEC, Masaryk University, Brno 62500, Czech Republic; First Department of Neurology, Faculty of Medicine and St. Anne’s University Hospital, Brno 60200, Czech Republic

**Keywords:** language dysfunctions, mild cognitive impairment, Lewy body diseases, functional MRI, brain connectivity

## Abstract

Lewy body diseases, including Parkinson’s disease and dementia with Lewy bodies, often involve mild cognitive impairment at diagnosis (mild cognitive impairment with Lewy bodies (MCI-LB). Language dysfunction in MCI-LB patients is often unrecognized. This study aimed to assess syntactic comprehension deficits in MCI-LB patients and to explore their neural correlates. A total of 25 MCI-LB patients (mean ± sd: 72 ± 5.6 years old, 10 women) and 25 healthy controls (HC, mean ± sd: 66 ± 4.0 years old, 12 women) performed task functional MRI Test of Sentence Comprehension (ToSC). Functional connectivity was analysed using psychophysiological interaction (PPI) method, focusing on the striatum and language networks. MCI-LB patients had lower ToSC scores than HC (MCI-LB: 74.7 ± 15.7, HC: 88.5 ± 9.0, *P* < 0.001) and their PPI analysis revealed decreased connectivity from the striatum to the cuneus, precuneus, and left supramarginal gyrus, and reduced connectivity particularly in the dorsal pathway during noncanonical (syntactically more complex) sentence processing. Taken together, in this cross-sectional study MCI-LB patients showed impaired sentence comprehension related to decreased subcortical-cortical and dorsal language network connectivity. Specific changes in frontotemporal connectivity in MCI-LB might be a promising indicator of language related cognitive impairment in these a-synucleinopathies.

## Teaser

Novakova *et al*. report that patients with mild cognitive impairment with Lewy bodies demonstrated impaired comprehension of syntactically complex sentences as compared to healthy controls. Task-related reduced functional connectivity in these patients within specific subcortical-cortical and language networks was identified and distinct alterations in the dorsal pathway were linked to processing of syntactically complex sentences.

## Graphical Abstract

**Graphical Abstract:**
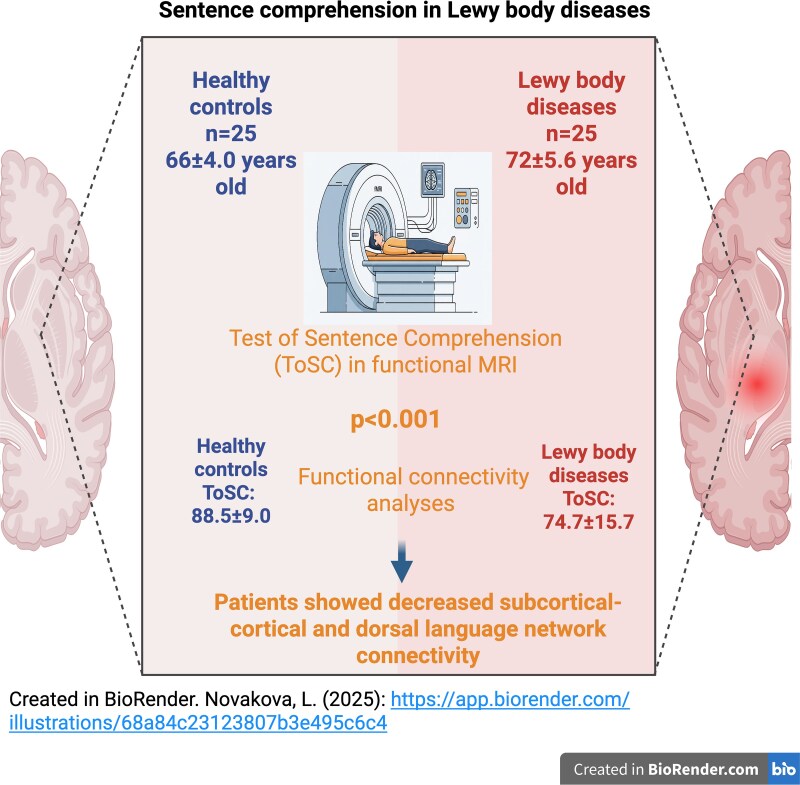

## Introduction

**Figure 1:**
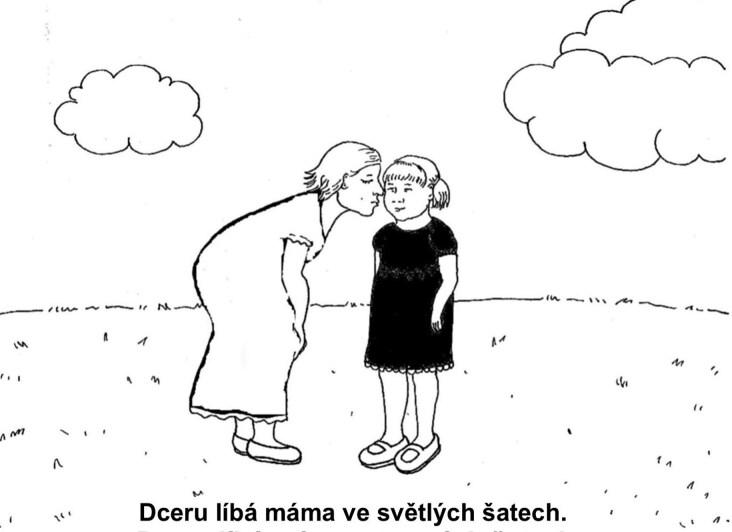
**Test of sentence comprehension, sample picture**. English translation: The daughter (accusative) is kissed by the mother (nominative) in the white dress (noncanonical word order in Czech, active). We have the permissions from the original authors of the images to include it in our manuscript (Nohová et al. 2022).^2^

One of the most important human functions is the ability to communicate; the loss or limitation of this ability has devastating impacts on the individual and those around them. Language processing and the use of complex sentence structures are key features that distinguish human language from other forms of communication observed in non-human species. Syntactic comprehension is a hallmark of human communication, enabling the understanding and interpretation of how words are arranged in a sentence to express specific meanings.^1^ In English, grammatical relations between words are primarily determined by a fixed word order. Changing the order of the nouns in a sentence, such as from *The mother is kissing the daughter* to *The daughter is kissing the mother* alters the meaning entirely. Noncanonical sentences (The daughter is kissed by the mother, non-canonical order in English is accomplished mostly by using passive structures) include syntactically complex sentences in which the first noun is a receiver of action (not a doer), making them more difficult to understand. In contrast, Slavic languages like Czech rely on a more flexible word order, with grammatical relations indicated by case markings on nouns (e.g. nominative–NOM and accusative–ACC). For instance, a Czech sentence in a canonical word order might be *Máma (NOM) líbá dceru (ACC),* meaning *The mother is kissing the daughter.* A noncanonical equivalent, *Dceru (ACC) líbá mámu (NOM),* conveys the same meaning—*The mother is kissing the daughter*—despite the different word order.^3^ For an example of the test task see Fig. 1.

Functional magnetic resonance imaging enables the study of the neural correlates of language function in living humans. The functional connectivity of the language network (domain-specific areas) consists of two basic pathways: dorsal (connecting the inferior frontal gyrus and the premotor area with the posterior superior temporal gyrus and inferior parietal lobe via the arcuate fasciculus), which is mainly involved in auditory-motor integration, speech production and repetition, and phonological processing; and ventral (connecting the superior and middle temporal gyrus, inferior parietal lobe, and occipital lobe with the inferior frontal gyrus via the inferior fronto-occipital fasciculus), which is mainly involved in speech comprehension and semantic processing.^4^ In addition to domain-specific areas, a domain-general network related to language is also involved: the bilateral fronto-insular-parietal system, which reflects general cognitive efforts proportional to the difficulty of the task.^5^

Neuronal Lewy body diseases (LBDs) consist of two major clinical entities – Parkinson’s disease (PD) and dementia with Lewy bodies (LB). The vast majority of patients with LBDs already have mild cognitive impairment (MCI) at the time of the diagnosis. Language dysfunctions in patients with LBDs with MCI (MCI-LB) are often unrecognized and negatively affect the patient’s quality of life. Behavioural and neuroimaging studies previously demonstrated that patients with LBDs exhibit deficits in sentence comprehension, particularly when processing complex syntactic structures.^6-11^ In healthy subjects, a meta-analysis of functional imaging studies has suggested the dorsal language pathway as critical for processing syntactically complex sentences.^12^ Complementary, evidence from patients with post-stroke aphasia and primary progressive aphasia further shows that damage to the dorsal pathway strongly predicts deficits in syntactic comprehension.^13-16^

We previously found that Slovak patients with PD without MCI already have problems with sentence reading comprehension with altered task-dependent functional connectivity.^3^ We aimed to continue our previous research^3^ and describe specific alterations in comprehending syntactically complex sentences in LBDs that already have MCI (MCI-LB patients) as compared to healthy controls (HC) and identify the neural underpinnings of these deficits using functional connectivity analysis from the striatum (the area involved in syntactic sentence processing and affected by the disease) and in language areas (divided into dorsal and ventral pathways^4^). We hypothesized that MCI-LB patients demonstrate greater behavioural impairments in comprehending syntactically complex sentences than HC. These deficits would be especially apparent for noncanonical sentences (our sentences of interest), and we would be able to identify neural correlates of behavioural deficits in MCI-LB as compared to HC in striato-cortical and language-related domain-specific networks. We further hypothesized that MCI-LB patients would show reduced functional connectivity of these large-scale brain networks compared to HC.

## Materials and methods

### Participants

A total of 25 MCI-LB (mean ± sd: 72 ± 5.6 years old) and 25 HC (mean ± sd: 66 ± 4.0 years old) participated in the fMRI study. Inclusion criteria were right-handedness, Czech as their first language, age (60–80 years), presence of PD-MCI^17^ or MCI-LB.^18^ We recruited participants from our study cohorts.^19,20^ All participants underwent a neuropsychological examination prior to fMRI scanning that consisted of a short neuropsychological battery^21^ (testing six cognitive domains: short-term memory, visuospatial functions, language functions, attention, executive functions, and long-term memory), the Montreal Cognitive Assessment test (MoCA),^22^ the Boston Naming Test (BNT),^23^ the Geriatric Depression Scale (GDS)^24^ and the Czech version of the Functional Activities Questionnaire (FAQcz).^(25)^ MCI subjects had subjective cognitive complaints and scored with at least two neuropsychological tests with a cutoff score under −1.5 standard deviation (SD) below the age-appropriate norms or with one test with a cutoff score under −1.5 SD below the age-appropriate norms plus a MoCA cutoff score of 26. PD-MCI patients had MCI and clinically established PD, were longitudinally followed by a neurologist, and were on a stable dopaminergic medication (mean Levodopa Equivalent Dose = 942 ± 399.6) at least 4 weeks prior to the baseline assessment and during the whole study and were tested in the ON medication state without dyskinesias. MCI-LB subjects were diagnosed with MCI and had at least two core clinical features of MCI-LB (fluctuating cognition, visual hallucinations, or REM sleep behaviour disorder, and one or more spontaneous cardinal features of parkinsonism). The exclusion criteria were a cardiac pacemaker or any MRI-incompatible metal in the body, epilepsy, any diagnosed psychiatric disorder, alcohol/drug abuse, and for the HC group additionally the presence of LBDs or other neurogenerative disorder or MCI/dementia. For more details see Table 1. The Mann-Whitney U-test was used to calculate the differences between MCI-LB and HC. All subjects signed an informed consent form that had been approved by the local ethics committee.

**Table 1:** Characteristics of the sample

Factor	MCI-LBD/PD	HC	Test statistics of the between-group difference
*N*	25	25	
Demographic variables			
Sex			*X* ^2^ (1, *N* = 50) = 0.33
Female (*n*)	10	12	
Male (*n*)	15	13	
Age (M ± SD)	72 (± 5.54)	66.2 (± 3.94)	**U** **=** **114.5, Z** **=** **−3.85**^a^
Education Length (M ± SD)	14.84 (± 3.81)	16.2 (± 2.78)	U = 274.5, Z = −0.737
Neuropsychological Tests (M ± SD)			
MoCA score	23.56 (± 2.45)	27.28 (± 1.34)	**U** **=** **121.5, Z** **=** **−3.71**^a^
Short-term memory z-score	−1.04 (± 0.76)	−0.08 (± 0.75)	U = 165, Z = −2.86
Visuo-spatial functions z-score	−0.01 (± 0.5)	0.65 (± 0.53)	**U** **=** **143, Z** **=** **−3.29**^a^
Attention z-score	−0.77 (± 0.68)	−0.32 (± 0.76)	U = 291, Z = −0.42
Executive functions z-score	−1.22 (± 0.65)	−0.12 (± 0.63)	**U** **=** **134, Z** **=** **−3.46**^a^
Long-term memory z-score	−1.39 (± 0.64)	−0.49 (± 0.59)	U = 163, Z = −2.9
Language functions z-score	−0.67 (± 0.55)	1.12 (± 0.4)	**U** **=** **145, Z** **=** **−3.25**^a^
Boston naming test score	25.76 (± 2.95)	28.56 (± 1.58)	U = 207, Z = −2.05
GDS score	3.12 (± 3.13)	0.84 (± 1.14)	U = 226.5, Z = −1.67
FAQcz score	1.92 (± 2.97)	0 (± 0)	U = 189.5, Z = −2.39

*Note:* Education Length is quantified as the total number of years of formal education completed. Due to the distribution of most variables not meeting the assumption of normality, non-parametric tests were performed. MoCA, Montreal Cognitive Assessment; GDS, Geriatric Depression Scale; FAQcz, Functional Activities Questionnaire, Czech version; *M*, mean; SD, standard deviation.

^a^Values in bold indicate that the difference is significant at the *P* < 0.05 level after adjusting the variables for the effect of age and applying the Bonferroni correction.

### MRI examination

The 3T Siemens Prisma MR scanner (Siemens Corp., Erlangen, Germany) was used for data acquisition at the Central European Institute of Technology (CEITEC), Masaryk University in Brno, using the T1 MPRAGE sequence (TR 2.400 ms; TE 2.27 ms; voxel size 0.85 × 0.85 × 0.85 mm; FoV 218 × 218 mm; flip angle 8°; 192 sagittal slices) and multiecho BOLD fMRI sequence for two sessions of the task fMRI [TR 980 ms; TE (14.0, 34.6, 55.3) ms; voxel size 2.5 × 2.5 × 2.5 mm; FoV 200 mm; flip angle 50°; 60 axial slices; 500 scans per session; multiband factor 5].

### fMRI task

We analysed the performance of the Czech version of the ToSC^2^ adjusted for fMRI. This was a slightly modified version of the fMRI task used in our previous research, in which the sentences were in Slovak.^3^ Subjects viewed pictures in conjunction with the sentences from the ToSC on the screen during fMRI and decided whether the sentences matched the pictures by pressing a YES or NO button. The fMRI task consisted of 96 trials, divided into 2 sessions (48 trials each); the duration of the whole fMRI task was 16 min. Canonical and noncanonical word order sentences were used (ratio of use: 1:1). The rate of correct and false statements was 1:1 (48 to 48). All participants were properly instructed and practiced the task before they were scanned. The percentage of correct answers, reflecting accuracy, was our outcome measure.

### Data analysis

The fMRI data was preprocessed using the SPM 12 toolbox running under Matlab 2017b (MathWorks, Inc.); this included realignment, multiecho merging based on CNR,^26^ spatial normalization, and smoothing (5 mm FWHM Gaussian filter). Levels of excessive motion set to the framewise displacement metric exceeded 0.5 mm in more than 20% of the scans in each of the sessions. Two MCI-LB subjects were excluded from analysis due to excessive movements. We then controlled the data for spatial abnormalities (e.g. dropouts) with the Mask Explorer tool.^27^

**Figure 2:**
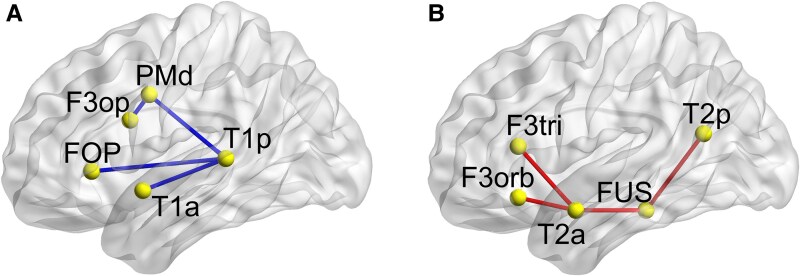
**Dorsal and ventral language pathways.** Dorsal (**A**) and ventral (**B**) pathways for language with region of interests (ROIs) that were used for the psychophysiological interaction (PPI) analysis; T1a/p: anterior/posterior superior temporal gyrus; T2a/p: anterior/posterior middle temporal gyrus; FUS: fusiform gyrus; F3orb/tri/op: pars orbitalis/triangularis and opercularis of the inferior frontal gyrus; FOP: deep frontal operculum; PMd, dorsal premotor cortex. Figure was created using BrainNet Viewer, Xia *et al*. (2013), MNI coordinates for ROIs were adapted from Sauer *et al*. 2008.

We used a mask of the whole striatum based on the structural anatomical striatal atlas^28^ available within the FSL software package. We used five peak coordinates for the ventral and the dorsal language pathways, according to previously published work,^4^ see Fig. 2. Spheres with the centroid at the corresponding peak voxel (radius = 6 mm) were created and intersected with the fMRI group mask. The final masks then served as ventral and dorsal language network region of interests (ROIs) and were used in the subsequent analyses.

Two task-dependent functional connectivity analyses (from the whole striatum to the rest of brain and in between the ROIs of language areas^4^) were studied using the psychophysiological interaction method (PPI)^29^ with age (which differed between both groups) used as a nuisance regressor. We used a general linear model with regressors for PPI interactions for canonical and noncanonical conditions, effects of seed, effects of task conditions (canonical, noncanonical), and nuisance regressors (24 regressors for movement, nuisance regressors for white matter signal and for signal from cerebrospinal fluid). T-tests were used to calculate the differences between MCI-LB and HC. For PPI analyses from the striatum to the whole brain, we reported results with a statistical significance threshold set to *P* < 0.05 with FWE correction at the cluster level with an initial cutoff of *P* = 0.001. For PPI analyses between ROIs separated into dorsal and ventral language networks, we reported results with statistical significance threshold set to *P* < 0.05 with FDR correction. ROIs were selected as spheres with a radius of 6 mm with the first eigenvariate as a representative signal.

## Results

### Behavioural results

On the behavioural level, the MCI-LB patients had significantly lower total ToSC scores (mean ± sd: 74.7 ± 15.7) in fMRI than HC (mean ± sd: 88.5 ± 9.0), *P* < 0.001, and the difference was significant for both canonical (MCI-LB-mean ± sd: 82.2 ± 15.6, HC-mean ± sd: 94.0 ± 7.6, *P* = 0.001) and noncanonical sentences (MCI-LB-mean ± sd: 67.5 ± 18.0, HC-mean ± sd: 82.9 ± 12.7, *P* < 0.001).

### Connectivity results

**Figure 3:**
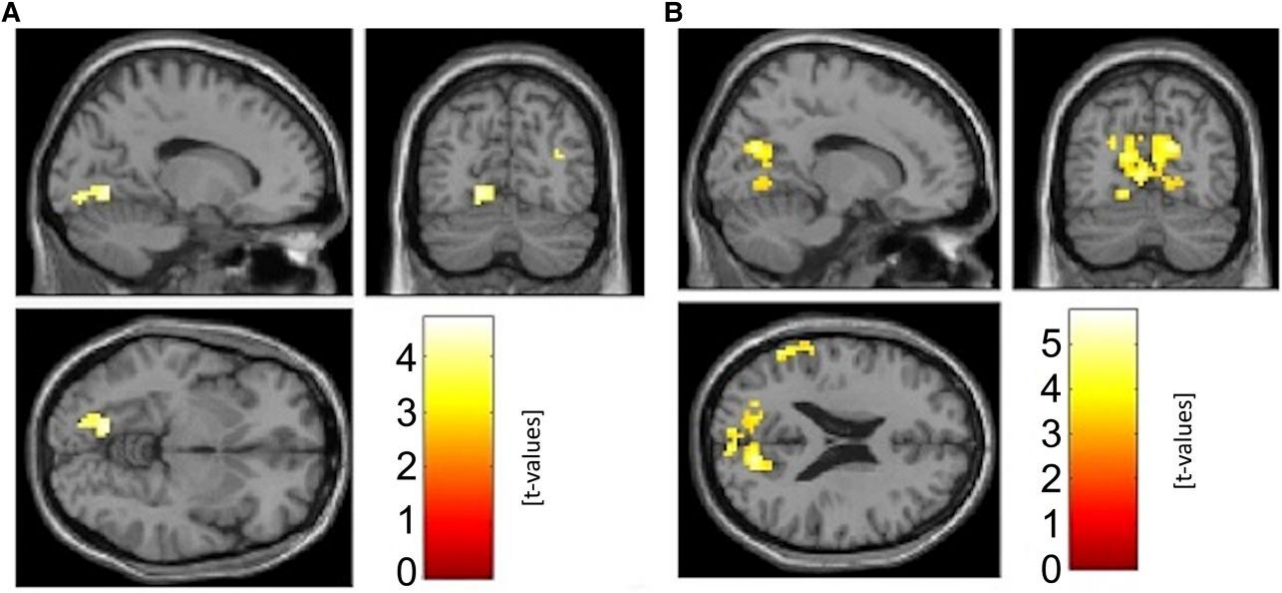
**Connectivity differences, seed striatum, comparing patients versus healthy controls.** Results of the psychophysiological interaction method (PPI) analysis using t-tests comparing patients with mild cognitive impairment with Lewy bodies (MCI-LB, *N* = 25) versus healthy controls (HC, *N* = 25), seed striatum, A: canonical B: noncanonical. In both cases were used two sample t-tests with t-values thresholded with *P* < 0.001.

**Figure 4:**
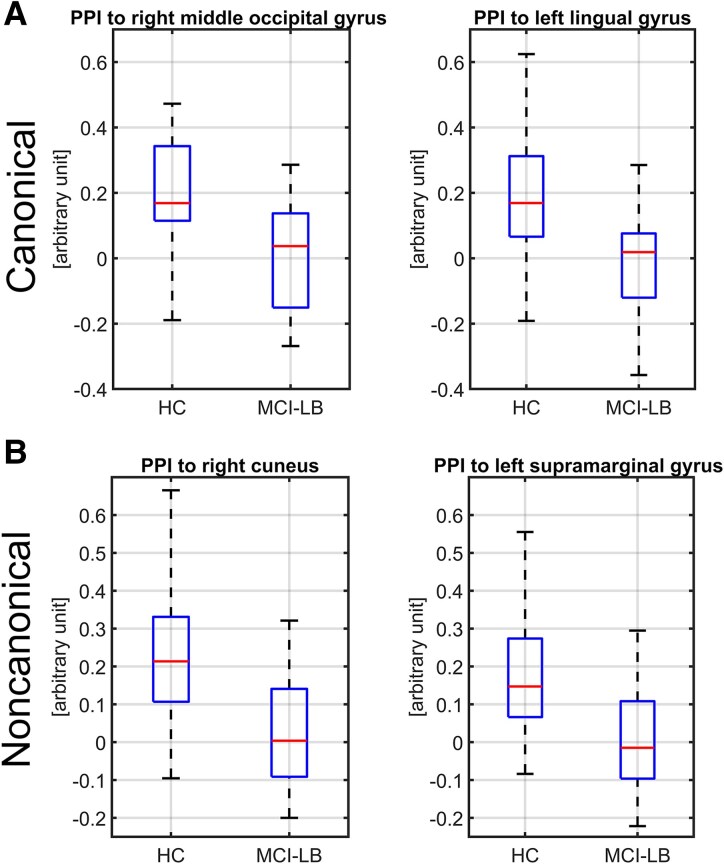
**Boxplots of the psychophysiological interaction (PPI) analysis, seed striatum.** (A) canonical—here difference between groups in connectivity to right middle occipital gyrus has on cluster level inference pFWE = 0.031, and to the left lingual gyrus pFWE = 0.019, (B) noncanonical—here difference in connectivity between groups connectivity to right cuneus has on cluster level inference pFWE < 0.001 and to the left supramarginal gyrus pFWE = 0.007. Difference is computed with two sample *t*-test on patients with mild cognitive impairment with Lewy bodies (MCI-LB, *N* = 25) versus healthy controls (HC, *N* = 25), significant clusters.

Using PPI from the striatum, we found that there was a statistically significant decrease in connectivity to the cuneus/precuneus/lingual gyrus for both canonical and noncanonical sentences in MCI-LB as compared to HC (the decrease in noncanonical sentences was more prominent) and to the left supramarginal gyrus for noncanonical sentences only, see Fig. 3 and Fig. 4. Second PPI of the language networks showed decreased connectivity in MCI-LB subjects as compared to HC during noncanonical condition between ROIs in the dorsal pathway, see Table 2. For all PPI scores, see Supplementary material.

**Table 2:** PPI significant differences in connectivity in non-canonical condition, the dorsal pathway, statistical significance thresholds of *t*-tests were set to *P* < 0.05, FDR corrected

Sign. diff. in	Connection	HC mean ± std	MCI-LB mean ± std	original *P* value *t*-test
noncanonical condition	T1p - T1a	0.223 ± 0.179	0.077 ± 0.243	0.021
	T1a - PMd	0.175 ± 0.182	0.031 ± 0.169	0.007
	T1a - F3op	0.165 ± 0.264	−0.035 ± 0.179	0.004
	FOP - F3op	0.198 ± 0.202	0.016 ± 0.198	0.003
	PMd - F3op	0.264 ± 0.303	0.049 ± 0.275	0.013

T1a/p, anterior/posterior superior temporal gyrus; F3op, pars opercularis of the inferior frontal gyrus; FOP, deep frontal operculum; PMd, dorsal premotor cortex.

## Discussion

This study aimed to assess syntactic comprehension deficits in MCI-LB patients and to explore the neural correlates using functional connectivity analysis of the striatum and language networks. Our behavioural results showed disturbed sentence comprehension in MCI-LB patients with altered task-dependent functional connectivity from the striatum to the cuneus/precuneus/lingual gyrus and to the left supramarginal gyrus. We also found decreased connectivity in dorsal language network in MCI-LB patients as compared to HC. Changes in noncanonical sentences were related specifically to the dorsal pathway disturbances.

The cuneus/precuneus/lingual gyrus are posterior cortical regions that seem to be heavily affected in DLB patients, with these regions showing cortical thinning compared to both HC and patients with Alzheimer’s disease.^30^ Posterior cortical hypometabolism involving the above-mentioned regions and in the supramarginal gyrus was also present in fully blown DLB patients on FDG PET.^31,32^ These areas showed hypometabolism even in the prodromal stages and this was associated with a higher phenoconversion rate to PD/DLB.^33^

A meta-analysis of resting-state functional connectivity in patients with a-synucleinopathy (PD, DLB, multiple system atrophy) showed hypoconnectivity between subcortical regions and the posterior default mode network (precuneus) as compared to HC.^34^ This is in line with our previous study, where we found decreased resting-state functional connectivity between middle striatum and precuneus in PD, which was negatively correlated with executive functions.^35^ Functionally posterior cortical regions are connected with visuospatial processing, executive functions, memory, word processing, and phonological processing.^36-39^ Specifically, the supramarginal gyrus plays an important role in visual word recognition and in phonological decoding, as was seen using transcranial magnetic stimulation.^38,39^

In our study, reduced connectivity between the striatum and dorsal language pathway regions in MCI-LB were not observed in the whole-brain connectivity analyses, apart from the supramarginal gyrus, which can indeed be considered a dorsal pathway component. One possible explanation is that the strong involvement of occipital and ventral visual association cortices in our visually presented sentence comprehension task may have dominated the connectivity patterns. Prior studies have shown that sentence comprehension tasks involving picture stimuli engage rapidly and extensively visual networks in addition to classical language regions.^40,41^ This may have made it more difficult to detect subtle decreases between the striatum and dorsal language areas.

Regarding the results of a PPI analysis reviewing task-related changes of specific seeds of the dorsal and ventral language pathways, we found decreased connectivity in our patient group as compared to HC, such that syntactically more complex sentences were connected to disruptions of dorsal pathway connections. This is in line with previous literature linking syntax specifically to this dorsal language pathway. The processing of complex syntax relies on the posterior superior temporal gyrus/sulcus and BA 44/45 via the dorsal pathway, but its engagement may partly reflect increased working memory demands from greater syntactic complexity.^4^ Other authors^42^ similarly associated higher working memory loads with the dorsal stream. Interestingly, there is also ontological differentiation: while the ventral pathway is present at birth, the dorsal pathway, critical for processing complex syntax, undergoes prolonged maturation and remains incomplete at age seven.^43^ In an awake language mapping study, direct subcortical stimulation showed that the dorsal pathways are critical for organizing words in a sequence necessary for sentence generation.^44^ In fact, these cortico-striatal language pathways are related to complex syntax^45^ and can dynamically adjust for syntactic complexity (canonical versus noncanonical sentences).^46^

Specifically, we have seen that connectivity between anterior part of the superior temporal gyrus and the language areas in the frontal lobule (F3op: pars opercularis of the inferior frontal gyrus; PMd: dorsal premotor cortex) was lower in MCI-LB patients than in HC during noncanonical condition. Specific changes in frontotemporal connectivity were described previously in LBDs and might be a promising indicator of cognitive impairment in these a-synucleinopathies.^47-49^

In our previous study,^3^ we found that patients with PD without MCI performed worse in sentence comprehension than HC and exhibited increased PPI functional connectivity between the right striatum and the supplementary motor area (SMA), which was related to reduced accuracy in noncanonical sentence comprehension. In comparison to our previous study,^3^ the current study with cognitively impaired patients reflects the disease stage when connectivity within specific language networks is decreased. Similar changes, from connectivity increases to decreased connectivity, were previously described in both AD^50^ and PD.^51^

The study limitations include a relatively small sample size, the lack of indicative biomarkers, the possible role of medications used by participants, and the age difference between the patient group and HC, although age was used as a covariate of no interest in our further data analyses.

In summary, MCI-LB patients demonstrated impaired comprehension of syntactically complex sentences as compared to HC. We identified task-related reduced functional connectivity in these patients within specific subcortical-cortical and language networks compared to HC. We identified distinct alterations in the dorsal pathway that were linked to processing of syntactically complex sentences in MCI-LB.

## Supplementary Material

fcaf423_Supplementary_Data

## Data Availability

Anonymized data will be shared with qualified researchers upon reasonable request to the corresponding author. Code used for data analysis is available in the supplementary materials.
