# Supplementary material for: Sentence comprehension in Lewy body diseases: a functional magnetic resonance imaging study
Source: Brain Commun. 2025 Oct 30;7(6):fcaf423. doi: 10.1093/braincomms/fcaf423 (PMC12602868; doi:10.1093/braincomms/fcaf423)
Supplement: fcaf423_Supplementary_Data [file fcaf423_supplementary_data.docx]

# Supplementary materials

Supplementary Table 1: Statistical comparison of PPI connectivity in canonical and noncanonical condition, dorsal pathway is in blue, ventral in red, p-value is for two sample t-test.

| Sign. diff. in | Connection | HC mean ± std | MCI-LB mean ± std | t-test p value |
| --- | --- | --- | --- | --- |
| canonical  condition | T1p - T1a | 0.207 ± 0.200 | 0.130 ± 0.220 | 0.212 |
|  | T1p - FOP | 0.173 ± 0.253 | 0.232 ± 0.277 | 0.444 |
|  | T1p - PMd | 0.135 ± 0.197 | 0.161 ± 0.228 | 0.675 |
|  | T1p - F3op | 0.239 ± 0.283 | 0.145 ± 0.241 | 0.221 |
|  | T1a - FOP | 0.239 ± 0.184 | 0.171 ± 0.269 | 0.308 |
|  | T1a - PMd | 0.171 ± 0.187 | 0.067 ± 0.181 | 0.057 |
|  | T1a - F3op | 0.254 ± 0.214 | 0.076 ± 0.209 | 0.006 |
|  | FOP - PMd | 0.126 ± 0.176 | 0.038 ± 0.185 | 0.097 |
|  | FOP - F3op | 0.225 ± 0.197 | 0.152 ± 0.183 | 0.190 |
|  | PMd - F3op | 0.300 ± 0.291 | 0.130 ± 0.198 | 0.023 |
|  | T2p - T2a | 0.082 ± 0.143 | 0.053 ± 0.153 | 0.487 |
|  | T2p - FUS | 0.057 ± 0.234 | 0.039 ± 0.200 | 0.774 |
|  | T2p - F3tri | 0.168 ± 0.203 | 0.197 ± 0.225 | 0.639 |
|  | T2p - F3orb | 0.254 ± 0.251 | 0.244 ± 0.190 | 0.869 |
|  | T2a - FUS | 0.194 ± 0.244 | 0.117 ± 0.224 | 0.263 |
|  | T2a - F3tri | 0.224 ± 0.212 | 0.088 ± 0.242 | 0.043 |
|  | T2a - F3orb | 0.284 ± 0.273 | 0.166 ± 0.290 | 0.152 |
|  | FUS - F3tri | 0.210 ± 0.213 | 0.138 ± 0.170 | 0.200 |
|  | FUS - F3orb | 0.268 ± 0.306 | 0.200 ± 0.261 | 0.414 |
|  | F3tri - F3orb | 0.285 ± 0.194 | 0.175 ± 0.216 | 0.070 |
| noncanonical condition | T1p - T1a | 0.223 ± 0.179 | 0.077 ± 0.243 | 0.021 |
|  | T1p - FOP | 0.188 ± 0.220 | 0.183 ± 0.199 | 0.938 |
|  | T1p - PMd | 0.128 ± 0.166 | 0.136 ± 0.158 | 0.864 |
|  | T1p - F3op | 0.198 ± 0.241 | 0.112 ± 0.197 | 0.187 |
|  | T1a - FOP | 0.163 ± 0.182 | 0.071 ± 0.190 | 0.094 |
|  | T1a - PMd | 0.175 ± 0.182 | 0.031 ± 0.169 | 0.007 |
|  | T1a - F3op | 0.165 ± 0.264 | -0.035 ± 0.179 | 0.004 |
|  | FOP - PMd | 0.075 ± 0.174 | -0.013 ± 0.135 | 0.059 |
|  | FOP - F3op | 0.198 ± 0.202 | 0.016 ± 0.198 | 0.003 |
|  | PMd - F3op | 0.264 ± 0.303 | 0.049 ± 0.275 | 0.013 |
|  | T2p - T2a | 0.052 ± 0.183 | -0.002 ± 0.130 | 0.243 |
|  | T2p - FUS | 0.068 ± 0.220 | -0.003 ± 0.159 | 0.212 |
|  | T2p - F3tri | 0.096 ± 0.216 | 0.105 ± 0.247 | 0.894 |
|  | T2p - F3orb | 0.205 ± 0.226 | 0.160 ± 0.172 | 0.439 |
|  | T2a - FUS | 0.146 ± 0.242 | 0.107 ± 0.233 | 0.568 |
|  | T2a - F3tri | 0.097 ± 0.221 | 0.024 ± 0.285 | 0.328 |
|  | T2a - F3orb | 0.203 ± 0.286 | 0.026 ± 0.220 | 0.021 |
|  | FUS - F3tri | 0.102 ± 0.229 | 0.029 ± 0.203 | 0.253 |
|  | FUS - F3orb | 0.273 ± 0.264 | 0.149 ± 0.253 | 0.106 |
|  | F3tri - F3orb | 0.205 ± 0.282 | 0.174 ± 0.221 | 0.680 |

Supplementary Table 2: Statistical table with cluster-level inference for parametric map of PPI connectivity in canonical condition with seed in striatum for two sample t-test MCI-LB vs HC. Initial cut-off is p<0.001. Shown are up to 3 local maxima per cluster more than 8.0 mm apart. Labels of coordinates are assigned according to AAL atlas^1^.

| PPI canonical MCI-LB vs HC two sample t-test | | | |
| --- | --- | --- | --- |
| p_FWE_ corrected | cluster size [N vx] | MNI coordinate [mm] | AAL label |
| 0.413 | 18 | [39;-40;-17] | Fusiform R |
| 0.019 | 52 | [-15;-70;-5] | Lingual L |
|  |  | [-15;-85;-8] | Lingual L |
| 0.161 | 28 | [42;-70;-11] | Occipital Inf R |
| 0.539 | 15 | [-33;-76;-41] | Cerebelum Crus2 L |
|  |  | [-30;-67;-41] | Cerebelum Crus2 L |
| 0.831 | 9 | [-21;-1;4] | Putamen L |
| 0.377 | 19 | [-21;-76;25] | Occipital Sup L |
| 0.913 | 7 | [-12;-28;4] | Thalamus L |
| 0.194 | 26 | [12;-73;-8] | Lingual R |
|  |  | [21;-76;-14] | Lingual R |
| 0.031 | 46 | [33;-76;19] | Occipital Mid R |
|  |  | [18;-91;25] | Occipital Sup R |
| 0.913 | 7 | [45;-28;1] | Temporal Sup R |
| 0.968 | 5 | [36;-58;19] | Out of atlas |
| 0.913 | 7 | [-36;-67;22] | Occipital Mid L |
| 0.494 | 16 | [-30;-67;-14] | Fusiform L |
|  |  | [-36;-76;-11] | Occipital Inf L |

Supplementary Table 3: Statistical table with cluster-level inference for parametric map of PPI connectivity in canonical condition with seed in striatum for one sample t-test for MCI-LB + HC. Initial cut-off is p<0.001. Shown are up to 3 local maxima per cluster more than 8.0 mm apart. Labels of coordinates are assigned according to AAL atlas^1^.

| Effect of PPI canonical for HC+MCI-LB - one sample t-test | | | |
| --- | --- | --- | --- |
| p_FWE_ corrected | cluster size [N vx] | MNI coordinate [mm] | AAL label |
| <0.001 | 14695 | [3;-7;7] | Out of atlas |
|  |  | [15;2;13] | Caudate R |
|  |  | [-24;26;-14] | Frontal Inf Orb L |
| <0.001 | 121 | [-9;29;40] | Frontal Sup Medial L |
|  |  | [-6;14;40] | Cingulum Mid L |
|  |  | [-9;11;52] | Supp Motor Area L |
| <0.001 | 101 | [15;2;61] | Supp Motor Area R |
|  |  | [15;-10;55] | Supp Motor Area R |
|  |  | [9;-16;49] | Cingulum Mid R |
| 0.913 | 7 | [-18;-7;-11] | Out of atlas |
| 0.968 | 5 | [3;11;70] | Out of atlas |
| 0.875 | 8 | [-36;8;-38] | Temporal Inf L |
| 0.287 | 22 | [-9;32;22] | Cingulum Ant L |
| 0.913 | 7 | [36;-16;64] | Precentral R |
| 0.785 | 10 | [-39;-31;34] | Out of atlas |
| 0.875 | 8 | [12;47;-2] | Frontal Med Orb R |
| 0.785 | 10 | [0;-52;-20] | Vermis 4 5 |
|  |  | [9;-55;-17] | Cerebelum 4 5 R |
| 0.686 | 12 | [-30;-28;-20] | ParaHippocampal L |
|  |  | [-24;-31;-26] | Cerebelum 4 5 L |
| 0.968 | 5 | [21;-7;-29] | ParaHippocampal R |
| 0.785 | 10 | [-48;-16;19] | Rolandic Oper L |
|  |  | [-54;-16;13] | Postcentral L |
| 0.287 | 22 | [-3;-37;73] | Paracentral Lobule L |
|  |  | [0;-13;76] | Out of atlas |
|  |  | [-6;-52;73] | Precuneus L |
| 0.875 | 8 | [60;2;-5] | Temporal Sup R |
| 0.913 | 7 | [24;-52;43] | Out of atlas |
| 0.832 | 9 | [-21;-10;58] | Frontal Sup L |
| 0.913 | 7 | [-9;47;7] | Cingulum Ant L |
|  |  | [-12;56;1] | Frontal Sup Medial L |
| 0.968 | 5 | [-27;41;16] | Frontal Mid L |
| 0.112 | 32 | [0;-4;-14] | Out of atlas |
|  |  | [6;-7;-20] | Out of atlas |
|  |  | [-6;-4;-20] | Out of atlas |

Supplementary Table 4: Statistical table with cluster-level inference for parametric map of PPI connectivity in canonical condition with seed in striatum for one sample t-test for MCI-LB. Initial cut-off is p<0.001. Shown are up to 3 local maxima per cluster more than 8.0 mm apart. Labels of coordinates are assigned according to AAL atlas^1^.

| Effect of PPI canonical for MCI-LB - one sample t-test | | | |
| --- | --- | --- | --- |
| p_FWE_ corrected | cluster size [N vx] | MNI coordinate [mm] | AAL label |
| <0.001 | 233 | [18;2;10] | Out of atlas |
|  |  | [3;-7;7] | Out of atlas |
|  |  | [12;14;-5] | Caudate R |
| 0.001 | 81 | [-15;-46;-47] | Cerebelum 9 L |
|  |  | [-15;-55;-47] | Cerebelum 9 L |
|  |  | [-21;-64;-50] | Cerebelum 8 L |
| 0.002 | 76 | [-27;50;-5] | Frontal Sup Orb L |
|  |  | [-42;41;-2] | Frontal Inf Tri L |
|  |  | [-24;38;-8] | Out of atlas |
| 0.145 | 27 | [45;-52;-14] | Temporal Inf R |
| 0.247 | 22 | [12;50;-2] | Frontal Med Orb R |
| 0.021 | 46 | [-21;14;7] | Putamen L |
|  |  | [-21;14;-5] | Putamen L |
| 0.047 | 38 | [45;35;-2] | Frontal Inf Tri R |
| 0.002 | 74 | [30;23;28] | Out of atlas |
|  |  | [36;8;25] | Out of atlas |
|  |  | [39;23;31] | Frontal Mid R |
| 0.013 | 51 | [-24;26;-14] | Frontal Inf Orb L |
|  |  | [-30;29;-2] | Frontal Inf Tri L |
|  |  | [-36;26;-11] | Frontal Inf Orb L |
| 0.665 | 12 | [-12;-85;-32] | Cerebelum Crus2 L |
| 0.222 | 23 | [-27;-79;7] | Out of atlas |
| 0.016 | 49 | [33;50;1] | Frontal Mid R |
| 0.095 | 31 | [-18;-22;-5] | Out of atlas |
| 0.720 | 11 | [30;-31;25] | Out of atlas |
| 0.774 | 10 | [-12;-1;10] | Out of atlas |
| 0.827 | 9 | [27;-58;-50] | Cerebelum 8 R |
| 0.180 | 25 | [12;-55;-47] | Cerebelum 9 R |
| 0.222 | 23 | [-30;8;31] | Frontal Mid L |
|  |  | [-36;20;31] | Frontal Mid L |
| 0.145 | 27 | [39;-46;22] | Out of atlas |
|  |  | [45;-37;22] | SupraMarginal R |
| 0.162 | 26 | [-15;-19;10] | Thalamus L |
|  |  | [-24;-22;13] | Out of atlas |
| 0.720 | 11 | [12;-1;61] | Supp Motor Area R |
| 0.106 | 30 | [39;-16;-11] | Out of atlas |
|  |  | [33;-25;-11] | Hippocampus R |
|  |  | [30;-16;-5] | Out of atlas |
| 0.610 | 13 | [21;-40;-29] | Cerebelum 4 5 R |
| 0.145 | 27 | [-36;-58;34] | Angular L |
|  |  | [-30;-52;34] | Angular L |
| 0.558 | 14 | [-3;-52;19] | Precuneus L |
|  |  | [6;-49;16] | Precuneus R |
| 0.720 | 11 | [-18;-10;58] | Out of atlas |
| 0.665 | 12 | [-57;-19;-20] | Temporal Mid L |
|  |  | [-54;-28;-20] | Temporal Inf L |
| 0.949 | 6 | [-30;5;52] | Frontal Mid L |
| 0.417 | 17 | [6;-31;-41] | Out of atlas |
|  |  | [9;-19;-29] | Out of atlas |
| 0.610 | 13 | [-9;-40;31] | Cingulum Mid L |
| 0.417 | 17 | [30;-52;-26] | Cerebelum 6 R |
| 0.007 | 59 | [6;-61;61] | Precuneus R |
|  |  | [-9;-55;55] | Precuneus L |
|  |  | [3;-67;52] | Precuneus R |
| 0.916 | 7 | [0;-22;31] | Cingulum Mid L |
| 0.720 | 11 | [57;-55;19] | Temporal Mid R |
| 0.827 | 9 | [-45;8;13] | Frontal Inf Oper L |
| 0.417 | 17 | [9;32;31] | Cingulum Mid R |
|  |  | [18;32;31] | Out of atlas |
| 0.949 | 6 | [66;-22;-8] | Temporal Mid R |
| 0.827 | 9 | [-27;14;22] | Out of atlas |
| 0.827 | 9 | [18;-4;52] | Out of atlas |
|  |  | [18;-10;58] | Out of atlas |
| 0.916 | 7 | [-30;29;25] | Frontal Mid L |
| 0.973 | 5 | [-6;-22;-23] | Out of atlas |
| 0.774 | 10 | [-6;11;28] | Cingulum Ant L |
| 0.949 | 6 | [18;-76;19] | Cuneus R |
| 0.874 | 8 | [54;-13;22] | Out of atlas |
|  |  | [45;-16;22] | Rolandic Oper R |
| 0.827 | 9 | [-18;-61;28] | Cuneus L |
|  |  | [-12;-55;22] | Precuneus L |
| 0.774 | 10 | [18;-58;25] | Precuneus R |
|  |  | [9;-55;19] | Precuneus R |
| 0.827 | 9 | [36;-61;25] | Out of atlas |
| 0.949 | 6 | [42;-67;43] | Angular R |
| 0.973 | 5 | [9;8;64] | Supp Motor Area R |
| 0.973 | 5 | [48;-67;-11] | Temporal Inf R |
|  |  | [39;-64;-14] | Fusiform R |
| 0.417 | 17 | [-3;14;7] | Caudate L |
|  |  | [-12;20;10] | Caudate L |
| 0.000 | 486 | [-3;-7;19] | Out of atlas |
|  |  | [9;-16;19] | Out of atlas |
|  |  | [24;-37;10] | Out of atlas |
| 0.460 | 16 | [9;20;10] | Out of atlas |
|  |  | [15;26;4] | Out of atlas |
| 0.131 | 28 | [-30;-52;1] | Out of atlas |
|  |  | [-27;-46;13] | Out of atlas |
|  |  | [-27;-40;1] | Hippocampus L |
| 0.827 | 9 | [6;-61;-2] | Lingual R |
| 0.949 | 6 | [3;-70;-11] | Vermis 6 |

Supplementary Table 5: Statistical table with cluster-level inference for parametric map of PPI connectivity in canonical condition with seed in striatum for one sample t-test for HC. Initial cut-off is p<0.001. Shown are up to 3 local maxima per cluster more than 8.0 mm apart. Labels of coordinates are assigned according to AAL atlas^1^.

| Effect of PPI canonical for HC - one sample t-test | | | |
| --- | --- | --- | --- |
| p_FWE_ corrected | cluster size [N vx] | MNI coordinate [mm] | AAL label |
| <0.001 | 10197 | [36;-82;-8] | Occipital Inf R |
|  |  | [3;-7;7] | Out of atlas |
|  |  | [-6;-10;7] | Thalamus L |
| <0.001 | 91 | [42;14;34] | Frontal Inf Oper R |
|  |  | [33;8;43] | Out of atlas |
|  |  | [36;2;52] | Frontal Mid R |
| 0.574 | 13 | [48;-4;-20] | Temporal Mid R |
| 0.002 | 72 | [-36;-1;52] | Precentral L |
|  |  | [-33;17;37] | Frontal Mid L |
|  |  | [-36;-10;49] | Precentral L |
| 0.748 | 10 | [30;-16;-2] | Out of atlas |
| 0.003 | 66 | [3;5;37] | Cingulum Mid R |
|  |  | [9;14;37] | Cingulum Mid R |
|  |  | [9;29;37] | Frontal Sup Medial R |
| <0.001 | 87 | [45;20;22] | Frontal Inf Tri R |
|  |  | [42;29;7] | Frontal Inf Tri R |
|  |  | [51;23;1] | Frontal Inf Tri R |
| 0.574 | 13 | [39;8;16] | Out of atlas |
| 0.470 | 15 | [-12;-31;-29] | Out of atlas |
| 0.903 | 7 | [9;-16;49] | Cingulum Mid R |
| 0.138 | 26 | [-9;26;40] | Frontal Sup Medial L |
|  |  | [-6;17;40] | Frontal Sup Medial L |
| 0.857 | 8 | [-54;-10;-23] | Temporal Mid L |
| 0.857 | 8 | [15;38;22] | Cingulum Ant R |
| 0.574 | 13 | [-9;2;46] | Supp Motor Area L |
| 0.969 | 5 | [15;2;61] | Supp Motor Area R |
| 0.804 | 9 | [9;14;52] | Supp Motor Area R |
| 0.903 | 7 | [18;-58;-26] | Cerebelum 6 R |
| 0.857 | 8 | [30;23;7] | Insula R |
| 0.857 | 8 | [-33;-46;-47] | Cerebelum 8 L |
| 0.903 | 7 | [-18;-37;49] | Out of atlas |
| 0.969 | 5 | [-9;41;-11] | Frontal Med Orb L |
| 0.969 | 5 | [3;-64;-23] | Vermis 6 |
| 0.969 | 5 | [3;-4;-20] | Out of atlas |

Supplementary Table 6: Statistical table with cluster-level inference for parametric map of PPI connectivity in noncanonical condition with seed in striatum for two sample t-test MCI-LB vs HC. Initial cut-off is p<0.001. Shown are up to 3 local maxima per cluster more than 8.0 mm apart. Labels of coordinates are assigned according to AAL atlas^1^.

| PPI noncanonical MCI-LB vs HC two sample t-test | | | |
| --- | --- | --- | --- |
| p_FWE_ corrected | cluster size [N vx] | MNI coordinate [mm] | AAL label |
| <0.001 | 547 | [-6;-73;4] | Lingual L |
|  |  | [-12;-61;-2] | Lingual L |
|  |  | [15;-70;22] | Cuneus R |
| 0.647 | 12 | [48;26;7] | Frontal Inf Tri R |
| 0.007 | 58 | [-57;-55;25] | SupraMarginal L |
|  |  | [-60;-49;19] | Temporal Sup L |
|  |  | [-60;-34;22] | Temporal Sup L |
| 0.861 | 8 | [48;-1;-26] | Temporal Mid R |
| 0.812 | 9 | [36;-13;46] | Precentral R |
| 0.647 | 12 | [54;-25;37] | SupraMarginal R |
| 0.647 | 12 | [51;-22;7] | Temporal Sup R |
| 0.490 | 15 | [21;-85;25] | Occipital Sup R |
| 0.905 | 7 | [6;-7;4] | Out of atlas |
| 0.401 | 17 | [-51;-19;25] | Postcentral L |
| 0.861 | 8 | [9;5;55] | Supp Motor Area R |
| 0.905 | 7 | [-33;-31;-23] | Fusiform L |
| 0.237 | 22 | [51;-46;7] | Temporal Mid R |
| 0.326 | 19 | [3;-46;-20] | Vermis 3 |
|  |  | [3;-61;-26] | Vermis 8 |
| 0.968 | 5 | [-21;-28;4] | Thalamus L |
| 0.702 | 11 | [-42;-25;52] | Postcentral L |
| 0.702 | 11 | [21;-40;-8] | ParaHippocampal R |
| 0.861 | 8 | [-36;-1;55] | Precentral L |
| 0.758 | 10 | [-39;-64;-44] | Cerebelum Crus2 L |
| 0.941 | 6 | [12;53;13] | Frontal Sup Medial R |
| 0.861 | 8 | [30;-4;43] | Out of atlas |
| 0.758 | 10 | [-51;-10;1] | Temporal Sup L |
|  |  | [-57;-10;10] | Heschl L |
| 0.905 | 7 | [21;-97;-2] | Calcarine R |
| 0.702 | 11 | [0;-76;-29] | Vermis 7 |
| 0.941 | 6 | [33;-46;-17] | Fusiform R |
| 0.968 | 5 | [9;-28;43] | Cingulum Mid R |
| 0.968 | 5 | [-36;26;-11] | Frontal Inf Orb L |

Supplementary Table 7: Statistical table with cluster-level inference for parametric map of PPI connectivity in noncanonical condition with seed in striatum for one sample t-test for MCI-LB + HC. Initial cut-off is p<0.001. Shown are up to 3 local maxima per cluster more than 8.0 mm apart. Labels of coordinates are assigned according to AAL atlas^1^.

| Effect of PPI noncanonical for HC+MCI-LB - one sample t-test | | | |
| --- | --- | --- | --- |
| p_FWE_ corrected | cluster size [N vx] | MNI coordinate [mm] | AAL label |
| <0.001 | 11935 | [9;-16;10] | Thalamus R |
|  |  | [21;11;1] | Putamen R |
|  |  | [-12;-16;7] | Thalamus L |
| 0.036 | 41 | [-9;8;40] | Cingulum Mid L |
|  |  | [-9;20;31] | Cingulum Mid L |
|  |  | [-12;2;52] | Out of atlas |
| 0.016 | 50 | [-30;-1;46] | Precentral L |
|  |  | [-21;8;43] | Out of atlas |
| 0.001 | 92 | [9;44;22] | Cingulum Ant R |
|  |  | [12;32;19] | Cingulum Ant R |
|  |  | [6;23;34] | Cingulum Mid R |
| 0.133 | 28 | [21;53;1] | Frontal Sup R |
|  |  | [15;47;-2] | Frontal Med Orb R |
|  |  | [21;59;7] | Frontal Sup R |
| 0.502 | 15 | [-9;44;19] | Frontal Sup Medial L |
| <0.001 | 138 | [-42;14;13] | Frontal Inf Oper L |
|  |  | [-33;8;25] | Frontal Inf Oper L |
|  |  | [-33;20;22] | Out of atlas |
| 0.023 | 46 | [45;-1;31] | Precentral R |
|  |  | [36;8;34] | Frontal Inf Oper R |
|  |  | [33;17;31] | Out of atlas |
| 0.907 | 7 | [-6;-13;58] | Supp Motor Area L |
| 0.059 | 36 | [45;-10;-20] | Out of atlas |
| 0.305 | 20 | [-21;44;-11] | Frontal Sup Orb L |
| 0.656 | 12 | [54;-1;16] | Rolandic Oper R |
| 0.456 | 16 | [36;-10;-14] | Hippocampus R |
|  |  | [33;-4;-8] | Putamen R |
| 0.456 | 16 | [-30;38;22] | Frontal Mid L |
|  |  | [-21;41;19] | Out of atlas |
| 0.147 | 27 | [33;-19;40] | Out of atlas |
|  |  | [33;-28;43] | Out of atlas |
|  |  | [39;-7;46] | Precentral R |
| 0.711 | 11 | [-39;-31;34] | Out of atlas |
| 0.907 | 7 | [27;-25;13] | Out of atlas |
| 0.338 | 19 | [24;41;28] | Frontal Mid R |
|  |  | [24;41;19] | Out of atlas |
| 0.765 | 10 | [-18;26;37] | Frontal Sup L |
| 0.817 | 9 | [6;-7;40] | Cingulum Mid R |
| 0.711 | 11 | [-21;-31;73] | Postcentral L |
|  |  | [-27;-28;67] | Postcentral L |
| 0.006 | 61 | [6;-10;-17] | Out of atlas |
|  |  | [6;-1;-11] | Out of atlas |
|  |  | [-3;-4;-20] | Out of atlas |
| 0.817 | 9 | [0;38;46] | Frontal Sup Medial L |
| 0.656 | 12 | [-57;11;28] | Frontal Inf Oper L |
|  |  | [-54;20;22] | Frontal Inf Tri L |
| 0.865 | 8 | [45;-4;-2] | Insula R |
|  |  | [42;-13;-2] | Insula R |
| 0.551 | 14 | [-42;5;-8] | Insula L |
|  |  | [-42;14;-8] | Insula L |
| 0.338 | 19 | [33;-40;67] | Postcentral R |
|  |  | [21;-43;70] | Postcentral R |
| 0.968 | 5 | [-15;-34;-17] | Fusiform L |
| 0.817 | 9 | [15;-28;-17] | Out of atlas |

Supplementary Table 8: Statistical table with cluster-level inference for parametric map of PPI connectivity in noncanonical condition with seed in striatum for one sample t-test for MCI-LB. Initial cut-off is p<0.001. Shown are up to 3 local maxima per cluster more than 8.0 mm apart. Labels of coordinates are assigned according to AAL atlas^1^.

| Effect of PPI noncanonical for MCI-LB - one sample t-test | | | |
| --- | --- | --- | --- |
| p_FWE_ corrected | cluster size [N vx] | MNI coordinate [mm] | AAL label |
| 0.098 | 28 | [15;-52;-44] | Cerebelum 9 R |
| <0.001 | 994 | [12;5;-2] | Pallidum R |
|  |  | [-18;-16;7] | Thalamus L |
|  |  | [12;5;7] | Caudate R |
| <0.001 | 81 | [-15;-49;37] | Out of atlas |
|  |  | [-12;-37;55] | Precuneus L |
|  |  | [-18;-34;46] | Out of atlas |
| 0.005 | 55 | [18;-1;46] | Out of atlas |
|  |  | [21;-16;58] | Out of atlas |
|  |  | [15;-22;58] | Out of atlas |
| <0.001 | 212 | [-24;-76;-26] | Cerebelum Crus1 L |
|  |  | [-27;-64;-26] | Cerebelum 6 L |
|  |  | [-15;-70;-23] | Cerebelum 6 L |
| 0.049 | 34 | [24;-61;-32] | Cerebelum 6 R |
|  |  | [30;-61;-44] | Cerebelum 8 R |
| 0.021 | 42 | [-18;14;-5] | Putamen L |
|  |  | [-27;11;-2] | Out of atlas |
| 0.098 | 28 | [-45;-49;-17] | Temporal Inf L |
|  |  | [-48;-49;-8] | Out of atlas |
| 0.621 | 12 | [-15;-52;-50] | Cerebelum 9 L |
| 0.506 | 14 | [-18;41;-2] | Out of atlas |
|  |  | [-15;44;-11] | Out of atlas |
| 0.802 | 9 | [-3;8;31] | Cingulum Mid L |
| 0.125 | 26 | [-24;-64;-50] | Cerebelum 8 L |
| 0.802 | 9 | [-6;-58;-50] | Cerebelum 9 L |
| 0.098 | 28 | [-48;-34;-8] | Out of atlas |
|  |  | [-42;-25;-8] | Out of atlas |
|  |  | [-30;-16;-11] | Out of atlas |
| 0.621 | 12 | [18;11;40] | Out of atlas |
| 0.944 | 6 | [-12;-52;-20] | Cerebelum 4 5 L |
| 0.857 | 8 | [-18;23;37] | Frontal Sup L |
|  |  | [-9;20;31] | Cingulum Mid L |
| 0.562 | 13 | [-42;38;-2] | Frontal Inf Tri L |
|  |  | [-33;38;1] | Frontal Inf Tri L |
| 0.454 | 15 | [-21;11;31] | Out of atlas |
|  |  | [-21;8;19] | Out of atlas |
|  |  | [-21;-1;22] | Out of atlas |
| 0.857 | 8 | [-9;-61;-32] | Out of atlas |
| 0.743 | 10 | [-45;-13;19] | Rolandic Oper L |
|  |  | [-36;-7;16] | Rolandic Oper L |
| 0.002 | 67 | [-24;-82;10] | Occipital Mid L |
|  |  | [-24;-73;16] | Out of atlas |
|  |  | [-18;-79;16] | Out of atlas |
| 0.944 | 6 | [-45;-22;25] | Out of atlas |
| 0.621 | 12 | [45;-1;28] | Out of atlas |
|  |  | [39;5;19] | Out of atlas |
| 0.286 | 19 | [21;-64;49] | Parietal Sup R |
|  |  | [18;-67;40] | Precuneus R |
| 0.857 | 8 | [-36;-28;34] | Out of atlas |
| 0.857 | 8 | [42;-61;-11] | Temporal Inf R |
| 0.944 | 6 | [27;-25;13] | Out of atlas |
| 0.905 | 7 | [-3;-58;-23] | Vermis 6 |
|  |  | [3;-55;-14] | Vermis 4 5 |
| 0.621 | 12 | [18;-34;55] | Out of atlas |
| 0.944 | 6 | [12;-25;-5] | Out of atlas |
| 0.905 | 7 | [33;-76;-5] | Out of atlas |
| 0.944 | 6 | [-6;-61;55] | Precuneus L |
| 0.972 | 5 | [12;-58;-23] | Cerebelum 6 R |
| 0.200 | 22 | [0;2;-11] | Out of atlas |
|  |  | [0;-7;-14] | Out of atlas |
|  |  | [0;-4;-5] | Out of atlas |
| 0.905 | 7 | [57;23;10] | Frontal Inf Tri R |
|  |  | [54;26;22] | Frontal Inf Tri R |
| 0.944 | 6 | [27;-37;67] | Postcentral R |

Supplementary Table 9: Statistical table with cluster-level inference for parametric map of PPI connectivity in noncanonical condition with seed in striatum for one sample t-test for HC. Initial cut-off is p<0.001. Shown are up to 3 local maxima per cluster more than 8.0 mm apart. Labels of coordinates are assigned according to AAL atlas^1^.

| Effect of PPI noncanonical for HC - one sample t-test | | | |
| --- | --- | --- | --- |
| p_FWE_ corrected | cluster size [N vx] | MNI coordinate [mm] | AAL label |
| <0.001 | 8401 | [-12;-70;4] | Lingual L |
|  |  | [21;11;1] | Putamen R |
|  |  | [6;-13;10] | Thalamus R |
| <0.001 | 104 | [3;-28;-29] | Out of atlas |
|  |  | [-12;-31;-32] | Out of atlas |
|  |  | [-6;-34;-38] | Out of atlas |
| 0.127 | 25 | [9;5;55] | Supp Motor Area R |
| <0.001 | 89 | [-30;-1;46] | Precentral L |
|  |  | [-33;2;37] | Precentral L |
|  |  | [-39;-10;49] | Precentral L |
| <0.001 | 122 | [33;-19;43] | Precentral R |
|  |  | [36;8;37] | Frontal Mid R |
|  |  | [30;-4;43] | Out of atlas |
| 0.049 | 33 | [48;-1;-29] | Temporal Mid R |
|  |  | [48;-10;-20] | Out of atlas |
| <0.001 | 140 | [-42;11;19] | Frontal Inf Oper L |
|  |  | [-27;23;7] | Insula L |
|  |  | [-33;26;-2] | Frontal Inf Tri L |
| 0.266 | 19 | [-9;8;40] | Cingulum Mid L |
| 0.022 | 40 | [9;44;22] | Cingulum Ant R |
|  |  | [12;29;25] | Cingulum Ant R |
|  |  | [9;32;34] | Cingulum Mid R |
| 0.266 | 19 | [63;-7;13] | Rolandic Oper R |
|  |  | [63;-16;4] | Temporal Sup R |
| 0.381 | 16 | [21;-13;-8] | Out of atlas |
|  |  | [12;-22;-5] | Out of atlas |
| 0.967 | 5 | [-9;44;22] | Frontal Sup Medial L |
| 0.721 | 10 | [45;-25;25] | Out of atlas |
|  |  | [45;-28;34] | Postcentral R |
| 0.842 | 8 | [-12;2;55] | Out of atlas |
| 0.039 | 35 | [-54;-7;13] | Postcentral L |
|  |  | [-54;-16;-2] | Temporal Mid L |
| 0.936 | 6 | [-24;44;-11] | Frontal Sup Orb L |
| 0.967 | 5 | [24;53;-2] | Frontal Sup R |
| 0.658 | 11 | [24;41;-14] | Frontal Sup Orb R |
| 0.127 | 25 | [-30;-22;52] | Precentral L |
|  |  | [-45;-22;52] | Postcentral L |
|  |  | [-21;-22;55] | Out of atlas |
| 0.338 | 17 | [54;-25;37] | SupraMarginal R |
|  |  | [51;-16;25] | SupraMarginal R |
| 0.967 | 5 | [54;-13;1] | Temporal Sup R |
| 0.894 | 7 | [-3;-58;61] | Precuneus L |
| 0.967 | 5 | [-51;2;13] | Rolandic Oper L |
| 0.936 | 6 | [33;-7;-14] | Amygdala R |
| 0.967 | 5 | [-33;-73;40] | Parietal Inf L |
| 0.967 | 5 | [24;23;-11] | Out of atlas |
| 0.338 | 17 | [6;-10;-17] | Out of atlas |
|  |  | [0;-1;-8] | Out of atlas |
| 0.894 | 7 | [3;50;37] | Frontal Sup Medial L |
|  |  | [-6;56;34] | Frontal Sup Medial L |

Code S1: *compute_FDR_ROI_analysis_age_reg.m* Code used for FDR correction.

function [pID,pN] = compute_FDR_ROI_analysis_age_reg(p,q)

% FORMAT [pID,pN] = FDR(p,q)

%

% p - vector of p-values

% q - False Discovery Rate level

%

% pID - p-value threshold based on independence or positive dependence

% pN - Nonparametric p-value threshold

%______________________________________________________________________________

% $Id: FDR.m,v 1.1 2009/10/20 09:04:30 nichols Exp $

p = sort(p(:));

V = length(p);

I = (1:V)';

cVID = 1;

cVN = sum(1./(1:V));

pID = p(max(find(p<=I/V*q/cVID)));

pN = p(max(find(p<=I/V*q/cVN)));

%figure,plot(I/V*q/cVID)

%hold on, plot(p,'r')

%axis([0,length(p_vals),0,0.06])

Code S2: *F_00_create_VOIs_for_filtered_data.m* Code used for extraction of the signal from VOI.

function F_00_create_VOIs_for_filtered_data

input=load('subject_data.mat');

foldname=input.foldname;

task1='task1';

task2='task2';

subject_info_task1=filter_data_to_task(foldname,task1);

subject_info_task2=filter_data_to_task(foldname,task2);

nsub=size(subject_info_task1,1);

task1_error_cell=cell(0,2);

task2_error_cell=cell(0,2);

for sub=1:nsub

compute_parpool(sub,subject_info_task1)

compute_parpool(sub,subject_info_task2)

end

task1_error_cell

task2_error_cell

function subject_info_task=filter_data_to_task(subject_info_run1,task)

task_col=5;

N=size(subject_info_run1,1);

vect=zeros(N,1);

for i=1:N

tmp=subject_info_run1{i,task_col};

if strcmp(tmp,task)

vect(i,1)=1;

end

end

ind=find(vect==1);

subject_info_task=subject_info_run1(ind,:);

function compute_parpool(sub,foldname)

scan_col=4;

ses_col=2;

file_col=3;

name_col=1;

task_name_col=5;

data_filt='cwmr24hpswtse_r';

pathdef=load('paths.mat');

cwd = pathdef.cwd;

mask_root=pathdef.mask_root;

data_folder=pathdef.data_folder;

seed_VOI_path=pathdef.seed_VOI_path;

mask_subfolder='masks';

sesname = foldname{sub,ses_col};

VOI_path=fullfile(cwd,'PPI','Extracted_filtered_VOIs_cwmr24hp');

mkdir(VOI_path)

VOI_input=load('vois.mat');

VOI_cell=VOI_input.VOI_cell;

nVOI=size(VOI_cell,1);

VOI_BOLD=cell(size(VOI_cell,1),size(foldname,1));

nsub=size(foldname,1);

swd = fullfile(data_folder,foldname{sub,name_col},sesname);

cd(swd);

mask_path=fullfile(mask_root,mask_subfolder,['mask_',foldname{sub,name_col},'_',foldname{sub,task_name_col},'.nii']);

q = fullfile(swd,[data_filt,foldname{sub,file_col}]);

V = spm_vol(q);

[Y XYZ] = spm_read_vols(V);

tmp_name=q(1,:);

tmp_vol=spm_vol(tmp_name);

[tmp_img,tmp_ind] = spm_read_vols(tmp_vol);

coords=cell(nVOI,1);

flags.prefix=fullfile(cwd,'PPI','tmp1');

for i=1:nVOI

% Get vx values of positions

tmp_VOI_path=fullfile(seed_VOI_path,VOI_cell{i,2});

P{1} = [q(1,:),',1'];

P{2} = [tmp_VOI_path,',1'];

Pi=strvcat(P{1}, P{2});

spm_unlink([flags.prefix,'.nii'])

[Qo,VOI_vol] = spm_reslice_MG(Pi,flags);

[VOI,~] = spm_read_vols(VOI_vol);

coords{i}.ind=find(VOI==1);

VOI_BOLD{i,sub}.coords=coords{i};

end

P{1} = q(1,:);

P{2} = mask_path;

Pi=strvcat(P{1}, P{2});

flags.prefix=fullfile(cwd,'PPI','tmp');

spm_unlink([flags.prefix,'.nii'])

[Qo,GMSK_vol] = spm_reslice_MG(Pi,flags);

[GMSK,GMSK_mxv] = spm_read_vols(GMSK_vol);

Q2 = find(GMSK);

for VOI_id=1:nVOI

Q1 = coords{VOI_id}.ind;

Q3 = Q1;

Q3(~ismember(Q1,Q2))=[];

maskXYZ = XYZ(:,Q3);

mx=[maskXYZ; ones(1,size(maskXYZ,2))];

mxv=V(1).mat\mx;

mxv=mxv(1:3,:);

VOI_BOLD{VOI_id,sub}.data = spm_get_data(V,mxv);

VOI_BOLD{VOI_id,sub}.desc = VOI_cell{VOI_id,1};

VOI_BOLD{VOI_id,sub}.nr = VOI_id;

VOI_BOLD{VOI_id,sub}.pos_data_mm = maskXYZ;

VOI_BOLD{VOI_id,sub}.pos_data_ind = Q3;

VOI_BOLD{VOI_id,sub}.pos_data_BOLD_vx = mxv;

Y_signals=VOI_BOLD{VOI_id,sub}.data;

[m,n] = size(Y_signals);

Y_signals = spm_detrend(Y_signals,1);

if m > n

[v s v] = svd(Y_signals'*Y_signals);

s = diag(s); % s - eigenvalues

v = v(:,1);

u = Y_signals*v/sqrt(s(1));

else

[u s u] = svd(Y_signals*Y_signals');

s = diag(s); % s - eigenvalues

u = u(:,1);

v = Y_signals'*u/sqrt(s(1));

end

d = sign(sum(v));

u = u*d;

v = v*d;

Y = u*sqrt(s(1)/n);

VOI_BOLD{VOI_id,sub}.first_eigenvector = Y;

clear xY

xY.u=Y;

VOI_name=['VOI_',VOI_cell{VOI_id,1},'_',foldname{sub},'_',foldname{sub,task_name_col}];

save(fullfile(VOI_path,VOI_name),'xY')

end

disp(['**************************'])

disp(['Subject ',num2str(sub),'/',num2str(nsub),' completed'])

function [resliced_name,resliced_vol]=spm_reslice_MG(P,flags)

% Function spm_reslice edited by MG 23.1.2015. Changed type of saving of

% resliced image. All images are resliced to the first image.

%

%__________________________________________________________________________

%

% function spm_reslice(P,flags)

% Rigid body reslicing of images

% FORMAT spm_reslice(P,flags)

%

% P - matrix or cell array of filenames {one string per row}

% All operations are performed relative to the first image.

% ie. Coregistration is to the first image, and resampling

% of images is into the space of the first image.

%

% flags - a structure containing various options. The fields are:

%

% mask - mask output images (true/false) [default: true]

% To avoid artifactual movement-related variance the

% realigned set of images can be internally masked, within

% the set (i.e. if any image has a zero value at a voxel

% than all images have zero values at that voxel). Zero

% values occur when regions 'outside' the image are moved

% 'inside' the image during realignment.

%

% mean - write mean image (true/false) [default: true]

% The average of all the realigned scans is written to

% an image file with 'mean' prefix.

%

% interp - the B-spline interpolation method [default: 1]

% Non-finite values result in Fourier interpolation. Note

% that Fourier interpolation only works for purely rigid

% body transformations. Voxel sizes must all be identical

% and isotropic.

%

% which - values of 0, 1 or 2 are allowed [default: 2]

% 0 - don't create any resliced images.

% Useful if you only want a mean resliced image.

% 1 - don't reslice the first image.

% The first image is not actually moved, so it may

% not be necessary to resample it.

% 2 - reslice all the images.

% If which is a 2-element vector, flags.mean will be set

% to flags.which(2).

%

% wrap - three values of either 0 or 1, representing wrapping in

% each of the dimensions. For fMRI, [1 1 0] would be used.

% For PET, it would be [0 0 0]. [default: [0 0 0]]

%

% prefix - prefix for resliced images [default: 'r']

%

%__________________________________________________________________________

%

% The spatially realigned images are written to the original subdirectory

% with the same (prefixed) filename. They are all aligned with the first.

%

% Inputs:

% A series of images conforming to SPM data format (see 'Data Format'). The

% relative displacement of the images is stored in their header.

%

% Outputs:

% The routine uses information in their headers and writes the realigned

% image files to the same subdirectory with a prefix.

%__________________________________________________________________________

% Copyright (C) 1999-2011 Wellcome Trust Centre for Neuroimaging

% John Ashburner

% $Id: spm_reslice.m 4490 2011-09-14 16:22:27Z guillaume $

%__________________________________________________________________________

%

% The headers of the images contain a 4x4 affine transformation matrix 'M',

% usually affected by the `realignment' and `coregistration' modules.

% What these matrices contain is a mapping from the voxel coordinates

% (x0,y0,z0) (where the first voxel is at coordinate (1,1,1)), to

% coordinates in millimeters (x1,y1,z1).

%

% x1 = M(1,1)*x0 + M(1,2)*y0 + M(1,3)*z0 + M(1,4)

% y1 = M(2,1)*x0 + M(2,2)*y0 + M(2,3)*z0 + M(2,4)

% z1 = M(3,1)*x0 + M(3,2)*y0 + M(3,3)*z0 + M(3,4)

%

% Assuming that image1 has a transformation matrix M1, and image2 has a

% transformation matrix M2, the mapping from image1 to image2 is: M2\M1

% (ie. from the coordinate system of image1 into millimeters, followed

% by a mapping from millimeters into the space of image2).

%

% Several spatial transformations (realignment, coregistration,

% normalisation) can be combined into a single operation (without the

% necessity of resampling the images several times).

%__________________________________________________________________________

%

% Refs:

%

% Friston KJ, Williams SR, Howard R Frackowiak RSJ and Turner R (1995)

% Movement-related effect in fMRI time-series. Mag. Res. Med. 35:346-355

%

% W. F. Eddy, M. Fitzgerald and D. C. Noll (1996) Improved Image

% Registration by Using Fourier Interpolation. Mag. Res. Med. 36(6):923-931

%

% R. W. Cox and A. Jesmanowicz (1999) Real-Time 3D Image Registration

% for Functional MRI. Mag. Res. Med. 42(6):1014-1018

%__________________________________________________________________________

SVNid = '$Rev: 4490 $';

%-Say hello

%--------------------------------------------------------------------------

SPMid = spm('FnBanner',mfilename,SVNid);

% MG 23.1.2015

%--------------------------------------------------------------------------

flags.mask=0;

flags.mean=0;

flags.interp=4;

flags.which=1;

flags.wrap=[1 1 0];

%--------------------------------------------------------------------------

%-Parameters

%--------------------------------------------------------------------------

if ~nargin || isempty(P), P = spm_select([2 Inf],'image'); end

if iscellstr(P), P = char(P); end

if ischar(P), P = spm_vol(P); end

def_flags = spm_get_defaults('realign.write');

% MG 23.1.2015

%--------------------------------------------------------------------------

% def_flags.prefix = 'r';

def_flags.prefix = flags.prefix;

%--------------------------------------------------------------------------

if nargin < 2

flags = def_flags;

else

fnms = fieldnames(def_flags);

for i=1:length(fnms)

if ~isfield(flags,fnms{i})

flags.(fnms{i}) = def_flags.(fnms{i});

end

end

end

if numel(flags.which) == 2

flags.mean = flags.which(2);

flags.which = flags.which(1);

elseif ~isfield(flags,'mean')

flags.mean = 1;

end

%-Reslice

%--------------------------------------------------------------------------

reslice_images(P,flags);

% MG 23.1.2015

%--------------------------------------------------------------------------

resliced_name=[flags.prefix,'.nii'];

resliced_vol=spm_vol(resliced_name);

%--------------------------------------------------------------------------

fprintf('%-40s: %30s\n','Completed',spm('time')) %-#

%==========================================================================

%-function reslice_images(P,flags)

%==========================================================================

function reslice_images(P,flags)

% Reslice images volume by volume

% FORMAT reslice_images(P,flags)

% See main function for a description of the input parameters

if ~isfinite(flags.interp), % Use Fourier method

% Check for non-rigid transformations in the matrixes

for i=1:numel(P)

pp = P(1).mat\P(i).mat;

if any(abs(svd(pp(1:3,1:3))-1)>1e-7)

fprintf('\n Zooms or shears appear to be needed');

fprintf('\n (probably due to non-isotropic voxels).');

fprintf('\n These can not yet be done using the');

fprintf('\n Fourier reslicing method. Switching to');

fprintf('\n 7th degree B-spline interpolation instead.\n\n');

flags.interp = 7;

break

end

end

end

if flags.mask || flags.mean

spm_progress_bar('Init',P(1).dim(3),'Computing available voxels','planes completed');

x1 = repmat((1:P(1).dim(1))',1,P(1).dim(2));

x2 = repmat( 1:P(1).dim(2) ,P(1).dim(1),1);

if flags.mean

Count = zeros(P(1).dim(1:3));

Integral = zeros(P(1).dim(1:3));

end

if flags.mask, msk = cell(P(1).dim(3),1); end;

for x3 = 1:P(1).dim(3)

tmp = zeros(P(1).dim(1:2));

for i = 1:numel(P)

tmp = tmp + getmask(inv(P(1).mat\P(i).mat),x1,x2,x3,P(i).dim(1:3),flags.wrap);

end

if flags.mask, msk{x3} = find(tmp ~= numel(P)); end;

if flags.mean, Count(:,:,x3) = tmp; end;

spm_progress_bar('Set',x3);

end

end

nread = numel(P);

if ~flags.mean

if flags.which == 1, nread = nread - 1; end;

if flags.which == 0, nread = 0; end;

end

spm_progress_bar('Init',nread,'Reslicing','volumes completed');

[x1,x2] = ndgrid(1:P(1).dim(1),1:P(1).dim(2));

nread = 0;

d = [flags.interp*[1 1 1]' flags.wrap(:)];

for i = 1:numel(P)

if (i>1 && flags.which==1) || flags.which==2

write_vol = 1;

else

write_vol = 0;

end

if write_vol || flags.mean

read_vol = 1;

else

read_vol = 0;

end

if read_vol

if ~isfinite(flags.interp)

v = abs(kspace3d(spm_bsplinc(P(i),[0 0 0 ; 0 0 0]'),P(1).mat\P(i).mat));

for x3 = 1:P(1).dim(3)

if flags.mean

Integral(:,:,x3) = ...

Integral(:,:,x3) + ...

nan2zero(v(:,:,x3) .* ...

getmask(inv(P(1).mat\P(i).mat),x1,x2,x3,P(i).dim(1:3),flags.wrap));

end

if flags.mask

tmp = v(:,:,x3); tmp(msk{x3}) = NaN; v(:,:,x3) = tmp;

end

end

else

C = spm_bsplinc(P(i), d);

v = zeros(P(1).dim);

for x3 = 1:P(1).dim(3)

[tmp,y1,y2,y3] = getmask(inv(P(1).mat\P(i).mat),x1,x2,x3,P(i).dim(1:3),flags.wrap);

v(:,:,x3) = spm_bsplins(C, y1,y2,y3, d);

% v(~tmp) = 0;

if flags.mean

Integral(:,:,x3) = Integral(:,:,x3) + nan2zero(v(:,:,x3));

end

if flags.mask

tmp = v(:,:,x3); tmp(msk{x3}) = NaN; v(:,:,x3) = tmp;

end

end

end

if write_vol

VO = P(i);

try

VO.fname = spm_file([flags.prefix,'.nii']); % Změna - definice místa uložení

catch

VO.fname = spm_file(P(i).fname, 'prefix',flags.prefix);

end

VO.dim = P(1).dim(1:3);

VO.dt = P(i).dt;

% VO.dt = [spm_type('int16') P(i).dt(2)];

VO.pinfo = P(i).pinfo;

VO.mat = P(1).mat;

VO.descrip = 'spm - realigned';

VO = spm_write_vol(VO,v);

end

nread = nread + 1;

end

spm_progress_bar('Set',nread);

end

if flags.mean

% Write integral image (16 bit signed)

%----------------------------------------------------------------------

Integral = Integral./Count;

PO = P(1);

PO = rmfield(PO,'pinfo');

PO.fname = spm_file(P(1).fname, 'prefix','mean');

PO.pinfo = [max(max(max(Integral)))/32767 0 0]';

PO.descrip = 'spm - mean image';

PO.dt = [spm_type('int16') spm_platform('bigend')];

spm_write_vol(PO,Integral);

end

spm_progress_bar('Clear');

%==========================================================================

%-function v = kspace3d(v,M)

%==========================================================================

function v = kspace3d(v,M)

% 3D rigid body transformation performed as shears in 1D Fourier space

% FORMAT v = kspace3d(v,M)

% v - image stored as a 3D array

% M - rigid body transformation matrix

%

% v - transformed image

%

% References:

% R. W. Cox and A. Jesmanowicz (1999)

% Real-Time 3D Image Registration for Functional MRI

% Magnetic Resonance in Medicine 42(6):1014-1018

%

% W. F. Eddy, M. Fitzgerald and D. C. Noll (1996)

% Improved Image Registration by Using Fourier Interpolation

% Magnetic Resonance in Medicine 36(6):923-931

[S0,S1,S2,S3] = shear_decomp(M);

d = [size(v) 1 1 1];

g = 2.^ceil(log2(d));

if any(g~=d)

tmp = v;

v = zeros(g);

v(1:d(1),1:d(2),1:d(3)) = tmp;

clear tmp;

end

% XY-shear

tmp1 = -sqrt(-1)*2*pi*([0:((g(3)-1)/2) 0 (-g(3)/2+1):-1])/g(3);

for j=1:g(2)

t = reshape( exp((j*S3(3,2) + S3(3,1)*(1:g(1)) + S3(3,4)).'*tmp1) ,[g(1) 1 g(3)]);

v(:,j,:) = real(ifft(fft(v(:,j,:),[],3).*t,[],3));

end

% XZ-shear

tmp1 = -sqrt(-1)*2*pi*([0:((g(2)-1)/2) 0 (-g(2)/2+1):-1])/g(2);

for k=1:g(3)

t = exp( (k*S2(2,3) + S2(2,1)*(1:g(1)) + S2(2,4)).'*tmp1);

v(:,:,k) = real(ifft(fft(v(:,:,k),[],2).*t,[],2));

end

% YZ-shear

tmp1 = -sqrt(-1)*2*pi*([0:((g(1)-1)/2) 0 (-g(1)/2+1):-1])/g(1);

for k=1:g(3)

t = exp( tmp1.'*(k*S1(1,3) + S1(1,2)*(1:g(2)) + S1(1,4)));

v(:,:,k) = real(ifft(fft(v(:,:,k),[],1).*t,[],1));

end

% XY-shear

tmp1 = -sqrt(-1)*2*pi*([0:((g(3)-1)/2) 0 (-g(3)/2+1):-1])/g(3);

for j=1:g(2)

t = reshape( exp( (j*S0(3,2) + S0(3,1)*(1:g(1)) + S0(3,4)).'*tmp1) ,[g(1) 1 g(3)]);

v(:,j,:) = real(ifft(fft(v(:,j,:),[],3).*t,[],3));

end

if any(g~=d), v = v(1:d(1),1:d(2),1:d(3)); end

%==========================================================================

%-function [S0,S1,S2,S3] = shear_decomp(A)

%==========================================================================

function [S0,S1,S2,S3] = shear_decomp(A)

% Decompose rotation and translation matrix A into shears S0, S1, S2 and

% S3, such that A = S0*S1*S2*S3. The original procedure is documented in:

% R. W. Cox and A. Jesmanowicz (1999)

% Real-Time 3D Image Registration for Functional MRI

% Magnetic Resonance in Medicine 42(6):1014-1018

A0 = A(1:3,1:3);

if any(abs(svd(A0)-1)>1e-7), error('Can''t decompose matrix'); end

t = A0(2,3); if t==0, t=eps; end

a0 = pinv(A0([1 2],[2 3])')*[(A0(3,2)-(A0(2,2)-1)/t) (A0(3,3)-1)]';

S0 = [1 0 0; 0 1 0; a0(1) a0(2) 1];

A1 = S0\A0; a1 = pinv(A1([2 3],[2 3])')*A1(1,[2 3])'; S1 = [1 a1(1) a1(2); 0 1 0; 0 0 1];

A2 = S1\A1; a2 = pinv(A2([1 3],[1 3])')*A2(2,[1 3])'; S2 = [1 0 0; a2(1) 1 a2(2); 0 0 1];

A3 = S2\A2; a3 = pinv(A3([1 2],[1 2])')*A3(3,[1 2])'; S3 = [1 0 0; 0 1 0; a3(1) a3(2) 1];

s3 = A(3,4)-a0(1)*A(1,4)-a0(2)*A(2,4);

s1 = A(1,4)-a1(1)*A(2,4);

s2 = A(2,4);

S0 = [[S0 [0 0 s3]'];[0 0 0 1]];

S1 = [[S1 [s1 0 0]'];[0 0 0 1]];

S2 = [[S2 [0 s2 0]'];[0 0 0 1]];

S3 = [[S3 [0 0 0]'];[0 0 0 1]];

%==========================================================================

%-function [Mask,y1,y2,y3] = getmask(M,x1,x2,x3,dim,wrp)

%==========================================================================

function [Mask,y1,y2,y3] = getmask(M,x1,x2,x3,dim,wrp)

tiny = 5e-2; % From spm_vol_utils.c

y1 = M(1,1)*x1+M(1,2)*x2+(M(1,3)*x3+M(1,4));

y2 = M(2,1)*x1+M(2,2)*x2+(M(2,3)*x3+M(2,4));

y3 = M(3,1)*x1+M(3,2)*x2+(M(3,3)*x3+M(3,4));

Mask = true(size(y1));

if ~wrp(1), Mask = Mask & (y1 >= (1-tiny) & y1 <= (dim(1)+tiny)); end

if ~wrp(2), Mask = Mask & (y2 >= (1-tiny) & y2 <= (dim(2)+tiny)); end

if ~wrp(3), Mask = Mask & (y3 >= (1-tiny) & y3 <= (dim(3)+tiny)); end

%==========================================================================

%-function vo = nan2zero(vi)

%==========================================================================

function vo = nan2zero(vi)

vo = vi;

vo(~isfinite(vo)) = 0;

Code S3: *F_01_compute_PPI_regressors.m* Code used for calculation of PPI regressors.

function F_01_compute_PPI_regressors

input='subjects.mat';

foldname=input.subject_data;

task1='task1';

task2='task2';

subject_info_task1=filter_data_to_task(foldname,task1);

subject_info_task2=filter_data_to_task(foldname,task2);

nsub=size(subject_info_task1,1);

foldname2=subject_info_task1(:,1);

comparison_cell = {

[1 1 0 0 0] 'Canonical'

[0 0 1 1 0] 'Noncanonical'

};

ncompar = size(comparison_cell,1);

for sub=1:nsub

tic

for i=1:ncompar

compute_parpool(sub,foldname2,comparison_cell(i,:),subject_info_task1,subject_info_task2)

end

disp(['**************************'])

disp(['Subject ',num2str(sub),'/',num2str(nsub),' completed'])

toc

end

function subject_info_task=filter_data_to_task(subject_info_run1,task)

task_col=5;

N=size(subject_info_run1,1);

vect=zeros(N,1);

for i=1:N

tmp=subject_info_run1{i,task_col};

if strcmp(tmp,task)

vect(i,1)=1;

end

end

ind=find(vect==1);

subject_info_task=subject_info_run1(ind,:);

function compute_parpool(sub,foldname,comparison_cell,foldname_orig1,foldname_orig2)

scan_col=4;

ses_col=2;

task_name_col=5;

foldname_all{1,1}=foldname_orig1;

foldname_all{2,1}=foldname_orig2;

pathdef=load('paths.mat');

cwd = pathdef.cwd;

SPM_path=fullfile(cwd,'GLM');

VOI_input=load('vois.mat');

VOI_cell=VOI_input.VOI_cell;

task_names={

'task_1'

'task_2'

};

nVOI=size(VOI_cell,1);

nses=size(task_names,1);

nROI=size(VOI_cell,1);

SPMname=fullfile(SPM_path,foldname{sub,1},'Model_1','SPM.mat');

ppiflag='ppi';

showGraphics=0;

load(SPMname)

ppiname_path=fullfile(cwd,'PPI','ppi_reg',comparison_cell{1,2});

mkdir(ppiname_path)

for i=1:nVOI

for ses = 1:nses

clear U

U = prepare_U(SPM,comparison_cell,ses);

VOI_name=['VOI_',VOI_cell{i,1},'_',foldname{sub},'_',foldname_all{ses,1}{sub,task_name_col},'.mat'];

VOI=fullfile(cwd,'PPI','Extracted_filtered_VOIs_cwmr24hp',VOI_name);

ppiname=['ppi_reg_VOI_',VOI_cell{i,1},'_',foldname{sub},'_ses_',num2str(ses)];

PPI = spm_peb_ppiMB_noncentr_gPPI12(SPMname,ppiflag,VOI,U,ppiname,showGraphics,ppiname_path,ses);

end

end

function U = prepare_U(SPM,comparison_cell,ses)

contrast_vect=comparison_cell{1};

N=numel(contrast_vect);

offset=33;

tmp=[];

for i=1:N

U.name(i) = {SPM.Sess(ses).U(i).name};

tmp=[tmp,SPM.Sess(ses).U(i).u(offset:end,1)];

end

U.u = tmp;

U.w = contrast_vect;

function PPI = spm_peb_ppiMB_noncentr_gPPI12(varargin)

% Bold deconvolution to create physio- or psycho-physiologic interactions

% FORMAT PPI = spm_peb_ppi(SPMname,ppiflag,VOI,Uu,ppiname,showGraphics)

%

% SPM - Structure containing generic details about the analysis or

% the fully qualified filename of such a structure.

% ppiflag - Type of analysis. Must be one of:

% 'simple deconvolution' or 'sd'

% 'psychophysiologic interaction' or 'ppi'

% 'physiophysiologic interaction' or 'phipi'

% VOI - Structure containing details about a VOI (as produced by

% spm_regions) or the fully qualified filename of such a

% structure. If a structure, then VOI should be of size 1x1

% in the case of simple deconvolution, and psychophysiologic

% interactions) or 1x2, in the case of physiophysiologic

% interactions. If a file name it should be 1xN or 2xN.

% Uu - Matrix of input variables and contrast weights. This is an

% [n x 3] matrix. The first column indexes SPM.Sess.U(i). The

% second column indexes the name of the input or cause, see

% SPM.Sess.U(i).name{j}. The third column is the contrast

% weight. Unless there are parametric effects the second

% column will generally be a 1.

% ppiname - Basename of the PPI file to save. The saved file will be:

% <PATH_TO_SPM.MAT>/PPI_<ppiname>.mat

% showGraphics - empty or 1 = yes, 0 = no.

%

%

% PPI.ppi - (PSY*xn or xn1*xn2) convolved with the HRF

% PPI.Y - Original BOLD eigenvariate. Use as covariate of no interest

% PPI.P - PSY convolved with HRF for psychophysiologic interactions,

% or in the case of physiophysologic interactions contains

% the eigenvariate of the second region.

% PPI.name - Name of PPI

% PPI.xY - Original VOI information

% PPI.xn - Deconvolved neural signal(s)

% PPI.U.u - Psychological variable or input function (PPIs only)

% PPI.U.w - Contrast weights for psychological variable (PPIs only)

% PPI.U.name - Names of psychological conditions (PPIs only)

%__________________________________________________________________________

%

% This routine is effectively a hemodynamic deconvolution using full priors

% and EM to deconvolve the HRF from a hemodynamic time series to give a

% neuronal time series [that can be found in PPI.xn]. This deconvolution

% conforms to Wiener filtering. The neuronal process is then used to form

% PPIs. See help text within function for more details.

%__________________________________________________________________________

% Copyright (C) 2002-2011 Wellcome Trust Centre for Neuroimaging

% Darren Gitelman

% $Id: spm_peb_ppi.m 4306 2011-04-13 17:02:00Z guillaume $

% SETTING UP A PPI THAT ACCOUNTS FOR THE HRF

% =========================================================================

% PPI's were initially conceived as a means of identifying regions whose

% reponses can be explained in terms of an interaction between activity in

% a specified source (the physiological factor) and some experimental

% effect (the psychological factor). However, a problem in setting up PPI's

% is that in order to derive a proper estimate of the interaction between

% a psychological variable (P) and measured hemodynamic signal (x), one

% cannot simply convolve the psychological variable with the hrf (HRF) and

% multiply by the signal. Thus:

%

% conv(P,HRF).* x ~= conv((P.*xn),HRF)

%

% P = psychological variable

% HRF = hemodynamic response function

% xn = underlying neural signal which in fMRI is convolved with the hrf to

% give the signal one measures -- x.

% x = measured fmri signal

%

% It is actually the right hand side of the equation one wants.

% Thus one has to work backwards, in a sense, and deconvolve the hrf

% from x to get xn. This can then be multiplied by P and the resulting

% vector (or matrix) reconvolved with the hrf.

%

% This algorithm uses a least squares strategy to solve for xn.

%

% The source's hemodynamics are x = HRF*xn;

%

% Using the constraint that xn should have a uniform spectral density

% we can expand x in terms of a discrete cosine set (xb)

%

% xn = xb*B

% B = parameter estimate

%

% The estimator of x is then

%

% x = HRF(k,:)*xn

% x = HRF(k,:) * xb * B

%

% This accounts for different time resolutions between our hemodynamic

% signal and the discrete representation of the psychological variable. In

% this case k is a vector representing the time resolution of the scans.

%

% Conditional estimates of B allow for priors that ensure uniform variance

% over frequencies.

%

% PPI STATISTICAL MODEL

% =========================================================================

% Once the PPI.ppi interaction term has been calculated a new GLM must be

% setup to search for the interaction effects across the brain. This is

% done using a standard, first level, fMRI model, which must include 3

% covariates, PPI.ppi (interaction), PPI.Y (main effect: source region bold

% signal) and PPI.P (main effect: "psychological" condition), plus any

% nuisance regressors according to the particular design.

%

% NB: Designs that include only the interaction term without the main

% effects are not proper as inferences on the interaction will include a

% mixture of both main and interaction effects.

%

% Once the model has been setup and run, a contrast of [1 0 0 ] over the

% PPI.ppi, PPI.Y and PPI.P columns respectively, will show regions with a

% positive relationship to the interaction term, discounting any main

% effects. Negative regressions can be examined with [-1 0 0]. A PPI random

% effects analysis would involve taking the con*.img files from the [1 0 0]

% t-contrast for each subject and forwarding them to a second level

% analysis.

% Set up the graphical interface

%--------------------------------------------------------------------------

Finter = spm_figure('GetWin','Interactive');

header = get(Finter,'Name');

spm_clf(Finter); set(Finter,'name','PPI Setup');

% MG - upgrade for more seesions

try

ses=varargin{8};

catch

ses=1;

end

% Check inputs

%--------------------------------------------------------------------------

if nargin && isstruct(varargin{1})

SPM = varargin{1};

try

swd = SPM.pwd;

catch

swd = '';

end

else

try

P = varargin{1};

catch

[P, sts] = spm_select(1,'^SPM\.mat$','Select SPM.mat');

if ~sts, PPI = struct([]); return; end

end

swd = spm_str_manip(P,'H');

load(fullfile(swd,'SPM.mat'));

end

if isempty(swd) || strcmp(swd,'.'), swd = pwd; end

SPM.swd = swd;

cwd = pwd;

cd(SPM.swd)

% Setup variables

%--------------------------------------------------------------------------

RT = SPM.xY.RT;

dt = SPM.xBF.dt;

NT = RT/dt;

fMRI_T0 = SPM.xBF.T0;

% Ask whether to perform physiophysiologic or psychophysiologic interactions

%--------------------------------------------------------------------------

try

ppiflag = varargin{2};

catch

ppiflag = {'simple deconvolution',...

'psychophysiologic interaction',...

'physiophysiologic interaction'};

i = spm_input('Analysis type?',1,'m',ppiflag);

ppiflag = ppiflag{i};

end

switch lower(ppiflag)

case {'simple deconvolution','sd'}

%======================================================================

if nargin>2 && isstruct(varargin{3})

p.xY = varargin{3};

else

try

VOI = varargin{3};

p = load(deblank(VOI(1,:)),'xY');

catch

spm_input('physiological variable:... ',2,'d');

voi = spm_select(1,'^VOI.*\.mat$',{'select VOI'});

p = load(deblank(voi),'xY');

end

end

xY(1) = p.xY;

Sess = SPM.Sess(xY(1).Sess);

case {'physiophysiologic interaction','phipi'}

%======================================================================

if nargin>2 && isstruct(varargin{3})

xY = varargin{3};

xY = xY(:)';

if size(xY) ~= [1 2]

error('Must include 2 VOI structures for physiophysiologic interactions')

end

else

try

VOI = varargin{3};

if size(VOI,1) ~= 2

error('Must include 2 VOI filenames for physiophygiologic interactions')

end

for i = 1:2

p = load(deblank(VOI(i,:)),'xY');

xY(i) = p.xY;

end

catch

spm_input('physiological variables:... ',2,'d');

voi = spm_select(2,'^VOI.*\.mat$',{'select VOIs'});

for i = 1:2

p = load(deblank(voi(i,:)),'xY');

xY(i) = p.xY;

end

end

end

Sess = SPM.Sess(xY(1).Sess);

case {'psychophysiologic interaction','ppi'}

%======================================================================

if nargin>2 && isstruct(varargin{3})

p.xY = varargin{3};

else

try

VOI = varargin{3};

p = load(deblank(VOI(1,:)),'xY');

catch

spm_input('physiological variable:... ',2,'d');

voi = spm_select(1,'^VOI.*\.mat$',{'select VOI'});

p = load(deblank(voi(:))','xY');

end

end

p.xY.u=(p.xY.u)./(quantile(p.xY.u,0.75)-quantile(p.xY.u,0.25));

xY(1) = p.xY;

Sess = SPM.Sess(ses);%%%%

% get 'causes' or inputs U

%----------------------------------------------------------------------

U=varargin{4};

end

% Name of PPI file to be saved

%--------------------------------------------------------------------------

try

PPI.name = varargin{5};

catch

PPI.name = spm_input('Name of PPI',3,'s','PPI');

end

[tmp ppiFilename] = fileparts(PPI.name);

% Check if Graphical output should be shown

%--------------------------------------------------------------------------

try

showGraphics = varargin{6};

catch

showGraphics = 1;

end

if showGraphics

Fgraph = spm_figure('GetWin','PPI');

spm_clf(Fgraph);

FS = spm('FontSizes');

end

% Setup more variables

%--------------------------------------------------------------------------

N = length(xY(1).u);

k = 1:NT:N*NT; % microtime to scan time indices

% Create basis functions and hrf in scan time and microtime

%--------------------------------------------------------------------------

spm('Pointer','watch')

hrf = spm_hrf(dt);

% Create convolved explanatory {Hxb} variables in scan time

%--------------------------------------------------------------------------

xb = spm_dctmtx(N*NT + 128,N);

Hxb = zeros(N,N);

for i = 1:N

Hx = conv(xb(:,i),hrf);

Hxb(:,i) = Hx(k + 128);

end

xb = xb(129:end,:);

% Get confounds (in scan time) and constant term

%--------------------------------------------------------------------------

% X0 = xY(1).X0;

X0 = SPM.xX.K.X0;

M = size(X0,2);

% Get response variable,

%--------------------------------------------------------------------------

for i = 1:size(xY,2)

Y(:,i) = xY(i).u;

end

% Remove confounds and save Y in ouput structure

%--------------------------------------------------------------------------

Yc = Y - X0*inv(X0'*X0)*X0'*Y;

PPI.Y = Yc(:,1);

if size(Y,2) == 2

PPI.P = Yc(:,2);

end

% Specify covariance components; assume neuronal response is white

% treating confounds as fixed effects

%--------------------------------------------------------------------------

Q = speye(N,N)*N/trace(Hxb'*Hxb);

Q = blkdiag(Q, speye(M,M)*1e6 );

% Get whitening matrix (NB: confounds have already been whitened)

%--------------------------------------------------------------------------

W = SPM.xX.W(Sess.row,Sess.row);

% Create structure for spm_PEB

%--------------------------------------------------------------------------

clear P

P{1}.X = [W*Hxb X0]; % Design matrix for lowest level

P{1}.C = speye(N,N)/4; % i.i.d assumptions

P{2}.X = sparse(N + M,1); % Design matrix for parameters (0's)

P{2}.C = Q;

switch ppiflag

case {'simple deconvolution','sd'}

%======================================================================

C = spm_PEB(Y,P);

xn = xb*C{2}.E(1:N);

xn = spm_detrend(xn);

% Save variables (NOTE: xn is in microtime and does not account for

% slice timing shifts). To convert to BOLD signal convolve with a hrf.

% Use a microtime to scan time index to convert to scan time: e.g.,

% k = 1:NT:N*NT; where NT = number of bins per TR = TR/dt or SPM.xBF.T

% and N = number of scans in the session. Finally account for slice

% timing effects by shifting the index accordingly. See approximately

% lines 420 and 421 below for an example.)

%----------------------------------------------------------------------

PPI.xn = xn;

% Plot results

%----------------------------------------------------------------------

if showGraphics

figure(Fgraph);

t = RT*[1:N];

T = dt*[1:(N*NT)];

ax = subplot(2,1,1);

plot(t,Yc,T,PPI.xn)

title('hemodynamic and neuronal responses')

xlabel('time (secs)')

axis tight square

grid on

legend('BOLD','neuronal')

str = sprintf('Simple Deconvolution: %s\n',ppiFilename);

str = [str sprintf('VOI file: %s',xY.name)];

hAx = axes('Position',[0 0 1 1],...

'DefaultTextFontSize',FS(8),...

'DefaultTextInterpreter','Tex',...

'DefaultTextVerticalAlignment','Baseline',...

'Parent',Fgraph,...

'Units','points',...

'Visible','off');

AxPos = get(hAx,'Position');

set(hAx,'XLim',[0,AxPos(3)]); set(hAx,'YLim',[0,AxPos(4)])

dy = FS(9);

h = text(dy,floor(AxPos(4))-2*dy,str);

set(h,'Parent',hAx);

end

case {'physiophysiologic interaction','phipi'}

%======================================================================

C = spm_PEB(Y(:,1),P);

xn1 = xb*C{2}.E(1:N);

C = spm_PEB(Y(:,2),P);

xn2 = xb*C{2}.E(1:N);

xn1 = spm_detrend(xn1);

xn2 = spm_detrend(xn2);

xnxn = xn1.*xn2;

% Convolve, convert to scan time, and account for slice timing shift

%----------------------------------------------------------------------

ppi = conv(xnxn,hrf);

ppi = ppi((k-1) + fMRI_T0);

% Save variables

%----------------------------------------------------------------------

PPI.xn = [xn1 xn2];

PPI.ppi = spm_detrend(ppi);

% Plot results

%----------------------------------------------------------------------

if showGraphics

figure(Fgraph);

t = RT*[1:N];

T = dt*[1:(N*NT)];

ax = subplot(2,1,1);

plot(t,PPI.ppi)

title('PPI')

xlabel('time (secs)')

axis tight square

grid on

subplot(2,2,3)

plot(t,Yc(:,1),T,PPI.xn(:,1))

title('hemodynamic and neuronal responses (1st)')

xlabel('time (secs)')

axis tight square

grid on

legend('BOLD','neuronal')

subplot(2,2,4)

plot(t,Yc(:,2),T,PPI.xn(:,2))

title('hemodynamic and neuronal responses (2nd)')

xlabel('time (secs)')

axis tight square

grid on

legend('BOLD','neuronal')

str = sprintf('Physiophysiologic Interaction: %s\n',ppiFilename);

str = [str, sprintf('VOI File 1: %s\n',xY(1).name)];

str = [str, sprintf('VOI File 2: %s',xY(2).name)];

hAx = axes('Position',[0 0 1 1],...

'DefaultTextFontSize',FS(8),...

'DefaultTextInterpreter','Tex',...

'DefaultTextVerticalAlignment','Baseline',...

'Parent',Fgraph,...

'Units','points',...

'Visible','off');

AxPos = get(hAx,'Position');

set(hAx,'XLim',[0,AxPos(3)]); set(hAx,'YLim',[0,AxPos(4)])

dy = FS(9);

h = text(dy,floor(AxPos(4))-2*dy,str);

set(h,'Parent',hAx);

end

case {'psychophysiologic interaction','ppi'}

%======================================================================

% COMPUTE PSYCHOPHYSIOLOGIC INTERACTIONS

% use basis set in microtime

%----------------------------------------------------------------------

% get parameter estimates and neural signal; beta (C) is in scan time

% This clever trick allows us to compute the betas in scan time which is

% much quicker than with the large microtime vectors. Then the betas

% are applied to a microtime basis set generating the correct neural

% activity to convolve with the psychological variable in microtime

%----------------------------------------------------------------------

C = spm_PEB(Y,P);

xn = xb*C{2}.E(1:N);

xn = spm_detrend(xn);

% Setup psychological variable from inputs and contast weights

%----------------------------------------------------------------------

PSY = zeros(N*NT,1);

for i = 1:size(U.u,2)

PSY = PSY + full(U.u(:,i)*U.w(:,i));

end

% PSY = spm_detrend(PSY); <- removed centering of psych variable

% prior to multiplication with xn. Based on discussion with Karl

% and Donald McLaren.

% Multiply psychological variable by neural signal

%----------------------------------------------------------------------

PSYxn = PSY.*xn;

% Convolve, convert to scan time, and account for slice timing shift

%----------------------------------------------------------------------

ppi = conv(PSYxn,hrf);

ppi = ppi((k-1) + fMRI_T0);

% Convolve psych effect, convert to scan time, and account for slice

% timing shift

%----------------------------------------------------------------------

PSYHRF = conv(PSY,hrf);

PSYHRF = PSYHRF((k-1) + fMRI_T0);

% Save psychological variables

%----------------------------------------------------------------------

PPI.psy = U;

PPI.P = PSYHRF;

PPI.xn = xn;

PPI.ppi = spm_detrend(ppi);

% Plot results

%----------------------------------------------------------------------

if showGraphics

figure(Fgraph);

t = RT*[1:N];

T = dt*[1:(N*NT)];

str = sprintf('Psychophyiologic Interaction: %s\n',ppiFilename);

str = [str, sprintf('VOI File: %s\n',xY(1).name)];

str = [str, sprintf('Factors: ')];

for i = 1:numel(U.name)

str = [str, sprintf('%s [%0.0f]',U.name{i},U.w(i))];

if i < numel(U.name)

str = [str, sprintf('; ')];

end

end

ax = subplot(2,1,1);

plot(t,Yc(:,1),T,PPI.xn(:,1))

title('hemodynamic and neuronal responses')

xlabel('time (secs)')

axis tight square

grid on

legend('BOLD','neuronal')

subplot(2,2,3)

plot(T,PSY,'LineStyle','--','Color',[0 .65 0]);

hold on

plot(t,PPI.P,'LineStyle','-','LineWidth',1,'Color','b');

hold off

title('[convolved] psych. variable')

xlabel('time (secs)')

axis tight square

grid on

subplot(2,2,4)

plot(t,PPI.ppi)

title('PPI')

xlabel('time (secs)')

axis tight square

grid on

hAx = axes('Position',[0 0 1 1],...

'DefaultTextFontSize',FS(8),...

'DefaultTextInterpreter','None',...

'DefaultTextVerticalAlignment','Baseline',...

'Parent',Fgraph,...

'Units','points',...

'Visible','off');

AxPos = get(hAx,'Position');

set(hAx,'XLim',[0,AxPos(3)]); set(hAx,'YLim',[0,AxPos(4)])

dy = FS(9);

h = text(dy,floor(AxPos(4))-2*dy,str);

set(h,'Parent',hAx);

end

end

% Setup other output variables and Save

%--------------------------------------------------------------------------

PPI.xY = xY;

PPI.dt = dt;

ppiname_name=varargin{5};

ppiname_path=varargin{7};

str = [ ppiname_name '_NONcentr.mat'];

if spm_check_version('matlab','7') >= 0,

save(fullfile(ppiname_path,str),'-V6','PPI')

else

save(fullfile(ppiname_path,str),'PPI')

end

% Clean up

%--------------------------------------------------------------------------

spm('Pointer','arrow'); set(Finter,'name',header);

fprintf('\nCompleted PPI: %s\n',ppiFilename);

cd(cwd);

Code S4: *F_02_Compute_PPI_analysis.m* Code used for PPI analysis.

function F_02_Compute_PPI_analysis

input='subjects.mat';

foldname=input.subject_data;

nsub=size(foldname,1);

VOI_input=load('vois.mat');

VOI_cell=VOI_input.VOI_cell;

nVOI=size(VOI_cell,1);

for sub=1:nsub

for VOI_id=1:nVOI

compute_parpool(sub,foldname,VOI_cell,VOI_id)

end

disp(['**************************'])

disp(['Subject ',num2str(sub),'/',num2str(nsub),' completed'])

end

function compute_parpool(sub,foldname,VOI_cell,VOI_id)

spm('defaults','fmri');

ses_col=2;

pathdef=load('paths.mat');

cwd = pathdef.cwd;

mask_root=pathdef.mask_root;

data_filter_WM_CSF_extraction='wtse_r';

mask_subfolder='masks';

task_names={

'task_1'

'task_2'

};

sesname = foldname{sub,ses_col};

nses=size(task_names,1);

for ses =1:nses

if ses==1

suffix='task1';

elseif ses==2

suffix='task2';

end

fwd = fullfile(cwd,'preprocessed',foldname{sub,1},sesname);

BOLD_filename=fullfile(fwd,foldname{sub,2+ses});

datacwd = fwd;

modelcwd = fullfile(cwd,'PPI','PPI_models');

nsub=size(foldname,1);

disp(' ')

disp(' %%%%%%%%%%%%%%%%%%%%%%%%%%%%%%%%%%%%%%%%%%%%%%%%%%%%%%%%%%%%%%%%%%%%%%%%%%%%% ')

disp(' ')

disp(['Processing sub ',foldname{sub,1}, '; ', num2str(sub),' / ',num2str(nsub),' Ses: ',num2str(ses),' VOI: ',VOI_cell{VOI_id,1}])

disp(' ')

disp(' %%%%%%%%%%%%%%%%%%%%%%%%%%%%%%%%%%%%%%%%%%%%%%%%%%%%%%%%%%%%%%%%%%%%%%%%%%%%% ')

disp(' ')

clear dicom_header

header_path=[BOLD_filename(1:end-4),'.json'];

json_data=spm_jsonread(header_path);

TR=json_data.RepetitionTime;

mrfile=fullfile(fwd,['rp_',foldname{sub,2+ses}(1:end-4),'.txt']);%spm_select('FPList',fullfile(movement_folder),['^rp_',foldname{sub,1},'_',tmp_variant,'_','.*txt']);

orig_string='_echo-1_bold';

new_string='_echo-2_bold';

k1=strfind(mrfile,orig_string);

kN=numel(orig_string);

mrfile(k1:k1+kN-1)=new_string;

rp = spm_detrend(dlmread(mrfile));

rp2=rp.^2;

rp_diff=[zeros(1,size(rp,2));diff(rp)];

rp_diff2=rp_diff.^2;

tmp_path=fullfile(mask_root,['Excluded_scans_report_FD_1_5mm_57_sub.mat']);

tmp_mov_data=load(tmp_path);

tmp_info=tmp_mov_data.excluded_scans(:,3);

fMRI_data_name=foldname{sub,2+ses};

orig_string='_echo-1_bold';

new_string='_echo-2_bold';

k1=strfind(fMRI_data_name,orig_string);

kN=numel(orig_string);

fMRI_data_name(k1:k1+kN-1)=new_string;

fMRI_data_name=fMRI_data_name(1:end-4)

NIFTIinfo = niftiinfo(BOLD_filename);

nscans = NIFTIinfo.raw.dim(5);

are_present_ex_scans_ind = find(ismember(tmp_info, fMRI_data_name));

if ~isempty(are_present_ex_scans_ind)

tmp_excluded = tmp_mov_data.excluded_scans{are_present_ex_scans_ind,4};

tmp_excluded_num=prepare_number_MG(tmp_excluded);

excluded_scans_reg=create_GLM_reg(tmp_excluded_num,nscans);

else

excluded_scans_reg=[];

end

mask_path=fullfile(mask_root,mask_subfolder);

mask_name=['mask_',foldname{sub,1},'_',suffix,'.nii'];

reg_WM_CSF=create_WM_CSF_reg(fwd,foldname{sub,2+ses},data_filter_WM_CSF_extraction,mask_path,mask_name,TR);

reg1=[rp rp2 rp_diff rp_diff2 reg_WM_CSF];

reg1=stand(reg1,1);

reg1=[reg1 excluded_scans_reg];

mrf=reg1;

N_mrf=size(mrf,2);

clear regressstruct

%% Canonical

PPI_1_name=fullfile(cwd,'PPI','ppi_reg','Canonical',['ppi_reg_VOI_',VOI_cell{VOI_id,1},'_',foldname{sub},'_ses_',num2str(ses),'_NONcentr.mat']);

PPI_1_data=load(PPI_1_name);

% Seed signal

seed_Y=PPI_1_data.PPI.xY.u;

% PPI task

PPI_task1=PPI_1_data.PPI.P;

% PPI interaction 1

PPI_interaction1=PPI_1_data.PPI.ppi;

%% Noncanonical

PPI_2_name=fullfile(cwd,'PPI','ppi_reg','Noncanonical',['ppi_reg_VOI_',VOI_cell{VOI_id,1},'_',foldname{sub},'_ses_',num2str(ses),'_NONcentr.mat']);

PPI_2_data=load(PPI_2_name);

% PPI task

PPI_task2=PPI_2_data.PPI.P;

% PPI interaction 2

PPI_interaction2=PPI_2_data.PPI.ppi;

%% Control

PPI_3_name=fullfile(cwd,'PPI','ppi_reg','Control',['ppi_reg_VOI_',VOI_cell{VOI_id,1},'_',foldname{sub},'_ses_',num2str(ses),'_NONcentr.mat']);

PPI_3_data=load(PPI_3_name);

PPI_task3=PPI_3_data.PPI.P;

PPI_interaction3=PPI_3_data.PPI.ppi;

reg=[seed_Y PPI_task1 PPI_interaction1 PPI_task2 PPI_interaction2 PPI_task3 PPI_interaction3];

nregPPI=size(reg,2);

regr_names = {'Seed', ...

'Task canonical',...

'PPI interakce canonical',...

'Task noncanonical',...

'PPI interakce noncanonical', ...

'Task control',...

'PPI interakce control', ...

'x trans', 'y trans', 'z trans', 'x rot', 'y rot', 'z rot',...

'x trans^2', 'y trans^2', 'z trans^2', 'x rot^2', 'y rot^2', 'z rot^2',...

'x trans diff', 'y trans diff', 'z trans diff', 'x rot diff', 'y rot diff', 'z rot diff',...

'x trans diff^2', 'y trans diff^2', 'z trans diff^2', 'x rot diff^2', 'y rot diff^2', 'z rot diff^2',...

'WM',...

'CSF',...

};

%% Seed GLM from spm code

data_filt='swtse_r';

P = [];

q=fullfile(fwd,[data_filt,foldname{sub,2+ses}]);

P = strvcat(P,q);

selected_scans=cell(nscans,1);

for i=1:nscans

selected_scans{i,1} = [P(1,:),',',num2str(i)];

end

mdir = fullfile(modelcwd,foldname{sub});

if ~(exist(mdir,'dir'))

mkdir(mdir);

end

model_dir{1} = {mdir};

ModelDirectoryName=['Seed_',VOI_cell{VOI_id,1}];

mdir = fullfile(modelcwd,foldname{sub},ModelDirectoryName);

if ~(exist(mdir))

mkdir(mdir);

end

cd(mdir);

model_dir{1}={mdir};

timingstruct = struct( 'units', 'scans',...

'RT', TR,...

'fmri_t', 16,...

'fmri_t0', 1);

modstruct = struct( 'name', {},...

'param', {},...

'poly', {});

condstruct = struct( 'name', {},...

'onset', [],...

'duration', [],...

'tmod', 0,...

'pmod', 0);

% Seed signal

for i=1:nregPPI

regressstruct(i) = struct( 'name', regr_names{i},...

'val', reg(:,i));

end

% Movement regressors

for i=nregPPI+1:nregPPI+24

regressstruct(:,i).val = spm_detrend(mrf(:,i-(nregPPI)),0);

regressstruct(:,i).name = regr_names{i};

end

% WM and CSF confounds

for i=nregPPI+25:nregPPI+26

regressstruct(:,i).val = spm_detrend(mrf(:,i-(nregPPI)),0);

regressstruct(:,i).name = regr_names{i};

end

% excluded scans - confounds

tmp_ind1=0;

for i=nregPPI+27:nregPPI+N_mrf

regressstruct(:,i).val = spm_detrend(mrf(:,i-(nregPPI)),0);

tmp_ind1=tmp_ind1+1;

regressstruct(:,i).name = ['Excluded scan ', num2str(tmp_ind1)];

end

sessstruct(ses) = struct( 'scans', {selected_scans},...

'cond', condstruct,...

'multi', {{''}},...

'regress', regressstruct,...

'multireg', {''},...

'hpf', 128);

clear selected_scans s regressstruct

end

factstruct = struct( 'name', {},...

'levels', {});

basesstruct.hrf.derivs = [0 0];

fmrispecstruct= struct( 'dir', model_dir,...

'timing', timingstruct,...

'sess', sessstruct,...

'fact', factstruct,...

'bases', basesstruct,...

'volt', 1,...

'global', 'None',...

'mthresh', 0.8,...

'mask', {{''}},...

'cvi', 'AR(1)');

spm_unlink(fullfile(mdir, 'SPM.mat'));

jobs{1}.stats{1}.fmri_spec = fmrispecstruct;

spm_jobman('run',jobs);

clear jobs;

matfile_path{1} = {fullfile(mdir,'SPM.mat')};

methodstruct.Classical = 1;

fmrieststruct = struct( 'spmmat', matfile_path,...

'method', methodstruct);

spm_unlink(fullfile(mdir, 'mask.img'));

jobs{1}.stats{1}.fmri_est = fmrieststruct;

spm_jobman('run',jobs);

clear jobs;

consessstruct{1}.tcon.name='PPI canonical';

consessstruct{1}.tcon.weights=[0 0 1 0 0 0 0];

consessstruct{1}.tcon.sessrep = 'replsc';

consessstruct{2}.tcon.name='PPI noncanonical';

consessstruct{2}.tcon.weights=[0 0 0 0 1 0 0];

consessstruct{2}.tcon.sessrep = 'replsc';

construct = struct( 'spmmat', matfile_path,...

'consess', {consessstruct});

jobs{1}.stats{1}.con = construct;

spm_jobman('run',jobs);

clear jobs;

function reg_WM_CSF=create_WM_CSF_reg(datacwd,BOLD_filename,data_filter,mask_path,mask_name,TR)

template_path=fullfile(spm('dir'),'tpm','TPM.nii');

tmp_WM_id=2;

tmp_CSF_id=3;

data_name=fullfile(datacwd,[data_filter,BOLD_filename]);

data_vol=spm_vol(data_name);

[data,~]=spm_read_vols(data_vol);

tmp_vol=spm_vol(template_path);

[tmp_data,~]=spm_read_vols(tmp_vol);

mask_vol=spm_vol(fullfile(mask_path,mask_name));

[mask_data,~]=spm_read_vols(mask_vol);

reg_WM=get_reg_vals(tmp_WM_id,data_vol,template_path,data,mask_data,mask_path,mask_name,TR);

reg_CSF=get_reg_vals(tmp_CSF_id,data_vol,template_path,data,mask_data,mask_path,mask_name,TR);

reg_WM_CSF=[reg_WM,reg_CSF];

function reg=get_reg_vals(id,data_vol,template_path,data,mask_data,mask_path,mask_name,TR)

nscans=size(data,4);

threshold=0.9;

Q_mask=find(mask_data == 1);

P{1} = fullfile(mask_path,mask_name);

P{2} = template_path;

Pi=strvcat(P{1}, P{2});

[Qo,template_Vo] = spm_reslice_MG(Pi);

[tmp_Y,tmp_XYZ] = spm_read_vols(template_Vo);

tmp_Y=squeeze(tmp_Y(:,:,:,id));

Q1 = find(tmp_Y > threshold);

Q3 = Q1;

Q3(~ismember(Q1,Q_mask))=[];

tmp_mm=tmp_XYZ(:,Q3);

mx1=[tmp_mm; ones(1,size(tmp_mm,2))];

tmp_vx=data_vol(1).mat\mx1;

tmp_vx=tmp_vx(1:3,:);

tmp_BOLD = spm_get_data(data_vol,tmp_vx);

y = spm_detrend(tmp_BOLD,1);

K.RT = TR;

K.row = 1:nscans;

K.HParam = 128;

K = spm_filter(K);

y = spm_filter(K,y);

ex=[];

for in=1:size(y,2)

if sum(isnan(y(:,in)))>0

ex(end+1)=in;

end

end

y(:,ex)=[];

[reg,~]=PCA_MG(y);

reg=reg(:,1);

function excluded_num=prepare_number_MG(excluded)

N=size(excluded,2);

excluded_num=[];

new_num=[];

for i=1:N

tmp=excluded(1,i);

if ~strcmp(tmp,' ')

new_num=[new_num,tmp];

else

excluded_num=[excluded_num,str2double(new_num)];

new_num=[];

end

end

excluded_num=[excluded_num,str2double(new_num)];

excluded_num(isnan(excluded_num))=[];

function GML_reg=create_GLM_reg(excluded_scans,nscans)

% Function creates regressors suitable for regressing out scans affected

% with severe motion in GLM analysis. Beware, if there are too many

% regressors; it affects degrees of freedom.

n_excluded=numel(excluded_scans);

GML_reg=zeros(nscans,n_excluded);

for i=1:n_excluded

GML_reg(excluded_scans(i),i)=1;

end

function [components,var]=PCA_MG(Y)

% Function performs PCA analysis using svd. Based on SPM toolbox.

% Input: Y - n signals for PCA decomposition - size Y:(signal length, n)

% Output: components - sorted N components - size components:(signal length, n)

% var - explained variability [%] for every component - size components:(1, n)

% ------------------------------------------------------------------------

% Based on svd used in SPM toolbox

% Version 1.01 - use spm_detrend instead of ica_tb_detrend

% ------------------------------------------------------------------------

% Written by Martin Gajdos

% ------------------------------------------------------------------------

[m,n] = size(Y);

Y = spm_detrend(Y,1);

if m > n

[v s v] = svd(Y'*Y);

s = diag(s); % s - eigenvalues

s_perc=100*(s/sum(s));

v = v(:,1:numel(s_perc));

u = NaN(m,numel(s_perc));

for i=1:numel(s_perc)

u(:,i) = Y*v(:,i)/sqrt(s(i));

end

else

[u s u] = svd(Y*Y');

s = diag(s); % s - eigenvalues

s_perc=100*(s/sum(s));

u = u(:,1:numel(s_perc));

v = NaN(n,numel(s_perc));

for i=1:numel(s_perc)

v(:,i) = Y'*u(:,i)/sqrt(s(i));

end

end

d = sign(sum(v));

u = u.*repmat(d,m,1);

components=NaN(m,numel(s_perc));

var=s_perc; % Explained variability [%]

for i=1:numel(s_perc)

components(:,i) = u(:,i)*sqrt(s(i)/n); % First eigenvariate {scaled - c.f. mean response}

end

function A = stand(A,flag)

% Function from SPM8

if ~exist('flag','var')

flag = 1;

end

switch flag

case 0 % remove mean

A = A - repmat(mean(A),size(A,1),1);

case 1 % remove mean and normalize to std=1

A = A - repmat(mean(A),size(A,1),1);

A = A ./ repmat(std(A),size(A,1),1);

case 2 % normalize to std=1

A = A ./ repmat(std(A),size(A,1),1);

case 3 % remove mean, normalize to std=1, add mean

mA = mean(A);

A = A - repmat(mA,size(A,1),1);

A = A ./ repmat(std(A),size(A,1),1);

A = A + repmat(mA,size(A,1),1);

end

Code S5: *F_03A_Second_level_One_sample_t_test_batch.m* Code used second level one sample t-test.

function F_03A_Second_level_One_sample_t_test_batch

pathdef=load('paths.mat');

cwd = pathdef.cwd;

VOI_input=load('vois.mat');

subfolders=VOI_input.subfolders;

nVOIs=size(subfolders,1);

stat_files={

'con_0001.nii' 'PPI canonical'

'con_0002.nii' 'PPI noncanonical'

};

nstat_files=size(stat_files,1);

ntests=nVOIs*nstat_files;

comparisons={

'01 All vs zero' [1 2]

'03 PD vs zero' [2]

'04 HC vs zero' [1]

};

ncomparisons=size(comparisons,1);

input='subjects.mat';

foldname_all=input.subject_data;

foldname=foldname_all(:,1);

group_id=cell2mat(foldname_all(:,2));

age_col=6;

cov.age=cell2mat(foldname_all(:,age_col));

LED_col=9;

cov.LED=cell2mat(foldname_all(:,LED_col));

nsub=size(foldname,1);

for comp_id=1:ncomparisons

if numel(comparisons{comp_id,2})==1

group_ind=find(group_id==comparisons{comp_id,2});

else

group_ind=find(ones(nsub,1));

end

for i=1:nVOIs

for j=1:nstat_files

output_dir=fullfile(cwd,'PPI_group_stats',subfolders{i},stat_files{j,2},comparisons{comp_id,1});

scans=cell(nsub,1);

for sub=1:nsub

q = spm_select('fplist',fullfile(cwd,'PPI_group_stats',subfolders{i},stat_files{j,2}),['^con.*\_',foldname{sub},'.nii$']);

scans{sub,1} = [q,',1'];

end

perform_t_test(scans,output_dir,i,ntests,group_ind,cov)

end

end

end

function perform_t_test(scans,output_dir,i,ntests,group_ind,cov)

%% Specify second level

scans=scans(group_ind,:);

cov.age=cov.age(group_ind,:);

cov.LED=cov.LED(group_ind,:);

matlabbatch{1}.spm.stats.factorial_design.dir = {output_dir};

matlabbatch{1}.spm.stats.factorial_design.des.t1.scans = [scans];

matlabbatch{1}.spm.stats.factorial_design.cov(1).c = cov.age;

matlabbatch{1}.spm.stats.factorial_design.cov(1).cname = 'Age';

matlabbatch{1}.spm.stats.factorial_design.cov(1).iCFI = 1;

matlabbatch{1}.spm.stats.factorial_design.cov(1).iCC = 1;

matlabbatch{1}.spm.stats.factorial_design.cov(2).c = cov.LED;

matlabbatch{1}.spm.stats.factorial_design.cov(2).cname = 'LED';

matlabbatch{1}.spm.stats.factorial_design.cov(2).iCFI = 1;

matlabbatch{1}.spm.stats.factorial_design.cov(2).iCC = 1;

matlabbatch{1}.spm.stats.factorial_design.multi_cov = struct('files', {}, 'iCFI', {}, 'iCC', {});

matlabbatch{1}.spm.stats.factorial_design.masking.tm.tm_none = 1;

matlabbatch{1}.spm.stats.factorial_design.masking.im = 1;

matlabbatch{1}.spm.stats.factorial_design.masking.em = {''};

matlabbatch{1}.spm.stats.factorial_design.globalc.g_omit = 1;

matlabbatch{1}.spm.stats.factorial_design.globalm.gmsca.gmsca_no = 1;

matlabbatch{1}.spm.stats.factorial_design.globalm.glonorm = 1;

matfile_path=fullfile(output_dir,'SPM.mat');

spm_unlink(matfile_path)

spm_jobman('initcfg')

spm_jobman('run',matlabbatch);

clear matlabbatch;

%% Estimate

matlabbatch{1}.spm.stats.fmri_est.spmmat = {matfile_path};

matlabbatch{1}.spm.stats.fmri_est.write_residuals = 0;

matlabbatch{1}.spm.stats.fmri_est.method.Classical = 1;

spm_jobman('run',matlabbatch);

clear matlabbatch;

%% Contrast manager

matlabbatch{1}.spm.stats.con.spmmat = {matfile_path};

matlabbatch{1}.spm.stats.con.consess{1}.tcon.name = 'All vs baseline';

matlabbatch{1}.spm.stats.con.consess{1}.tcon.weights = [1];

matlabbatch{1}.spm.stats.con.consess{1}.tcon.sessrep = 'none';

matlabbatch{1}.spm.stats.con.delete = 1;

spm_jobman('run',matlabbatch);

clear matlabbatch;

disp([' '])

disp(['Group statistics ',num2str(i),'/', num2str(ntests),' completed'])

disp([' '])

Code S6: *function F_03B_Second_level_Two_sample_t_test.m* Code used for second level two sample t-tests.

function F_03B_Second_level_Two_sample_t_test

pathdef=load('paths.mat');

cwd = pathdef.cwd;

VOI_input=load('vois.mat');

subfolders=VOI_input.subfolders;

nVOIs=size(subfolders,1);

stat_files={

'con_0001.nii' 'PPI canonical'

'con_0002.nii' 'PPI noncanonical'

};

nstat_files=size(stat_files,1);

comparisons={'02 PD vs HC';

};

input='subjects.mat';

foldname_all=input.subject_data;

foldname=foldname_all(:,1);

group_reg_cell= foldname_all(:,1:2);

age_col=6;

cov.age=cell2mat(foldname_all(:,age_col));

LED_col=9;

cov.LED=cell2mat(foldname_all(:,LED_col));

for i=1:nVOIs

for j=1:nstat_files

second_level_stat_groups(cwd,subfolders{i},stat_files{j,2},comparisons{1,1},group_reg_cell,foldname,cov);

end

end

disp([' '])

disp(['Group statistics completed'])

disp([' '])

function second_level_stat_groups(cwd,subfolder,contrast_folder,comparison,group_reg_cell,foldname,cov)

outputfolder=fullfile(cwd,'PPI_group_stats',subfolder,contrast_folder,comparison);

name_ID_column=1;

group_ID_column=2;

nsub=size(foldname,1);

G1.scans = [];

G1.age = [];

G1.LED = [];

G2.scans = [];

G2.age = [];

G2.LED = [];

for sub=1:nsub

names_xls{sub,1}=group_reg_cell{sub,name_ID_column};

end

for sub=1:nsub

IndexC = strfind(names_xls,foldname{sub});

new_ind(sub) = find(not(cellfun('isempty', IndexC)));

end

for sub=1:nsub

xls_id=new_ind(sub);

if group_reg_cell{xls_id,group_ID_column}==2

q = spm_select('fplist',fullfile(cwd,'PPI_group_stats',subfolder,contrast_folder),['^con.*\_',foldname{sub},'.nii$']);

if isempty(q)

continue

end

G1.scans{end+1,1} = [q,',1'];

G1.age(end+1,1)=cov.age(xls_id,1);

G1.LED(end+1,1)=cov.LED(xls_id,1);

elseif group_reg_cell{xls_id,group_ID_column}==1

q = spm_select('fplist',fullfile(cwd,'PPI_group_stats',subfolder,contrast_folder),['^con.*\_',foldname{sub},'.nii$']);

if isempty(q)

continue

end

G2.scans{end+1,1} = [q,',1'];

G2.age(end+1,1)=cov.age(xls_id,1);

G2.LED(end+1,1)=cov.LED(xls_id,1);

end

end

G.age=[G1.age;G2.age];

G.LED=[G1.LED;G2.LED];

%% Specify second level

matlabbatch{1}.spm.stats.factorial_design.dir = {outputfolder};

matlabbatch{1}.spm.stats.factorial_design.des.t2.scans1 = G1.scans;

matlabbatch{1}.spm.stats.factorial_design.des.t2.scans2 = G2.scans;

matlabbatch{1}.spm.stats.factorial_design.des.t2.dept = 0;

matlabbatch{1}.spm.stats.factorial_design.des.t2.variance = 1;

matlabbatch{1}.spm.stats.factorial_design.des.t2.gmsca = 0;

matlabbatch{1}.spm.stats.factorial_design.des.t2.ancova = 0;

matlabbatch{1}.spm.stats.factorial_design.cov(1).c = G.age;

matlabbatch{1}.spm.stats.factorial_design.cov(1).cname = 'Age';

matlabbatch{1}.spm.stats.factorial_design.cov(1).iCFI = 1;

matlabbatch{1}.spm.stats.factorial_design.cov(1).iCC = 1;

matlabbatch{1}.spm.stats.factorial_design.cov(2).c = G.LED;

matlabbatch{1}.spm.stats.factorial_design.cov(2).cname = 'LED';

matlabbatch{1}.spm.stats.factorial_design.cov(2).iCFI = 1;

matlabbatch{1}.spm.stats.factorial_design.cov(2).iCC = 1;

matlabbatch{1}.spm.stats.factorial_design.multi_cov = struct('files', {}, 'iCFI', {}, 'iCC', {});

matlabbatch{1}.spm.stats.factorial_design.masking.tm.tm_none = 1;

matlabbatch{1}.spm.stats.factorial_design.masking.im = 1;

matlabbatch{1}.spm.stats.factorial_design.masking.em = {''};

matlabbatch{1}.spm.stats.factorial_design.globalc.g_omit = 1;

matlabbatch{1}.spm.stats.factorial_design.globalm.gmsca.gmsca_no = 1;

matlabbatch{1}.spm.stats.factorial_design.globalm.glonorm = 1;

spm_jobman('initcfg')

spm_jobman('run',matlabbatch);

clear matlabbatch;

matfile_path=fullfile(outputfolder,'SPM.mat');

%% Estimate

matlabbatch{1}.spm.stats.fmri_est.spmmat = {matfile_path};

matlabbatch{1}.spm.stats.fmri_est.write_residuals = 0;

matlabbatch{1}.spm.stats.fmri_est.method.Classical = 1;

spm_jobman('run',matlabbatch);

clear matlabbatch;

%% Conrast manager

matlabbatch{1}.spm.stats.con.spmmat = {matfile_path};

matlabbatch{1}.spm.stats.con.consess{1}.tcon.name = comparison;

matlabbatch{1}.spm.stats.con.consess{1}.tcon.weights = [1 -1];

matlabbatch{1}.spm.stats.con.consess{1}.tcon.sessrep = 'none';

matlabbatch{1}.spm.stats.con.delete = 1;

spm_jobman('run',matlabbatch);

clear matlabbatch;

Code S7: *F_03C_extract_PPI_age_reg.m* Code used for extraction of PPI values.

function F_03C_extract_PPI_age_reg

contrast_cell={

'PPI canonical' 1

'PPI noncanonical' 2

};

group_desc={1 'HC'

2 'MCI-LB'};

N_contrasts=size(contrast_cell,1);

VOI_input=load('vois.mat');

ROI_couples=VOI_input.ROI_couples;

ROI_header=VOI_input.ROI_header;

N=size(ROI_couples,1);

input='subjects.mat';

foldname=input.subject_data;

nsub=size(foldname,1);

study_data.foldname=foldname;

age_col=6;

study_data.save_figures=1; %

pathdef=load('paths.mat');

study_data.res_path=pathdef.res_path;

study_data.cwd =pathdef.cwd;

study_data.source_path = pathdef.source_path;

study_data.VOI_path = pathdef.VOI_path;

save_xlsx=1;

save_mat=1;

group_vect=foldname(:,2);

age_vect=cell2mat(foldname(:,age_col));

regress_age=1;

PPI_data_all=cell(N_contrasts,1);

for i=1:N_contrasts

tmp_contrast=contrast_cell(i,:);

PPI_data=cell(nsub,N);

PPI_stat=cell(N,4);

for j=1:N

PPI_data(:,j)=get_PPI(foldname,study_data,ROI_couples(j,:),tmp_contrast);

if regress_age==1

PPI_data(:,j)=GLM_filt(PPI_data(:,j),age_vect);

end

[PPI_stat(j,:),stat_header]=compute_stat(PPI_data(:,j),foldname,group_desc);

disp(['Completed: Contrast ',tmp_contrast{1,1},' Connection ',num2str(j),'/',num2str(N)])

end

PPI_table=[[{'Subject ID' 'Group (1=HC, 2=PD)'},ROI_header'];[foldname(:,[1:2]),PPI_data]];

PPI_stat_table=[[{'Connection'},stat_header];[ROI_header,PPI_stat]];

if save_xlsx==1

res_name='PPI_analysis.xlsx';

writecell(PPI_table,res_name,'Sheet',[tmp_contrast{1,1},' values'])

writecell(PPI_stat_table,res_name,'Sheet',[tmp_contrast{1,1},' stat'])

end

PPI_data_all{i,1}.PPI_table=PPI_table;

PPI_data_all{i,1}.PPI_stat_table=PPI_stat_table;

end

if save_mat==1

res_name='PPI_ROI_analysis_v03_Fedorenko_Saur_age_reg.mat';

save(res_name,'PPI_data_all','contrast_cell','ROI_couples', 'group_desc', 'study_data')

end

function Y_filt=GLM_filt(Y,X)

Y=cell2mat(Y);

nreg=size(X,2);

for i=1:nreg

X(:,i)=X(:,i)-mean(X(:,i));

end

X=[ones(size(X,1),1) X];

b=pinv(X)*Y;

e=Y-X*b;

ee=e+X(:,1)*b(1);

Y_filt=ee;

Y_filt=num2cell(Y_filt);

function PPI_data=get_PPI(foldname,study_data,ROI_couple,contrast_info)

nsub=size(foldname,1);

subjects=foldname(:,1);

PPI_path=study_data.source_path;

cwd=study_data.res_path;

PPI_folder=contrast_info{1,1};

PPI_data=cell(nsub,1);

ROI2_mask=ROI_couple{1,2};

ROI2_mask_fullname=fullfile(study_data.VOI_path,ROI2_mask);

for i=1:nsub

tmp_subject=subjects{i,1};

seed_name=ROI_couple{1,1};

con_name=['con_000',num2str(contrast_info{1,2}),'_',tmp_subject,'.nii'];

con_fullname=fullfile(PPI_path,seed_name,PPI_folder,con_name);

tmp_PPI_value=extract_con_data(con_fullname,ROI2_mask_fullname,cwd);

PPI_data{i,1}=nanmean(tmp_PPI_value);

end

function PPI_values=extract_con_data(con_fullname,ROI2_mask_fullname,cwd)

V=spm_vol(con_fullname);

[Y,XYZ] = spm_read_vols(V);

flags.prefix=fullfile(cwd,'tmp');

P{1} = [con_fullname,',1'];

P{2} = [ROI2_mask_fullname,',1'];

Pi=strvcat(P{1}, P{2});

spm_unlink([flags.prefix,'.nii'])

[Qo,VOI_vol] = spm_reslice_MG(Pi,flags);

[VOI,~] = spm_read_vols(VOI_vol);

ind=find(VOI==1);

maskXYZ = XYZ(:,ind);

mx=[maskXYZ; ones(1,size(maskXYZ,2))];

mxv=V(1).mat\mx;

mxv=mxv(1:3,:);

PPI_values = spm_get_data(V,mxv);

spm_unlink([flags.prefix,'.nii'])

function [PPI_stat,stat_header]=compute_stat(PPI_data,foldname,group_desc)

group_vect=cell2mat(foldname(:,2));

G1_ind=find(group_vect==group_desc{1,1});

G2_ind=find(group_vect==group_desc{2,1});

G1=cell2mat(PPI_data(G1_ind,1));

G2=cell2mat(PPI_data(G2_ind,1));

PPI_stat{1,1}=get_descrip_vals2(G1);

PPI_stat{2,1}=get_descrip_vals2(G2);

[~,PPI_stat{3,1}]=ttest2(G1,G2);

PPI_stat{4,1}=ranksum(G1,G2);

stat_header={'HC mean ± std (median)', 'MCI-LB mean ± std (median)','p value t-test', 'p value ranksum'};

function val=get_descrip_vals2(vector)

mean_val=num2str(nanmean(vector),'%.3f');

std_val=num2str(nanstd(vector),'%.3f');

median_val=num2str(nanmedian(vector),'%.3f');

valid_N=sum(~isnan(vector));

val=[mean_val,' ± ',std_val, ' (',median_val,'); Valid N = ', num2str(valid_N)];

function [resliced_name,resliced_vol]=spm_reslice_MG(P,flags)

% Function spm_reslice edited by MG 23.1.2015. Changed type of saving of

% resliced image. All images are resliced to the first image.

%

%__________________________________________________________________________

%

% function spm_reslice(P,flags)

% Rigid body reslicing of images

% FORMAT spm_reslice(P,flags)

%

% P - matrix or cell array of filenames {one string per row}

% All operations are performed relative to the first image.

% ie. Coregistration is to the first image, and resampling

% of images is into the space of the first image.

%

% flags - a structure containing various options. The fields are:

%

% mask - mask output images (true/false) [default: true]

% To avoid artifactual movement-related variance the

% realigned set of images can be internally masked, within

% the set (i.e. if any image has a zero value at a voxel

% than all images have zero values at that voxel). Zero

% values occur when regions 'outside' the image are moved

% 'inside' the image during realignment.

%

% mean - write mean image (true/false) [default: true]

% The average of all the realigned scans is written to

% an image file with 'mean' prefix.

%

% interp - the B-spline interpolation method [default: 1]

% Non-finite values result in Fourier interpolation. Note

% that Fourier interpolation only works for purely rigid

% body transformations. Voxel sizes must all be identical

% and isotropic.

%

% which - values of 0, 1 or 2 are allowed [default: 2]

% 0 - don't create any resliced images.

% Useful if you only want a mean resliced image.

% 1 - don't reslice the first image.

% The first image is not actually moved, so it may

% not be necessary to resample it.

% 2 - reslice all the images.

% If which is a 2-element vector, flags.mean will be set

% to flags.which(2).

%

% wrap - three values of either 0 or 1, representing wrapping in

% each of the dimensions. For fMRI, [1 1 0] would be used.

% For PET, it would be [0 0 0]. [default: [0 0 0]]

%

% prefix - prefix for resliced images [default: 'r']

%

%__________________________________________________________________________

%

% The spatially realigned images are written to the original subdirectory

% with the same (prefixed) filename. They are all aligned with the first.

%

% Inputs:

% A series of images conforming to SPM data format (see 'Data Format'). The

% relative displacement of the images is stored in their header.

%

% Outputs:

% The routine uses information in their headers and writes the realigned

% image files to the same subdirectory with a prefix.

%__________________________________________________________________________

% Copyright (C) 1999-2011 Wellcome Trust Centre for Neuroimaging

% John Ashburner

% $Id: spm_reslice.m 4490 2011-09-14 16:22:27Z guillaume $

%__________________________________________________________________________

%

% The headers of the images contain a 4x4 affine transformation matrix 'M',

% usually affected by the `realignment' and `coregistration' modules.

% What these matrices contain is a mapping from the voxel coordinates

% (x0,y0,z0) (where the first voxel is at coordinate (1,1,1)), to

% coordinates in millimeters (x1,y1,z1).

%

% x1 = M(1,1)*x0 + M(1,2)*y0 + M(1,3)*z0 + M(1,4)

% y1 = M(2,1)*x0 + M(2,2)*y0 + M(2,3)*z0 + M(2,4)

% z1 = M(3,1)*x0 + M(3,2)*y0 + M(3,3)*z0 + M(3,4)

%

% Assuming that image1 has a transformation matrix M1, and image2 has a

% transformation matrix M2, the mapping from image1 to image2 is: M2\M1

% (ie. from the coordinate system of image1 into millimeters, followed

% by a mapping from millimeters into the space of image2).

%

% Several spatial transformations (realignment, coregistration,

% normalisation) can be combined into a single operation (without the

% necessity of resampling the images several times).

%__________________________________________________________________________

%

% Refs:

%

% Friston KJ, Williams SR, Howard R Frackowiak RSJ and Turner R (1995)

% Movement-related effect in fMRI time-series. Mag. Res. Med. 35:346-355

%

% W. F. Eddy, M. Fitzgerald and D. C. Noll (1996) Improved Image

% Registration by Using Fourier Interpolation. Mag. Res. Med. 36(6):923-931

%

% R. W. Cox and A. Jesmanowicz (1999) Real-Time 3D Image Registration

% for Functional MRI. Mag. Res. Med. 42(6):1014-1018

%__________________________________________________________________________

SVNid = '$Rev: 4490 $';

%-Say hello

%--------------------------------------------------------------------------

SPMid = spm('FnBanner',mfilename,SVNid);

%-Parameters

%--------------------------------------------------------------------------

if ~nargin || isempty(P), P = spm_select([2 Inf],'image'); end

if iscellstr(P), P = char(P); end

if ischar(P), P = spm_vol(P); end

def_flags = spm_get_defaults('realign.write');

% MG 23.1.2015

%--------------------------------------------------------------------------

def_flags.prefix = 'r';

% def_flags.prefix = flags.prefix;

%--------------------------------------------------------------------------

if nargin < 2

flags = def_flags;

else

fnms = fieldnames(def_flags);

for i=1:length(fnms)

if ~isfield(flags,fnms{i})

flags.(fnms{i}) = def_flags.(fnms{i});

end

end

end

if numel(flags.which) == 2

flags.mean = flags.which(2);

flags.which = flags.which(1);

elseif ~isfield(flags,'mean')

flags.mean = 1;

end

% MG 23.1.2015

%--------------------------------------------------------------------------

flags.mask=0;

flags.mean=0;

flags.interp=0;

flags.which=1;

flags.wrap=[1 1 0];

% flags.prefix='tmp';

%--------------------------------------------------------------------------

%-Reslice

%--------------------------------------------------------------------------

reslice_images(P,flags);

% MG 23.1.2015

%--------------------------------------------------------------------------

resliced_name=[flags.prefix,'.nii'];

resliced_vol=spm_vol(resliced_name);

%--------------------------------------------------------------------------

fprintf('%-40s: %30s\n','Completed',spm('time')) %-#

%==========================================================================

%-function reslice_images(P,flags)

%==========================================================================

function reslice_images(P,flags)

% Reslice images volume by volume

% FORMAT reslice_images(P,flags)

% See main function for a description of the input parameters

if ~isfinite(flags.interp), % Use Fourier method

% Check for non-rigid transformations in the matrixes

for i=1:numel(P)

pp = P(1).mat\P(i).mat;

if any(abs(svd(pp(1:3,1:3))-1)>1e-7)

fprintf('\n Zooms or shears appear to be needed');

fprintf('\n (probably due to non-isotropic voxels).');

fprintf('\n These can not yet be done using the');

fprintf('\n Fourier reslicing method. Switching to');

fprintf('\n 7th degree B-spline interpolation instead.\n\n');

flags.interp = 7;

break

end

end

end

if flags.mask || flags.mean

spm_progress_bar('Init',P(1).dim(3),'Computing available voxels','planes completed');

x1 = repmat((1:P(1).dim(1))',1,P(1).dim(2));

x2 = repmat( 1:P(1).dim(2) ,P(1).dim(1),1);

if flags.mean

Count = zeros(P(1).dim(1:3));

Integral = zeros(P(1).dim(1:3));

end

if flags.mask, msk = cell(P(1).dim(3),1); end;

for x3 = 1:P(1).dim(3)

tmp = zeros(P(1).dim(1:2));

for i = 1:numel(P)

tmp = tmp + getmask(inv(P(1).mat\P(i).mat),x1,x2,x3,P(i).dim(1:3),flags.wrap);

end

if flags.mask, msk{x3} = find(tmp ~= numel(P)); end;

if flags.mean, Count(:,:,x3) = tmp; end;

spm_progress_bar('Set',x3);

end

end

nread = numel(P);

if ~flags.mean

if flags.which == 1, nread = nread - 1; end;

if flags.which == 0, nread = 0; end;

end

spm_progress_bar('Init',nread,'Reslicing','volumes completed');

[x1,x2] = ndgrid(1:P(1).dim(1),1:P(1).dim(2));

nread = 0;

d = [flags.interp*[1 1 1]' flags.wrap(:)];

for i = 1:numel(P)

if (i>1 && flags.which==1) || flags.which==2

write_vol = 1;

else

write_vol = 0;

end

if write_vol || flags.mean

read_vol = 1;

else

read_vol = 0;

end

if read_vol

if ~isfinite(flags.interp)

v = abs(kspace3d(spm_bsplinc(P(i),[0 0 0 ; 0 0 0]'),P(1).mat\P(i).mat));

for x3 = 1:P(1).dim(3)

if flags.mean

Integral(:,:,x3) = ...

Integral(:,:,x3) + ...

nan2zero(v(:,:,x3) .* ...

getmask(inv(P(1).mat\P(i).mat),x1,x2,x3,P(i).dim(1:3),flags.wrap));

end

if flags.mask

tmp = v(:,:,x3); tmp(msk{x3}) = NaN; v(:,:,x3) = tmp;

end

end

else

C = spm_bsplinc(P(i), d);

v = zeros(P(1).dim);

for x3 = 1:P(1).dim(3)

[tmp,y1,y2,y3] = getmask(inv(P(1).mat\P(i).mat),x1,x2,x3,P(i).dim(1:3),flags.wrap);

v(:,:,x3) = spm_bsplins(C, y1,y2,y3, d);

% v(~tmp) = 0;

if flags.mean

Integral(:,:,x3) = Integral(:,:,x3) + nan2zero(v(:,:,x3));

end

if flags.mask

tmp = v(:,:,x3); tmp(msk{x3}) = NaN; v(:,:,x3) = tmp;

end

end

end

if write_vol

VO = P(i);

try

VO.fname = spm_file([flags.prefix,'.nii']); % Změna - definice místa uložení

catch

VO.fname = spm_file(P(i).fname, 'prefix',flags.prefix);

end

VO.dim = P(1).dim(1:3);

VO.dt = P(i).dt;

% VO.dt = [spm_type('int16') P(i).dt(2)];

VO.pinfo = P(i).pinfo;

VO.mat = P(1).mat;

VO.descrip = 'spm - realigned';

VO = spm_write_vol(VO,v);

end

nread = nread + 1;

end

spm_progress_bar('Set',nread);

end

if flags.mean

% Write integral image (16 bit signed)

%----------------------------------------------------------------------

Integral = Integral./Count;

PO = P(1);

PO = rmfield(PO,'pinfo');

PO.fname = spm_file(P(1).fname, 'prefix','mean');

PO.pinfo = [max(max(max(Integral)))/32767 0 0]';

PO.descrip = 'spm - mean image';

PO.dt = [spm_type('int16') spm_platform('bigend')];

spm_write_vol(PO,Integral);

end

spm_progress_bar('Clear');

%==========================================================================

%-function v = kspace3d(v,M)

%==========================================================================

function v = kspace3d(v,M)

% 3D rigid body transformation performed as shears in 1D Fourier space

% FORMAT v = kspace3d(v,M)

% v - image stored as a 3D array

% M - rigid body transformation matrix

%

% v - transformed image

%

% References:

% R. W. Cox and A. Jesmanowicz (1999)

% Real-Time 3D Image Registration for Functional MRI

% Magnetic Resonance in Medicine 42(6):1014-1018

%

% W. F. Eddy, M. Fitzgerald and D. C. Noll (1996)

% Improved Image Registration by Using Fourier Interpolation

% Magnetic Resonance in Medicine 36(6):923-931

[S0,S1,S2,S3] = shear_decomp(M);

d = [size(v) 1 1 1];

g = 2.^ceil(log2(d));

if any(g~=d)

tmp = v;

v = zeros(g);

v(1:d(1),1:d(2),1:d(3)) = tmp;

clear tmp;

end

% XY-shear

tmp1 = -sqrt(-1)*2*pi*([0:((g(3)-1)/2) 0 (-g(3)/2+1):-1])/g(3);

for j=1:g(2)

t = reshape( exp((j*S3(3,2) + S3(3,1)*(1:g(1)) + S3(3,4)).'*tmp1) ,[g(1) 1 g(3)]);

v(:,j,:) = real(ifft(fft(v(:,j,:),[],3).*t,[],3));

end

% XZ-shear

tmp1 = -sqrt(-1)*2*pi*([0:((g(2)-1)/2) 0 (-g(2)/2+1):-1])/g(2);

for k=1:g(3)

t = exp( (k*S2(2,3) + S2(2,1)*(1:g(1)) + S2(2,4)).'*tmp1);

v(:,:,k) = real(ifft(fft(v(:,:,k),[],2).*t,[],2));

end

% YZ-shear

tmp1 = -sqrt(-1)*2*pi*([0:((g(1)-1)/2) 0 (-g(1)/2+1):-1])/g(1);

for k=1:g(3)

t = exp( tmp1.'*(k*S1(1,3) + S1(1,2)*(1:g(2)) + S1(1,4)));

v(:,:,k) = real(ifft(fft(v(:,:,k),[],1).*t,[],1));

end

% XY-shear

tmp1 = -sqrt(-1)*2*pi*([0:((g(3)-1)/2) 0 (-g(3)/2+1):-1])/g(3);

for j=1:g(2)

t = reshape( exp( (j*S0(3,2) + S0(3,1)*(1:g(1)) + S0(3,4)).'*tmp1) ,[g(1) 1 g(3)]);

v(:,j,:) = real(ifft(fft(v(:,j,:),[],3).*t,[],3));

end

if any(g~=d), v = v(1:d(1),1:d(2),1:d(3)); end

%==========================================================================

%-function [S0,S1,S2,S3] = shear_decomp(A)

%==========================================================================

function [S0,S1,S2,S3] = shear_decomp(A)

% Decompose rotation and translation matrix A into shears S0, S1, S2 and

% S3, such that A = S0*S1*S2*S3. The original procedure is documented in:

% R. W. Cox and A. Jesmanowicz (1999)

% Real-Time 3D Image Registration for Functional MRI

% Magnetic Resonance in Medicine 42(6):1014-1018

A0 = A(1:3,1:3);

if any(abs(svd(A0)-1)>1e-7), error('Can''t decompose matrix'); end

t = A0(2,3); if t==0, t=eps; end

a0 = pinv(A0([1 2],[2 3])')*[(A0(3,2)-(A0(2,2)-1)/t) (A0(3,3)-1)]';

S0 = [1 0 0; 0 1 0; a0(1) a0(2) 1];

A1 = S0\A0; a1 = pinv(A1([2 3],[2 3])')*A1(1,[2 3])'; S1 = [1 a1(1) a1(2); 0 1 0; 0 0 1];

A2 = S1\A1; a2 = pinv(A2([1 3],[1 3])')*A2(2,[1 3])'; S2 = [1 0 0; a2(1) 1 a2(2); 0 0 1];

A3 = S2\A2; a3 = pinv(A3([1 2],[1 2])')*A3(3,[1 2])'; S3 = [1 0 0; 0 1 0; a3(1) a3(2) 1];

s3 = A(3,4)-a0(1)*A(1,4)-a0(2)*A(2,4);

s1 = A(1,4)-a1(1)*A(2,4);

s2 = A(2,4);

S0 = [[S0 [0 0 s3]'];[0 0 0 1]];

S1 = [[S1 [s1 0 0]'];[0 0 0 1]];

S2 = [[S2 [0 s2 0]'];[0 0 0 1]];

S3 = [[S3 [0 0 0]'];[0 0 0 1]];

%==========================================================================

%-function [Mask,y1,y2,y3] = getmask(M,x1,x2,x3,dim,wrp)

%==========================================================================

function [Mask,y1,y2,y3] = getmask(M,x1,x2,x3,dim,wrp)

tiny = 5e-2; % From spm_vol_utils.c

y1 = M(1,1)*x1+M(1,2)*x2+(M(1,3)*x3+M(1,4));

y2 = M(2,1)*x1+M(2,2)*x2+(M(2,3)*x3+M(2,4));

y3 = M(3,1)*x1+M(3,2)*x2+(M(3,3)*x3+M(3,4));

Mask = true(size(y1));

if ~wrp(1), Mask = Mask & (y1 >= (1-tiny) & y1 <= (dim(1)+tiny)); end

if ~wrp(2), Mask = Mask & (y2 >= (1-tiny) & y2 <= (dim(2)+tiny)); end

if ~wrp(3), Mask = Mask & (y3 >= (1-tiny) & y3 <= (dim(3)+tiny)); end

%==========================================================================

%-function vo = nan2zero(vi)

%==========================================================================

function vo = nan2zero(vi)

vo = vi;

vo(~isfinite(vo)) = 0;

Code S8: *F_03CA_correlations_and_plot_values.m* Code used for correlations and visualization.

function F_03CA_correlations_and_plot_values

PPI_input_name='PPI_analysis_v01.mat';

PPI_input=load(PPI_input_name);

behav_data_name='behav.mat';

neuropsy_data_name='neuropsy.mat';

behav_input=load(behav_data_name);

neuropsy_input=load(neuropsy_data_name);

pathdef=load('paths.mat');

cwd = pathdef.cwd;

fig_path=fullfile(cwd,'Corr_Figures');

plot_figures=1;

fig_opt.prepare_fig=plot_figures;

fig_opt.save_figures=0;

fig_opt.plot_normality=0;

fig_opt.path=fig_path;

fig_opt.use_not_normalized_data_for_plot=1;

close all

contrast_id=1;

group_desc=PPI_input.group_desc;

contrast_name=PPI_input.contrast_cell{contrast_id,1};

PPI_table=PPI_input.PPI_data_all{contrast_id,1}.PPI_table;

group_vect=cell2mat(PPI_table(2:end,2));

PPI_data=cell2mat(PPI_table(2:end,3:end));

PPI_data_header=PPI_table(1,3:end);

N_PPI=size(PPI_data,2);

PPI_table_data=PPI_table(2:end,:);

PPI_table_header=PPI_table(1,:);

N_PPI_table_data=size(PPI_table_data,2);

behav_data=behav_input.subjects_sum_tab_data;

behav_header=behav_input.subjects_sum_tab_header;

psy_data=neuropsy_input.neuropsy_data(2:end,2:end);

psy_header=neuropsy_input.neuropsy_data(1,2:end);

psy_data=psy_data(:,[2,1,3:end]);

psy_header=psy_header(:,[2,1,3:end]);

psy_header=transform_header_to_cell_char(psy_header);

psy_subject_col=1;

psy_other_col=2:size(psy_data,2);

psy_data=add_sub_prefix(psy_data,psy_subject_col);

psy_data=preplace_empty_cells_wit_NaN(psy_data,psy_other_col);

tmp1_all_table=merge_tables_MG(PPI_table_data,behav_data);

tmp1_all_table2=[tmp1_all_table(:,1:N_PPI_table_data),tmp1_all_table(:,N_PPI_table_data+2:end)];

tmp1_all_table2_header=[PPI_table_header,behav_header(1,2:end)];

N_tmp1_table=size(tmp1_all_table2,2);

tmp2_all_table=merge_tables_MG(tmp1_all_table2,psy_data);

tmp2_all_table2=[tmp2_all_table(:,1:N_tmp1_table),tmp2_all_table(:,N_tmp1_table+2:end)];

tmp2_all_table2_header=[tmp1_all_table2_header,psy_header(1,2:end)];

all_data=tmp2_all_table2;

all_header=tmp2_all_table2_header;

G1_id=group_desc{1,1};

G2_id=group_desc{2,1};

G1_ind=find(group_vect==G1_id);

G2_ind=find(group_vect==G2_id);

HC_data=all_data(G1_ind,:);

MCI_LB_data=all_data(G2_ind,:);

prepare_boxplots=1;

if prepare_boxplots==1

fig_id=1;

for i=1:N_PPI

tmp_PPI_data=PPI_data(:,i);

box_data=prepare_data_groups(tmp_PPI_data,group_vect,group_desc);

box_desc=[contrast_name,' ', PPI_data_header{1,i}];

prepare_boxplots_fcn(box_data,box_desc,cwd,fig_opt,group_desc,fig_id,N_PPI,i)

end

end

PPI_ind=[3:N_PPI_table_data];

confound={'years'};

psych_behav=behav_input.psych_behav;

psychbehav_ind=find_indices_in_header(all_header,psych_behav);

confound_ind=find_indices_in_header(all_header,confound);

res_tab2=prepare_corr(HC_data,MCI_LB_data,PPI_ind,psychbehav_ind,confound_ind,all_header,fig_opt,contrast_name);

psych_ind1=11;

psych_behav2=behav_input.psych_behav2;

psychbehav_ind2=find_indices_in_header(all_header,psych_behav2);

res_tab3=prepare_corr2(HC_data,MCI_LB_data,psych_ind1,psychbehav_ind2,confound_ind,all_header,fig_opt,contrast_name);

psych_behav3=behav_input.psych_behav3;

psychbehav_ind3=find_indices_in_header(all_header,psych_behav3);

res_tab4=prepare_corr(HC_data,MCI_LB_data,PPI_ind,psychbehav_ind3,confound_ind,all_header,fig_opt,contrast_name);

function new_psy_data=preplace_empty_cells_wit_NaN(psy_data,col)

Ncol=numel(col);

Nrow=size(psy_data,1);

new_psy_data=psy_data;

index=0;

for i=1:Ncol

for j=1:Nrow

tmp=psy_data{j,col(i)};

tmp2=convertStringsToChars(tmp);

if isempty(tmp2)

new_psy_data{j,col(i)}=NaN;

index=index+1;

end

end

end

function psychbehav_ind=find_indices_in_header(all_header,psych_behav)

N=numel(psych_behav);

psychbehav_ind=NaN(1,N);

for i=1:N

Index=find_string_in_cell_MG(psych_behav{i},all_header);

psychbehav_ind(1,i)=Index;

end

function box_data=prepare_data_groups(PPI_data,group_vect,group_desc)

G1_id=group_desc{1,1};

G2_id=group_desc{2,1};

G1_ind=find(group_vect==G1_id);

G2_ind=find(group_vect==G2_id);

box_data{1,1}=PPI_data(G1_ind,1);

box_data{1,2}=PPI_data(G2_ind,1);

function new_psy_data=add_sub_prefix(psy_data,psy_subject_col)

N=size(psy_data,1);

new_psy_data=psy_data;

for i=1:N

tmp=convertStringsToChars(psy_data{i,psy_subject_col});

if numel(tmp)>=5

new_psy_data{i,psy_subject_col}=['sub-',tmp(1:5)];

else

new_psy_data{i,psy_subject_col}=['sub-',tmp];

end

end

function new_psy_header=transform_header_to_cell_char(psy_header)

N=size(psy_header,2);

new_psy_header=cell(1,N);

for i=1:N

tmp=convertStringsToChars(psy_header{1,i});

new_psy_header{1,i}=tmp;

end

function prepare_boxplots_fcn(box_data,box_desc,cwd,fig_opt,group_desc,fig_id,N_subplots,subplot_index)

fig=figure(fig_id);

title_str=box_desc;

title_str(title_str=='_')=' ';

boxplot_data=prepare_box_data(box_data);

subplot(1,N_subplots,subplot_index)

boxplot(boxplot_data,'Labels',{group_desc{1,2},group_desc{2,2}});

hold on

title(title_str)

grid on

ylim([-0.4,0.7])

ylabel('[arb. unit]')

set(gcf, 'Position', get(0, 'Screensize')*(3/4))

if fig_opt.save_figures==1

cd(fig_opt.path)

tmp_name=['Fig_',num2str(fig_id),'_',box_desc,'.png'];

fprintf(['Saving figure: ',tmp_name,'\n\n'])

set(fig,'Position', get(0, 'Screensize'))

set(fig,'color','w');

F = getframe(fig);

imwrite(F.cdata, tmp_name, 'png')

end

function boxplot_data=prepare_box_data(box_data)

% Function prepares data for boxplot when input is cell, in each cell are

% different numbers of elements (vectors are expected)

% Output is m x n matrix, where m is maximum number of elements in box_data

% and n is number of cells in box_data

N_data=numel(box_data);

max_N_vect=zeros(N_data,1);

for i=1:N_data

max_N_vect(i,1)=numel(box_data{i});

end

max_N=max(max_N_vect);

boxplot_data=NaN(max_N,N_data);

for i=1:N_data

start=1;

stop=max_N_vect(i,1);

boxplot_data(start:stop,i)=box_data{i};

end

function [res_cell,res_desc_groups]=prepare_corr(HC_data,MCI_LB_data,PPI_ind,psych_ind,confound_ind,all_header,fig_opt,contrast_name)

npar=11;

res_cell=cell(0,npar);

nvar1=numel(PPI_ind);

nvar2=numel(psych_ind);

HC_confound=HC_data(:,confound_ind);

MCI_LB_confound=MCI_LB_data(:,confound_ind);

for i=1:nvar1

for j=1:nvar2

HCdata1=HC_data(:,PPI_ind(i));

HCdata2=HC_data(:,psych_ind(j));

preDLBdata1=MCI_LB_data(:,PPI_ind(i));

preDLBdata2=MCI_LB_data(:,psych_ind(j));

[tmp,res_desc_groups]=perform_corr(HCdata1,HCdata2,preDLBdata1,preDLBdata2,PPI_ind(i),psych_ind(j),fig_opt,all_header,npar,contrast_name,HC_confound,MCI_LB_confound);

res_cell=[res_cell;tmp];

end

end

res_cell=[res_desc_groups;res_cell];

function [res_cell,res_desc_groups]=prepare_corr2(HC_data,preDLB_data,psych_ind1,psych_ind2,confound_ind,all_header,fig_opt,contrast_name)

npar=11;

res_cell=cell(0,npar);

nvar1=numel(psych_ind1);

nvar2=numel(psych_ind2);

HC_confound=HC_data(:,confound_ind);

MCI_LB_confound=preDLB_data(:,confound_ind);

for i=1:nvar1

for j=1:nvar2

HCdata1=HC_data(:,psych_ind1(i));

HCdata2=HC_data(:,psych_ind2(j));

preDLBdata1=preDLB_data(:,psych_ind1(i));

preDLBdata2=preDLB_data(:,psych_ind2(j));

[tmp,res_desc_groups]=perform_corr(HCdata1,HCdata2,preDLBdata1,preDLBdata2,psych_ind1(i),psych_ind2(j),fig_opt,all_header,npar,contrast_name,HC_confound,MCI_LB_confound);

res_cell=[res_cell;tmp];

end

end

res_cell=[res_desc_groups;res_cell];

function [res_cell,res_desc_groups]=perform_corr(HCdata1,HCdata2,MCI_LBdata1,MCI_LBata2,dFC_ind,psych_ind,fig_opt,all_header,npar,contrast_name,HC_confound,PD_confound)

res_cell=cell(1,npar);

HCdata1=replace_empty_by_NaN(HCdata1);

HCdata2=replace_empty_by_NaN(HCdata2);

HCdata1=cell2mat(HCdata1);

HCdata2=cell2mat(HCdata2);

MCI_LBdata1=replace_empty_by_NaN(MCI_LBdata1);

MCI_LBata2=replace_empty_by_NaN(MCI_LBata2);

MCI_LBdata1=cell2mat(MCI_LBdata1);

MCI_LBata2=cell2mat(MCI_LBata2);

HC_confound=cell2mat(HC_confound);

PD_confound=cell2mat(PD_confound);

[HC_vect1,HC_vect2,HC_confound]=remove_invalid_data(HCdata1,HCdata2,HC_confound);

[PD_vect1,PD_vect2,PD_confound]=remove_invalid_data(MCI_LBdata1,MCI_LBata2,PD_confound);

[r_P_PD, p_P_PD]=partialcorr(PD_vect1,PD_vect2,PD_confound,'Type','Pearson');

[r_P_HC, p_P_HC]=partialcorr(HC_vect1,HC_vect2,HC_confound,'Type','Pearson');

[r_S_PD, p_S_PD]=partialcorr(PD_vect1,PD_vect2,PD_confound,'Type','Spearman');

[r_S_HC, p_S_HC]=partialcorr(HC_vect1,HC_vect2,HC_confound,'Type','Spearman');

res_name=[all_header{1,dFC_ind},' - ', all_header{1,psych_ind}];

res_desc_groups={'Description','r Pearson PD','r Spearman PD','p Pearson PD','p Spearman PD', ['Valid N PD ',contrast_name],'r Pearson HC','r Spearman HC','p Pearson HC','p Spearman HC', ['Valid N HC ',contrast_name]};

res_cell{1,1}=res_name;

res_cell{1,2}=r_P_PD;

res_cell{1,3}=r_S_PD;

res_cell{1,4}=p_P_PD;

res_cell{1,5}=p_S_PD;

res_cell{1,6}=[num2str(numel(PD_vect1)),', ',num2str(numel(PD_vect2))];

res_cell{1,7}=r_P_HC;

res_cell{1,8}=r_S_HC;

res_cell{1,9}=p_P_HC;

res_cell{1,10}=p_S_HC;

res_cell{1,11}=[num2str(numel(HC_vect1)),', ',num2str(numel(HC_vect2))];

if fig_opt.prepare_fig==1

r_thres=0.3;

p_thres=0.05;

cond=(abs(r_P_PD)>r_thres)||(abs(r_S_PD)>r_thres)||(abs(r_P_HC)>r_thres)||(abs(r_S_HC)>r_thres)||(p_P_PD<p_thres)||(p_S_PD<p_thres)||(p_P_HC<p_thres)||(p_S_HC<p_thres);

if cond==1

prepare_figures(PD_vect1,PD_vect2,HC_vect1,HC_vect2,res_name,all_header{1,dFC_ind}, all_header{1,psych_ind},fig_opt)

end

end

function Index=find_string_in_cell_MG(str,cell_data)

IndexC = strfind(cell_data,str);

Index = find(not(cellfun('isempty',IndexC)));

function newdata=normalize_data(data)

[N,ndim]=size(data);

newdata=NaN(N,ndim);

for i=1:ndim

tmp=data(:,i);

newdata(:,i)=(tmp-mean(tmp))/std(tmp);

end

function prepare_figures(MCI_LB_vect1,MCI_LB_vect2,HC_vect1,HC_vect2,res_name,xname,yname,fig_opt)

title_str=['Correlation ',res_name];

title_str(title_str=='_')=' ';

xname(xname=='_')=' ';

yname(yname=='_')=' ';

fig=figure;

subplot(111)

hold on

xlabel(xname);

ylabel(yname);

x_min=min([MCI_LB_vect1;HC_vect1]);

x_max=max([MCI_LB_vect1;HC_vect1]);

y_min=min([MCI_LB_vect2;HC_vect2]);

y_max=max([MCI_LB_vect2;HC_vect2]);

range_x=x_max-x_min;

range_y=y_max-y_min;

const=0.1;

gap_x=const*(range_x);

gap_y=const*(range_y);

xlim([x_min-gap_x,x_max+gap_x])

ylim([y_min-gap_y,y_max+gap_y])

MCI_LB_reg_y=prepare_reg(MCI_LB_vect1,MCI_LB_vect2);

HC_reg_y=prepare_reg(HC_vect1,HC_vect2);

plot(MCI_LB_vect1,MCI_LB_reg_y,'r','linewidth',1)

plot(HC_vect1,HC_reg_y,'b','linewidth',1)

plot(MCI_LB_vect1,MCI_LB_vect2,'xr')

plot(HC_vect1,HC_vect2,'xb')

legend({['PD: r_S=',num2str(corr(MCI_LB_vect1,MCI_LB_vect2,'Type','Spearman'),'%.2f')],['HC: r_S=',num2str(corr(HC_vect1,HC_vect2,'Type','Spearman'),'%.2f')]})

grid on

if fig_opt.save_figures==1

cd(fig_opt.path)

tmp_name=[title_str,'_paper.png'];

tmp_name(tmp_name==':')=[];

fprintf(['Saving figure: ',tmp_name,'\n\n'])

set(fig,'Position', round(get(0, 'Screensize')*0.75))

set(fig,'color','w');

F = getframe(fig);

imwrite(F.cdata, tmp_name, 'png')

end

function yCalc2=prepare_reg(X,Y)

X_r = [ones(length(X),1) X];

b = X_r\Y;

yCalc2 = X_r*b;

function data=replace_empty_by_NaN(data)

N=numel(data);

for i=1:N

if isempty(data{i})

data{i}=NaN;

end

end

function [new_vect1,new_vect2,new_confound]=remove_invalid_data(vect1,vect2,confound)

NaN1=find(isnan(vect1));

NaN2=find(isnan(vect2));

NaN_ind=[NaN1;NaN2];

NaN_ind=unique(NaN_ind);

new_vect1=vect1;

new_vect1(NaN_ind)=[];

new_vect2=vect2;

new_vect2(NaN_ind)=[];

new_confound=confound;

new_confound(NaN_ind)=[];

function new_table=merge_tables_MG(primary_table,secondary_table)

merging_col_prim_table=1;

megring_col_secondary_table=1;

n_col1=size(primary_table,2);

n_col2=size(secondary_table,2);

N_row_primary=size(primary_table,1);

reordered_secondary_table=cell(N_row_primary,n_col2);

key_vect_primary=primary_table(:,merging_col_prim_table);

key_vect_secondary=secondary_table(:,megring_col_secondary_table);

for i=1:N_row_primary

tmp_name_primary=key_vect_primary{i,1};

idx = find_MG_fcn(key_vect_secondary, tmp_name_primary);

if ~isempty(idx)

reordered_secondary_table(i,:)=secondary_table(idx,:);

end

end

new_table=[primary_table,reordered_secondary_table];

function idx = find_MG_fcn(key_vect, tmp)

N=numel(key_vect);

tmp2=zeros(N,1);

for i=1:N

tmptmp=key_vect{i};

if strcmp(tmp,tmptmp)

tmp2(i,1)=1;

end

end

idx=find(tmp2);

Code S9: *Prepare_sphere_masks.m* Code used for creating sphere masks.

function Prepare_sphere_masks

VOI_input=load('vois.mat');

coord=VOI_input.coord;

template=VOI_input.template;

N=size(coord,1);

pathdef=load('paths.mat');

outputpath=pathdef.outputpath;

radius = 6;

for i=1:N

prepare_sphere_mask(outputpath,radius,template,coord{i,2},['Sphere_',num2str(radius),'mm_',coord{i,1},'.nii']);

end

function prepare_sphere_mask(outputpath,radius,template,coord_mm,output_name)

tmp_vol=spm_vol([template,',1']);

[tmp_img,tmp_ind] = spm_read_vols(tmp_vol);

[sp_coords]=get_sphere_coords(tmp_vol,tmp_ind,coord_mm,radius);

Y=zeros(tmp_vol.dim(1),tmp_vol.dim(2),tmp_vol.dim(3));

Y(sp_coords.ind)=1;

mask = deal(struct(...

'fname', [],...

'dim', [tmp_vol.dim(1) tmp_vol.dim(2) tmp_vol.dim(3)],...

'dt', [spm_type('int16') 0],...

'mat', tmp_vol(1).mat,...

'pinfo', [1 0 0]',...

'descrip', 'Sphere mask',...

'n', [],...

'private', []));

mask.fname = fullfile(outputpath,output_name);

spm_unlink(mask.fname)

mask = spm_create_vol(mask);

spm_write_vol(mask,Y);

function [sp_coords]=get_sphere_coords(temp_vol,temp_ind,center_pos,r)

sp_coords.mm=[];

sp_coords.ind=[];

for i=1:size(temp_ind,2)

temp_point=temp_ind(:,i);

distance=sqrt((center_pos(1)-temp_point(1)).^2+(center_pos(2)-temp_point(2)).^2+(center_pos(3)-temp_point(3)).^2);

if distance<=r

sp_coords.mm=[sp_coords.mm;temp_point'];

sp_coords.ind=[sp_coords.ind;i];

end

end

sp_coords.vx=NaN(size(sp_coords.mm));

for j=1:size(sp_coords.mm,1)

temp_coo=[sp_coords.mm(j,1) sp_coords.mm(j,2) sp_coords.mm(j,3) 1];

coordsvox=temp_vol.mat\temp_coo';

sp_coords.vx(j,:)=[round(coordsvox(1)) round(coordsvox(2)) round(coordsvox(3))];

end

Code S10: *check_movement.m* Code used for evaluation of movement.

function check_movement

pathdef=load('paths.mat');

parent_folder=pathdef.parent_folder;

res_folder=pathdef.res_folder;

input='subjects.mat';

subject_data=input.subject_data;

n_rp=size(subject_data,1);

crit{1}.FD=0.5;

crit{1}.perc=20;

crit{2}.FD=1.5;

crit{2}.perc=0;

n_crit=numel(crit);

res_list=cell(n_rp,n_crit);

perc_list=cell(n_rp,n_crit);

for i=1:n_rp

tmp=subject_data{i,3}(1:end-4);

tmp(end-5)='2';

tmp_rp=['rp_',tmp,'.txt'];

tmp_rp_full=fullfile(parent_folder,'rp_files',tmp_rp);

for j=1:n_crit

[res_list{i,j},perc_list{i,j}]=check_FD_quality(tmp_rp_full,crit{j});

end

end

res_desc={'perc exceeding crit 1 [%]','perc exceeding crit 2 [%]','is crit 1 OK','is crit 2 OK'};

res_tab=[res_desc;[perc_list,res_list]];

subject_data=[subject_data,perc_list,res_list];

save_results=1;

if save_results==1

save(fullfile(res_folder,'FD_info.mat'),'subject_data','res_tab','crit')

end

function [is_FD_OK,perc]=check_FD_quality(rp_file,crit)

input_is_in_radians=1;

fd_type=1;

mov_par=load(rp_file);

if input_is_in_radians==1

mov_par(:,4:6)=(180/pi).*mov_par(:,4:6);

end

Nactual_scans=size(mov_par,1);

FD=get_FD(mov_par,fd_type);

FD_threshold_ex_scans=crit.FD;

FD_threshold_perc=crit.perc;

tmp_N=numel(FD);

tmp_ind_ex=find(FD>FD_threshold_ex_scans);

if ~isempty(tmp_ind_ex)

n_ex=numel(tmp_ind_ex);

tmp_perc=100*n_ex/tmp_N;

if tmp_perc<=FD_threshold_perc

is_FD_OK=1;

else

is_FD_OK=0;

end

perc=tmp_perc;

else

is_FD_OK=1;

perc=0;

end

function FD=get_FD(mov_par,fd_type)

reg_trans = mov_par(:,1:3);

reg_rot = mov_par(:,4:6);

dif_trans = [0 0 0; (reg_trans(2:end,:)-reg_trans(1:(end-1),:))];

dif_rot = [0 0 0; (reg_rot(2:end,:)-reg_rot(1:(end-1),:))];

% Framewise Displacement

switch fd_type

case 1 % Jonathan Power

abs_trans = abs(dif_trans);

abs_rot = abs(dif_rot);

fd = abs_trans(:,1)+abs_trans(:,2)+abs_trans(:,3)+50*pi/180*(abs_rot(:,1)+abs_rot(:,2)+abs_rot(:,3));

case 2 % Chao-Gan Yan

fd = sqrt((dif_trans(:,1).^2)+(dif_trans(:,2).^2)+(dif_trans(:,3).^2));

case 3 % Koene Van Dijk

fd = sqrt((reg_trans(2:end,1).^2)+(reg_trans(2:end,2).^2)+(reg_trans(2:end,3).^2))-sqrt((reg_trans(1:(end-1),1).^2)+(reg_trans(1:(end-1),2).^2)+(reg_trans(1:(end-1),3).^2));

fd = [0; fd];

end

FD=fd;

Code S11: *Prepare_cluster_masks_p_0_001.m* Code used for preparation of cluster masks.

function empty_VOI=Prepare_cluster_masks_p_0_001

pathdef=load('paths.mat');

data_path=pathdef.data_path;

result_folder=pathdef.result_folder;

VOI_input=load('vois.mat');

VOI_info=VOI_input.VOI_info;

nVOI=size(VOI_info,1);

empty_VOI={};

for i=1:nVOI

nclust=size(VOI_info{i,2},1);

for j=1:nclust

prepare_mask_of_VOI(data_path,result_folder,VOI_info{i,1},VOI_info{i,2});

end

disp(['VOI ',num2str(i),'/',num2str(nVOI),' ready'])

end

function prepare_mask_of_VOI(data_path,result_folder,VOI_name,coord)

model_path=fullfile(data_path);

SPM_model_name=fullfile(model_path,'SPM.mat');

SPM_data=load(SPM_model_name);

spm_T_name=fullfile(model_path,'spmT_0001.nii');

spm_T_vol=spm_vol(spm_T_name);

[spm_T_vals,~]=spm_read_vols(spm_T_vol);

p_threshold=0.001;

eidf=SPM_data.SPM.xCon.eidf;

T_start = strfind(spm_T_vol.descrip,'SPM{T_[')+length('SPM{T_[');

T_end = strfind(spm_T_vol.descrip,']}')-1;

df = [eidf str2double(spm_T_vol.descrip(T_start:T_end))];

t_cut_off_threshold = spm_invTcdf(1-p_threshold,df(2));

spm_T_vals_bin=zeros(size(spm_T_vals));

ind=find(abs(spm_T_vals)>t_cut_off_threshold);

spm_T_vals_bin(ind)=1;

[L,num] = spm_bwlabel(spm_T_vals_bin,18);

coo=[coord 1];

coords_vx=spm_T_vol.mat\coo';

coords_vx=coords_vx(1:3)';

val=L(coords_vx(1),coords_vx(2),coords_vx(3));

cluster_mask=zeros(size(spm_T_vals));

cluster_ind=find(L==val);

cluster_mask(cluster_ind)=1;

clust_struct = deal(struct(...

'fname', [],...

'dim', [spm_T_vol.dim(1) spm_T_vol.dim(2) spm_T_vol.dim(3)],...

'dt', [spm_type('int16') 0],...

'mat', spm_T_vol.mat,...

'pinfo', [1 0 0]',...

'descrip', ['Mask of cluster on ',num2str(coord)],...

'n', [],...

'private', []));

clust_struct.fname = fullfile(result_folder,[VOI_name,'.nii']);

spm_unlink(clust_struct.fname)

clust_struct = spm_create_vol(clust_struct);

spm_write_vol(clust_struct,cluster_mask);

Code S12: *fMRI_preprocessing_batch.m* Code used for preprocessing of fMRI data.

function fMRI_preprocessing_batch

pathdef=load('paths.mat');

path_def.orig_folder=pathdef.orig_folder;

path_def.new_folder=pathdef.new_folder;

path_def.cwd = pathdef.cwd;

path_def.result_subfolder=pathdef.result_subfolder;

path_def.eprime_logs_folder=pathdef.eprime_logs_folder;

path_def.mask_path=pathdef.mask_path;

path_def.mask_folder_name=pathdef.mask_folder_name;

skip_copying_data=0;

skip_ME_merging=0;

skip_spm_preprocessing=0;

skip_movement_report=1;

skip_creating_masks=1;

skip_filtration=1;

opt.N_echoes=3;

opt.BOLD_suffix='_bold.nii';

opt.boundingbox = [-78 -112 -50; 78 76 85];

opt.SmoothFWHMmm = [6 6 6];

opt.TR=0.98;

opt.TE=[14 34.63 55.26];

use_parfor=1;

ncores=4;

subject_input='subjects.mat';

subject_info=subject_input.subject_data;

subject_info_run1=subject_info;

nsub = size(subject_info,1);

if skip_copying_data~=1

if use_parfor==1

parpool(ncores)

parfor sub_id=1:nsub

copy_data_for_preprocessing(path_def,subject_info_run1,opt,sub_id);

disp(['*****************************************************'])

disp(['Copying: Subject id ', subject_info{sub_id,1},'; ',num2str(sub_id),'/',num2str(nsub),' completed'])

disp(['*****************************************************'])

end

delete(gcp)

else

for sub_id=1:nsub

copy_data_for_preprocessing(path_def,subject_info_run1,opt,sub_id);

disp(['*****************************************************'])

disp(['Copying: Subject id ', subject_info{sub_id,1},'; ',num2str(sub_id),'/',num2str(nsub),' completed'])

disp(['*****************************************************'])

end

end

end

if skip_ME_merging~=1

if use_parfor==1

parpool(ncores)

parfor sub_id=1:nsub

ME_merging_fcn(path_def,subject_info_run1,opt,sub_id);

disp(['*****************************************************'])

disp(['ME merging: Subject id ', subject_info_run1{sub_id,1},'; variant ',subject_info_run1{sub_id,3},'; ',num2str(sub_id),'/',num2str(nsub),' completed'])

disp(['*****************************************************'])

end

delete(gcp)

else

for sub_id=1:nsub

ME_merging_fcn(path_def,subject_info_run1,opt,sub_id);

disp(['*****************************************************'])

disp(['ME merging: Subject id ', subject_info_run1{sub_id,1},'; variant ',subject_info_run1{sub_id,3},'; ',num2str(sub_id),'/',num2str(nsub),' completed'])

disp(['*****************************************************'])

end

end

end

if skip_spm_preprocessing~=1

if use_parfor==1

parpool(ncores)

parfor sub_id=1:nsub

SPM12_preprocess_MG(subject_info_run1,path_def,sub_id);

disp(['*****************************************************'])

disp(['SPM processing: Subject id ', subject_info{sub_id,1},'; ',num2str(sub_id),'/',num2str(nsub),' completed'])

disp(['*****************************************************'])

end

delete(gcp)

else

for sub_id=1:nsub

SPM12_preprocess_MG(subject_info_run1,path_def,sub_id);

disp(['*****************************************************'])

disp(['SPM processing: Subject id ', subject_info{sub_id,1},'; ',num2str(sub_id),'/',num2str(nsub),' completed'])

disp(['*****************************************************'])

% end

end

end

end

if skip_movement_report~=1

FD_threshold=1.5;

for sub=1:nsub

prepare_movement_report(subject_info_run1(sub,:),path_def,FD_threshold,'run1_before');

prepare_movement_report(subject_info_run2(sub,:),path_def,FD_threshold,'run2_after');

end

end

if skip_creating_masks~=1

intensity_mask_threshold=0.8;

filt='swu';

for sub=1:nsub

create_mask(path_def.new_folder,subject_info_run1{sub,1},subject_info_run1{sub,2},filt,subject_info_run1{sub,3},path_def.mask_folder_name,path_def.mask_path,intensity_mask_threshold,'run1_before');

create_mask(path_def.new_folder,subject_info_run2{sub,1},subject_info_run2{sub,2},filt,subject_info_run2{sub,3},path_def.mask_folder_name,path_def.mask_path,intensity_mask_threshold,'run2_after');

end

end

if skip_filtration~=1

mask_folder=fullfile(path_def.mask_path,path_def.mask_folder_name);

cwd = path_def.new_folder;

if use_parfor==1

parpool(ncores)

parfor sub_id=1:nsub

filter_parpool(sub_id,subject_info_run1,mask_folder,cwd,TR,'run1_before');

filter_parpool(sub_id,subject_info_run2,mask_folder,cwd,TR,'run2_after');

disp(['*****************************************************'])

disp(['Filtering data: Subject id ', subject_info_run1{sub_id,1},'; ',num2str(sub_id),'/',num2str(nsub),' completed'])

disp(['*****************************************************'])

end

delete(gcp)

else

for sub_id=1:nsub

filter_parpool(sub_id,subject_info_run1,mask_folder,cwd,TR,'run1_before');

filter_parpool(sub_id,subject_info_run2,mask_folder,cwd,TR,'run2_after');

disp(['*****************************************************'])

disp(['Filtering data: Subject id ', subject_info_run1{sub_id,1},'; ',num2str(sub_id),'/',num2str(nsub),' completed'])

disp(['*****************************************************'])

end

end

end

function filter_parpool(sub,subject_data,mask_folder,cwd,TR,suffix)

foldname=subject_data(:,1);

nsub=size(foldname,1);

sesname=subject_data{sub,2};

dwd=fullfile(cwd,foldname{sub,1},sesname);

cd(dwd);

data_prefix='swu';

data_prefix_WM_CSF_extraction='wu';

OutputFilePrefix='cwmr24hp';

swd=dwd;

disp(swd)

P = [];

for ses = 1:1

q = fullfile(swd,[data_prefix,subject_data{sub,3}]);

P = strvcat(P,q);

end

vols = spm_vol(P);

nscans = size(vols,1);

Y = spm_read_vols(vols);

mrfile=fullfile(swd,['rp_',subject_data{sub,3}(1:end-4),'.txt']);

rp = spm_detrend(dlmread(mrfile));

rp2=rp.^2;

rp_diff=[zeros(1,size(rp,2));diff(rp)];

rp_diff2=rp_diff.^2;

mask_path=mask_folder;

mask_name=['mask_',foldname{sub,1},'_',suffix,'.nii'];

reg_WM_CSF=create_WM_CSF_reg(dwd,subject_data{sub,3},data_prefix_WM_CSF_extraction,mask_path,mask_name,TR);

reg=[rp rp2 rp_diff rp_diff2 reg_WM_CSF];

reg=stand(reg,1);

nreg=size(reg,2);

regressstruct=[];

for i=1:1

regressstruct(:,i) = ones(nscans,1);

end

for i=2:27

regressstruct(:,i) = spm_detrend(reg(:,i-1),0);

end

K.RT = TR;

K.row = 1:nscans;

K.HParam = 128; % [s] ~ 1/128 [Hz]

K = spm_filter(K);

X = spm_filter(K,regressstruct);

Yfilt = Y;

for x=1:vols(1).dim(1)

for y=1:vols(1).dim(2)

for z = 1:vols(1).dim(3)

Y_tmp=[];

Y_tmp=squeeze(Y(x,y,z,:));

if max(Y_tmp)~=0

Y_tmp=spm_detrend(Y_tmp,1);

Y_tmp_f = spm_filter(K,Y_tmp);

Y_tmp_f = spm_detrend(Y_tmp_f,1);

Yfilt(x,y,z,:) = mean(Y_tmp_f - X*(pinv(full(X))*Y_tmp_f),2);

end

end

end

end

vfilt=vols;

for scan = 1:nscans

[fpath, fname, fext] = fileparts(vfilt(scan).fname);

newfname = [OutputFilePrefix fname fext];

vfilt(scan).fname = fullfile(fpath,newfname);

vfilt(scan).dt(1) = 16;

vfilt(scan) = spm_create_vol(vfilt(scan));

vfilt(scan) = spm_write_vol(vfilt(scan),Yfilt(:,:,:,scan));

end

disp(' ')

disp(['Sub ',num2str(sub),' / ',num2str(nsub),' Regression completed'])

disp(' ')

function flag=create_mask(study_path,subject,subfolder_name,filt,data_name,mask_folder_name,mask_path,intensity_mask_threshold,suffix)

flag=0;

data_path=fullfile(study_path,subject,subfolder_name);

files = fullfile(data_path,[filt,data_name]);

%% Compute global means and thresholds

% get data

%==========================================================================

% scan number

%--------------------------------------------------------------------------

xM.I=0; % xM.I - Implicit masking (0=>none, 1 => implicit zero/NaN mask)

%-Map files

%--------------------------------------------------------------------------

fprintf('%-40s: ','Mapping files') %-#

VY = spm_vol(files);

fprintf('%30s\n','...done') %-#

nScan = size(VY,1);

%-check internal consistency of images

%--------------------------------------------------------------------------

spm_check_orientations(VY);

%-Compute Global variate

%==========================================================================

GM = 100;

q = length(VY);

g = zeros(q,1);

fprintf('%-40s: %30s','Calculating globals',' ') %-#

for i = 1:q

fprintf('%s%30s',repmat(sprintf('\b'),1,30),sprintf('%4d/%-4d',i,q))%-#

g(i) = spm_global(VY(i)); % Computing global mean for every volume

end

fprintf('%s%30s\n',repmat(sprintf('\b'),1,30),'...done') %-#

% scale if specified (otherwise session specific grand mean scaling)

%--------------------------------------------------------------------------

gSF = GM./mean(g); % Default in SPM - grand mean scaling - if SPM.xGX.iGXcalc is 'none'

% gSF = GM./g; % If specified - if SPM.xGX.iGXcalc is not 'none'

%-Apply gSF to memory-mapped scalefactors to implement scaling

%--------------------------------------------------------------------------

for i = 1:q

VY(i).pinfo(1:2,:) = VY(i).pinfo(1:2,:)*gSF; % Scaling

end

%-Masking structure automatically set to 80% of mean - TH threshold computing

%==========================================================================

% try

% TH = g.*gSF*spm_get_defaults('mask.thresh');

% catch

% TH = g.*gSF*0.8;

% end

TH = g.*gSF*intensity_mask_threshold; % User can specify the threshold

%% spm_spm

SVNid = '$Rev: 4515 $';

SPMid = spm('FnBanner',mfilename,SVNid);

Finter = spm('FigName','Stats: estimation...'); spm('Pointer','Watch');

%-Change to SPM.swd if specified

%--------------------------------------------------------------------------

SPM.swd = pwd;

%==========================================================================

% - A N A L Y S I S P R E L I M I N A R I E S

%==========================================================================

%-Get non-sphericity V

%==========================================================================

%-Image dimensions and data

%==========================================================================

spm_check_orientations(VY);

% check files exists and try pwd

%--------------------------------------------------------------------------

for i = 1:numel(VY)

if ~spm_existfile(VY(i).fname)

[p,n,e] = fileparts(VY(i).fname);

VY(i).fname = [n,e];

end

end

M = VY(1).mat;

DIM = VY(1).dim(1:3)';

xdim = DIM(1); ydim = DIM(2); zdim = DIM(3);

YNaNrep = spm_type(VY(1).dt(1),'nanrep');

%-Maximum number of residual images for smoothness estimation

%--------------------------------------------------------------------------

MAXRES = spm_get_defaults('stats.maxres');

fprintf('%s%30s\n',repmat(sprintf('\b'),1,30),'...done'); %-#

%-Initialise output images (unless this is a 1st pass for ReML)

%==========================================================================

fprintf('%-40s: %30s','Output images','...initialising'); %-#

%-Initialise new mask name: current mask & conditions on voxels

%----------------------------------------------------------------------

% Create folder for mask

cd(mask_path);

% folder_name=fullfile(mask_path,mask_folder_name);

mkdir(mask_folder_name);

mask_path=fullfile(mask_path,mask_folder_name);

mask_name=['mask_' subject '_' suffix '.nii'];

cd(mask_path);

VM = struct('fname', mask_name,...

'dim', DIM',...

'dt', [spm_type('uint8') spm_platform('bigend')],...

'mat', M,...

'pinfo', [1 0 0]',...

'descrip','spm_spm:resultant analysis mask');

VM = spm_create_vol(VM);

fprintf('%s%30s\n',repmat(sprintf('\b'),1,30),'...initialised'); %-#

%==========================================================================

% - F I T M O D E L & W R I T E P A R A M E T E R I M A G E S

%==========================================================================

%-MAXMEM is the maximum amount of data processed at a time (bytes)

%--------------------------------------------------------------------------

MAXMEM = spm_get_defaults('stats.maxmem');

mmv = MAXMEM/8/nScan;

blksz = min(xdim*ydim,ceil(mmv)); %-block size

nbch = ceil(xdim*ydim/blksz); %-# blocks

nbz = max(1,min(zdim,floor(mmv/(xdim*ydim)))); nbz = 1; %-# planes

blksz = blksz * nbz;

%-Initialise variables used in the loop

%==========================================================================

[xords, yords] = ndgrid(1:xdim, 1:ydim);

xords = xords(:)'; yords = yords(:)'; % plane X,Y coordinates

S = 0; % Volume (voxels)

%-Initialise XYZ matrix of in-mask voxel co-ordinates (real space)

%--------------------------------------------------------------------------

XYZ = zeros(3,xdim*ydim*zdim);

%-Cycle over bunches blocks within planes to avoid memory problems

%==========================================================================

str='mask estimation';

spm_progress_bar('Init',100,str,'');

for z = 1:nbz:zdim %-loop over planes (2D or 3D data)

% current plane-specific parameters

%----------------------------------------------------------------------

CrPl = z:min(z+nbz-1,zdim); %-plane list

zords = CrPl(:)*ones(1,xdim*ydim); %-plane Z coordinates

Q = []; %-in mask indices for this plane

for bch = 1:nbch %-loop over blocks

%-Print progress information in command window

%------------------------------------------------------------------

if numel(CrPl) == 1

str = sprintf('Plane %3d/%-3d, block %3d/%-3d',...

z,zdim,bch,nbch);

else

str = sprintf('Planes %3d-%-3d/%-3d',z,CrPl(end),zdim);

end

if z == 1 && bch == 1

str2 = '';

else

str2 = repmat(sprintf('\b'),1,72);

end

fprintf('%s%-40s: %30s',str2,str,' ');

%-construct list of voxels in this block

%------------------------------------------------------------------

I = (1:blksz) + (bch - 1)*blksz; %-voxel indices

I = I(I <= numel(CrPl)*xdim*ydim); %-truncate

xyz = [repmat(xords,1,numel(CrPl)); ...

repmat(yords,1,numel(CrPl)); ...

reshape(zords',1,[])];

xyz = xyz(:,I); %-voxel coordinates

nVox = size(xyz,2); %-number of voxels

%-Get data & construct analysis mask

%=================================================================

fprintf('%s%30s',repmat(sprintf('\b'),1,30),'...read & mask data')

Cm = true(1,nVox); %-current mask

%-Get the data in mask, compute threshold & implicit masks

%------------------------------------------------------------------

Y = zeros(nScan,nVox);

for i = 1:nScan

%-Load data in mask

%--------------------------------------------------------------

if ~any(Cm)

break

end %-Break if empty mask

Y(i,Cm) = spm_get_data(VY(i),xyz(:,Cm),false);

Cm(Cm) = Y(i,Cm) > TH(i); %-Threshold (& NaN) mask

if xM.I && ~YNaNrep && TH(i) < 0 %-Use implicit mask

Cm(Cm) = abs(Y(i,Cm)) > eps;

end

end

%-Mask out voxels where data is constant

%------------------------------------------------------------------

Cm(Cm) = any(diff(Y(:,Cm),1));

CrS = sum(Cm); %-# current voxels

%==================================================================

%-Proceed with General Linear Model (if there are voxels)

%==================================================================

if CrS

clear Y %-Clear to save memory

end % (CrS)

%-Append new inmask voxel locations and volumes

%------------------------------------------------------------------

XYZ(:,S + (1:CrS)) = xyz(:,Cm); %-InMask XYZ voxel coords

Q = [Q I(Cm)]; %-InMask XYZ voxel indices

S = S + CrS; %-Volume analysed (voxels)

end % (bch)

%-Plane complete, write plane to image files (unless 1st pass)

%======================================================================

fprintf('%s%30s',repmat(sprintf('\b'),1,30),'...saving plane'); %-#

jj = NaN(xdim,ydim,numel(CrPl));

%-Write Mask image

%------------------------------------------------------------------

if ~isempty(Q)

jj(Q) = 1;

end

VM = spm_write_plane(VM, ~isnan(jj), CrPl);

%-Report progress

%----------------------------------------------------------------------

fprintf('%s%30s',repmat(sprintf('\b'),1,30),'...done'); %-#

spm_progress_bar('Set',100*(bch + nbch*(z - 1))/(nbch*zdim));

end % (for z = 1:zdim)

fprintf('\n'); %-#

spm_progress_bar('Clear')

% scsource = [mfilename('fullpath') '.m'];

% scname= [mfilename '.m.txt'];

% copyfile(scsource,fullfile(mask_path,scname));

%==========================================================================

% - P O S T E S T I M A T I O N C L E A N U P

%==========================================================================

if S == 0, spm('alert!','No inmask voxels - empty analysis!');

return;

end

%==========================================================================

%- E N D: Cleanup GUI

%==========================================================================

fprintf('%s%30s\n',repmat(sprintf('\b'),1,30),'...done') %-#

spm('FigName','Stats: done',Finter); spm('Pointer','Arrow')

fprintf('%-40s: %30s\n','Completed',spm('time')) %-#

flag=1;

% end

function copy_data_for_preprocessing(path_def,subject_data,opt,sub_id)

parent_folder=path_def.orig_folder;

output_folder=path_def.new_folder;

ses_col=3;

N_echoes=opt.N_echoes;

orig_folder=fullfile(parent_folder,subject_data{sub_id,1},subject_data{sub_id,2});

new_dest=fullfile(output_folder,subject_data{sub_id,1},subject_data{sub_id,2});

if ~exist(new_dest,'dir')

mkdir(new_dest)

end

filename=cell(N_echoes,1);

filename{1,1}=subject_data{sub_id,ses_col};

suffix=opt.BOLD_suffix;

if N_echoes>1

for ii=2:N_echoes

tmp=subject_data{sub_id,ses_col};

k=strfind(tmp,suffix);

tmp(k-1)=num2str(ii);

filename{ii,1}=tmp;

end

end

for ii=1:N_echoes

tmp_source=fullfile(orig_folder,filename{ii,1});

tmp_target=fullfile(new_dest,filename{ii,1});

copyfile(tmp_source,tmp_target);

end

%% json

for ii=1:N_echoes

tmp_file=filename{ii,1};

tmp_file2=[tmp_file(1:end-4),'.json'];

tmp_source=fullfile(orig_folder,tmp_file2);

tmp_target=fullfile(new_dest,tmp_file2);

copyfile(tmp_source,tmp_target);

end

function prepare_movement_report(subject_info,path_def,FD_threshold,run_name)

cwd=fullfile(path_def.new_folder,subject_info{1,1},subject_info{1,2});

rp_file_name=['rp_',subject_info{1,3}(1:end-4),'.txt'];

rp_file_full_path=fullfile(cwd,rp_file_name);

input_is_in_radians=1;

FD_threshold_ex_scans=FD_threshold;

no_of_added_scans_after_ex=0;

perc_of_affected_scans_th=0;

mov_par1=load(rp_file_full_path);

nscans=size(mov_par1,1);

nsub=size(subject_info,1);

try

nses=length(subject_info(1,2));

catch

sesname={'tmp_sess'};

nses=length(sesname);

end

nparams=6;% 6 movement parameters - 3 translations and 3 rotations

param_desc={'trans x','trans y','trans z','rot x','rot y','rot z'};

mov_cell=cell(nsub,nparams); % cell with regresors

mov_matrix=cell(1,nparams);

max_mov_params=NaN(nsub,nparams);

max_diff_mov_params=NaN(nsub,nparams);

max_sum_mov_params=NaN(nsub,nparams);

for i=1:nparams

mov_matrix{i}=NaN(nscans,nsub); % For visualization

end

FD=NaN(nscans,nsub,nses);

fd_type=1;

mov_par_matrix=NaN(nscans,6,nsub,nses);

for sub=1:nsub

for ses=1:nses

mov_txt = rp_file_full_path;

mov_par=load(mov_txt);

if input_is_in_radians==1

mov_par(:,4:6)=(180/pi).*mov_par(:,4:6);

end

Nactual_scans=size(mov_par,1);

mov_par_matrix(1:Nactual_scans,:,sub,ses)=mov_par;

for i=1:nparams

mov_cell{sub,i}=mov_par(1:Nactual_scans,i);

mov_matrix{i}(1:Nactual_scans,sub)=mov_par(1:Nactual_scans,i);

end

max_mov_params(sub,:)=max(abs(mov_par));

max_diff_mov_params(sub,:)=max(abs(diff(mov_par)));

max_sum_mov_params(sub,:)=sum(abs(mov_par));

FD(1:Nactual_scans,sub,ses)=get_FD(mov_par,fd_type);

end

end

excluded_scans=cell(0,3);

id=0;

for sub=1:nsub

for ses=1:nses

clear tmp_excluded

tmp_FD=squeeze(FD(:,sub,ses));

tmp_N=numel(tmp_FD);

tmp_ind_ex=find(tmp_FD>FD_threshold_ex_scans);

if ~isempty(tmp_ind_ex)

id=id+1;

n_ex=numel(tmp_ind_ex);

tmp_perc=100*n_ex/tmp_N;

tmp_excluded=[];

for i=1:n_ex

tmp_excluded=[tmp_excluded tmp_ind_ex(i)];

for j=1:no_of_added_scans_after_ex

tmp_excluded=[tmp_excluded tmp_ind_ex(i)+j];

end

end

tmp_excluded=unique(tmp_excluded);

excluded_scans{id,1}=tmp_perc;

excluded_scans{id,2}=numel(tmp_excluded);

excluded_scans{id,3}=subject_info{sub,1};

excluded_scans{id,4}=tmp_excluded;

end

end

end

mov_par_diff=cell(1,nparams);

for i=1:nparams

mov_par_diff{i}=[zeros(1,nsub);mov_matrix{i}(2:end,:)-mov_matrix{i}(1:(end-1),:)];

end

foldname=subject_info(1,1);

excluded_scans=cell(0,3);

id=0;

for sub=1:nsub

for ses=1:nses

clear tmp_excluded

tmp_FD=squeeze(FD(:,sub,ses));

tmp_N=numel(tmp_FD);

tmp_ind_ex=find(tmp_FD>FD_threshold_ex_scans);

if ~isempty(tmp_ind_ex)

id=id+1;

n_ex=numel(tmp_ind_ex);

tmp_perc=100*n_ex/tmp_N;

tmp_excluded=[];

for i=1:n_ex

tmp_excluded=[tmp_excluded tmp_ind_ex(i)];

for j=1:no_of_added_scans_after_ex

tmp_excluded=[tmp_excluded tmp_ind_ex(i)+j];

end

end

tmp_excluded=unique(tmp_excluded);

GML_reg=create_GLM_reg(tmp_excluded,nscans);

excluded_scans{id,1}=tmp_perc;

excluded_scans{id,2}=numel(tmp_excluded);

excluded_scans{id,3}=foldname{sub,1};

excluded_scans{id,4}=num2str(tmp_excluded);

excluded_scans{id,5}=GML_reg;

end

end

end

xlegend={'Selected threshold' 'Percent of affected scans' 'Number of scans' 'Dataset' 'Excluded scans' };

threshold_cell{1,1}=['FD <= ', num2str(FD_threshold),' mm'];

threshold_cell{2,1}=['Affected scans <= ', num2str(perc_of_affected_scans_th),'%'];

res_name=['Movement_info_report_',subject_info{1,1},'_',run_name,'.mat'];

output_name=fullfile(cwd,res_name);

excluded_scans_description=xlegend(2:end);

save(output_name,'excluded_scans_description','excluded_scans','threshold_cell')

function FD=get_FD(mov_par,fd_type)

% Framewise displacement FD

% Regressors

reg_trans = mov_par(:,1:3);

reg_rot = mov_par(:,4:6);

dif_trans = [0 0 0; (reg_trans(2:end,:)-reg_trans(1:(end-1),:))];

dif_rot = [0 0 0; (reg_rot(2:end,:)-reg_rot(1:(end-1),:))];

% sqr_dif_trans = dif_trans.^2;

% sqr_dif_rot = dif_rot.^2;

% Framewise Displacement

switch fd_type

case 1 % Jonathan Power

abs_trans = abs(dif_trans);

abs_rot = abs(dif_rot);

% Rotational displacements were converted from degrees to

% millimeters by calculating displacement on the surface of a

% sphere of radius 50 mm, which is approximately the mean distance

% from the cerebral cortex to the center of the head. - z článku

% Power 2012 Spurious but systematic correlations in functional connectivity MRI networks arise from subject motion

fd = abs_trans(:,1)+abs_trans(:,2)+abs_trans(:,3)+50*pi/180*(abs_rot(:,1)+abs_rot(:,2)+abs_rot(:,3)); % Degrees are here transformed to radians

case 2 % Chao-Gan Yan

fd = sqrt((dif_trans(:,1).^2)+(dif_trans(:,2).^2)+(dif_trans(:,3).^2));

case 3 % Koene Van Dijk

fd = sqrt((reg_trans(2:end,1).^2)+(reg_trans(2:end,2).^2)+(reg_trans(2:end,3).^2))-sqrt((reg_trans(1:(end-1),1).^2)+(reg_trans(1:(end-1),2).^2)+(reg_trans(1:(end-1),3).^2));

fd = [0; fd];

end

FD=fd;

function GML_reg=create_GLM_reg(excluded_scans,nscans)

n_excluded=numel(excluded_scans);

GML_reg=zeros(nscans,n_excluded);

for i=1:n_excluded

GML_reg(excluded_scans(i),i)=1;

end

function SPM12_preprocess_MG(subject_info,path_def,sub_id)

spm_jobman('initcfg');

orig_folder = path_def.orig_folder;

new_folder = path_def.new_folder;

ses_col=3;

pathdelim = filesep;

subjects=subject_info(sub_id,1);

nsub=size(subjects,1);

reslicevoxelsize = [3 3 3];

boundingbox = [-78 -112 -50; 78 76 85];

SmoothFWHMmm = [6 6 6];

tic

for sub=1:nsub

for ses=1

disp('')

scsource = [mfilename('fullpath') '.m'];

scname= [mfilename '.m.txt'];

scdestination = fullfile(new_folder,subjects{sub,1},scname);

swd=[new_folder pathdelim subjects{sub,1} pathdelim subject_info{sub,2}];

swd_new = swd;

copyfile(scsource,scdestination);

fMRI_file=subject_info{sub_id,ses_col};

fMRI_input=fullfile(swd,fMRI_file);

tmp_N = nifti(fMRI_input);

dim = [tmp_N.dat.dim 1 1 1 1 1];

nscans = dim(4);

% get files to realign

%%%%%%%%%%%%%%%%%%%%%%%%%%%%%%%%%%%%%%%%%%%%%%%%%%%%%%%%%%%%%

% Set up Realign and unwarp

%%%%%%%%%%%%%%%%%%%%%%%%%%%%%%%%%%%%%%%%%%%%%%%%%%%%%%%%%%%%%

do_realign=0;

if do_realign==1

for i=1:nscans

selected_scans{i,1} = [fMRI_input,',',num2str(i)];

end

RW.data.scans = selected_scans;

RW.data.pmscan = '';

RW.eoptions = struct( 'quality', 0.9,...

'sep', 4,...

'fwhm', 5,...

'rtm', 0,...

'einterp', 2,...

'ewrap', [0 0 0],...

'weight', {''});

RW.uweoptions = struct( 'basfcn', [12 12],...

'regorder', 1,...

'lambda', 100000,...

'jm', 0,...

'fot', [4 5],...

'sot', {[]},...

'uwfwhm', 4,...

'rem', 1,...

'noi', 5,...

'expround', 'Average');

RW.uwroptions = struct( 'uwwhich', [2 1],...

'rinterp', 4,...

'wrap', [0 0 0],...

'mask', 1,...

'prefix', 'u');

matlabbatch{1}.spm.spatial.realignunwarp = RW;

spm_jobman('run',matlabbatch);

clear matlabbatch;

end

selected_scans = [];

tmp_col=ses_col;

tmp_session=subject_info{sub_id,tmp_col};

q=fullfile(new_folder, subject_info{sub_id,1},subject_info{sub_id,2},['tse_r',subject_info{sub_id,3}] );

tmpv = spm_vol(q);

tmpY = spm_read_vols(tmpv);

meanY = mean(tmpY,4);

meanvol = tmpv(1);

fname=meanvol.fname;

[pathstr,name,ext]=fileparts(fname);

meanvol.fname=fullfile(pathstr,['mean' name ext]);

meanvol.descrip=['mean image'];

meanvol=spm_write_vol(meanvol,meanY);

%%%%%%%%%%%%%%%%%%%%%%%%%%%%%%%%%%%%%%%%%%%%

% Spatial normalisation fMRI

%%%%%%%%%%%%%%%%%%%%%%%%%%%%%%%%%%%%%%%%%%%%

srcimg{1} = fullfile(swd_new,['meantse_r',fMRI_file]);

SUBstr.vol = srcimg;

tmpf=[];

tmpf = strvcat(tmpf, fullfile(swd_new,['tse_r',fMRI_file]));

tmpf = strvcat(tmpf, fullfile(swd_new,['meantse_r',fMRI_file]));

resamp=cellstr(tmpf);

SUBstr.resample = resamp;

EOstr = struct ('biasreg', 0.0001,...

'biasfwhm', 60,...

'tpm', {[spm('dir') '\tpm\TPM.nii']},...

'affreg', 'mni',...

'reg', [0 0.001 0.5 0.05 0.2],...

'fwhm', 0,...

'samp', 3);

WOstr.bb = boundingbox;

WOstr.vox = reslicevoxelsize;

WOstr.interp = 4;

ESTWR = struct ('subj', SUBstr,...

'eoptions', EOstr,...

'woptions', WOstr);

matlabbatch{1}.spm.spatial.normalise.estwrite = ESTWR;

spm_jobman('run',matlabbatch);

clear matlabbatch SUBstr WOstr EOstr ESTWR;%

%%%%%%%%%%%%%%%%%%%%%%%%%%%%%%%%%%%%%%%%%%%%%%%%%%%%%%%%%%%%%

% Set up Smooth functional images

%%%%%%%%%%%%%%%%%%%%%%%%%%%%%%%%%%%%%%%%%%%%%%%%%%%%%%%%%%%%%

P = [];

q = fullfile(swd_new,['wtse_r',fMRI_file]);

P = strvcat(P,q);

for i=1:size(P,1)

selected_scans{i,1} = [P(i,:)];

end

SMT.data = selected_scans;

SMT.fwhm = SmoothFWHMmm;

SMT.dtype = 0;

SMT.im = 0;

SMT.prefix = 's';

matlabbatch{1}.spm.spatial.smooth = SMT;

spm_jobman('run',matlabbatch);

clear matlabbatch;

%************************************************************

clear P dirref direc SMT dirsrc srcimg refimg othimg COR RW q matlabbatch selected_scans RW COR NRA NRF WRITE WOstr SUBstr resamp;

end

end

toc

function reg_WM_CSF=create_WM_CSF_reg(datacwd,BOLD_filename,data_filter,mask_path,mask_name,TR)

template_path=fullfile(spm('dir'),'tpm','TPM.nii');

tmp_WM_id=2;

tmp_CSF_id=3;

data_name=fullfile(datacwd,[data_filter,BOLD_filename]);

data_vol=spm_vol(data_name);

[data,~]=spm_read_vols(data_vol);

tmp_vol=spm_vol(template_path);

[tmp_data,~]=spm_read_vols(tmp_vol);

mask_vol=spm_vol(fullfile(mask_path,mask_name));

[mask_data,~]=spm_read_vols(mask_vol);

reg_WM=get_reg_vals(tmp_WM_id,data_vol,template_path,data,mask_data,mask_path,mask_name,TR);

reg_CSF=get_reg_vals(tmp_CSF_id,data_vol,template_path,data,mask_data,mask_path,mask_name,TR);

reg_WM_CSF=[reg_WM,reg_CSF];

function reg=get_reg_vals(id,data_vol,template_path,data,mask_data,mask_path,mask_name,TR)

nscans=size(data,4);

threshold=0.9;

Q_mask=find(mask_data == 1);

P{1} = fullfile(mask_path,mask_name);

P{2} = template_path;

Pi=strvcat(P{1}, P{2});

[Qo,template_Vo] = spm_reslice_MG(Pi);

[tmp_Y,tmp_XYZ] = spm_read_vols(template_Vo);

tmp_Y=squeeze(tmp_Y(:,:,:,id));

Q1 = find(tmp_Y > threshold);

Q3 = Q1;

Q3(~ismember(Q1,Q_mask))=[];

tmp_mm=tmp_XYZ(:,Q3);

mx1=[tmp_mm; ones(1,size(tmp_mm,2))];

tmp_vx=data_vol(1).mat\mx1;

tmp_vx=tmp_vx(1:3,:);

tmp_BOLD = spm_get_data(data_vol,tmp_vx);

y = spm_detrend(tmp_BOLD,1);

K.RT = TR;

K.row = 1:nscans;

K.HParam = 128;

K = spm_filter(K);

y = spm_filter(K,y);

ex=[];

for in=1:size(y,2)

if sum(isnan(y(:,in)))>0

ex(end+1)=in;

end

end

y(:,ex)=[];

[reg,~]=PCA_MG(y);

reg=reg(:,1);

function [components,var]=PCA_MG(Y)

% Function performs PCA analysis using svd. Based on SPM toolbox.

% Input: Y - n signals for PCA decomposition - size Y:(signal length, n)

% Output: components - sorted N components - size components:(signal length, n)

% var - explained variability [%] for every component - size components:(1, n)

% ------------------------------------------------------------------------

% Based on svd used in SPM toolbox

% Version 1.01 - use spm_detrend instead of ica_tb_detrend

% ------------------------------------------------------------------------

% Written by Martin Gajdos

[m,n] = size(Y);

Y = spm_detrend(Y,1);

if m > n

[v s v] = svd(Y'*Y);

s = diag(s);

s_perc=100*(s/sum(s));

v = v(:,1:numel(s_perc));

u = NaN(m,numel(s_perc));

for i=1:numel(s_perc)

u(:,i) = Y*v(:,i)/sqrt(s(i));

end

else

[u s u] = svd(Y*Y');

s = diag(s);

s_perc=100*(s/sum(s));

u = u(:,1:numel(s_perc));

v = NaN(n,numel(s_perc));

for i=1:numel(s_perc)

v(:,i) = Y'*u(:,i)/sqrt(s(i));

end

end

d = sign(sum(v));

u = u.*repmat(d,m,1);

components=NaN(m,numel(s_perc));

var=s_perc;

for i=1:numel(s_perc)

components(:,i) = u(:,i)*sqrt(s(i)/n);

end

function A = stand(A,flag)

if ~exist('flag','var')

flag = 1;

end

switch flag

case 0 % remove mean

A = A - repmat(mean(A),size(A,1),1);

case 1 % remove mean and normalize to std=1

A = A - repmat(mean(A),size(A,1),1);

A = A ./ repmat(std(A),size(A,1),1);

case 2 % normalize to std=1

A = A ./ repmat(std(A),size(A,1),1);

case 3 % remove mean, normalize to std=1, add mean

mA = mean(A);

A = A - repmat(mA,size(A,1),1);

A = A ./ repmat(std(A),size(A,1),1);

A = A + repmat(mA,size(A,1),1);

end

function [resliced_name,resliced_vol]=spm_reslice_MG(P,flags)

% Function spm_reslice edited by MG 23.1.2015. Changed type of saving of

% resliced image. All images are resliced to the first image.

%

%__________________________________________________________________________

%

% function spm_reslice(P,flags)

% Rigid body reslicing of images

% FORMAT spm_reslice(P,flags)

%

% P - matrix or cell array of filenames {one string per row}

% All operations are performed relative to the first image.

% ie. Coregistration is to the first image, and resampling

% of images is into the space of the first image.

%

% flags - a structure containing various options. The fields are:

%

% mask - mask output images (true/false) [default: true]

% To avoid artifactual movement-related variance the

% realigned set of images can be internally masked, within

% the set (i.e. if any image has a zero value at a voxel

% than all images have zero values at that voxel). Zero

% values occur when regions 'outside' the image are moved

% 'inside' the image during realignment.

%

% mean - write mean image (true/false) [default: true]

% The average of all the realigned scans is written to

% an image file with 'mean' prefix.

%

% interp - the B-spline interpolation method [default: 1]

% Non-finite values result in Fourier interpolation. Note

% that Fourier interpolation only works for purely rigid

% body transformations. Voxel sizes must all be identical

% and isotropic.

%

% which - values of 0, 1 or 2 are allowed [default: 2]

% 0 - don't create any resliced images.

% Useful if you only want a mean resliced image.

% 1 - don't reslice the first image.

% The first image is not actually moved, so it may

% not be necessary to resample it.

% 2 - reslice all the images.

% If which is a 2-element vector, flags.mean will be set

% to flags.which(2).

%

% wrap - three values of either 0 or 1, representing wrapping in

% each of the dimensions. For fMRI, [1 1 0] would be used.

% For PET, it would be [0 0 0]. [default: [0 0 0]]

%

% prefix - prefix for resliced images [default: 'r']

%

%__________________________________________________________________________

%

% The spatially realigned images are written to the original subdirectory

% with the same (prefixed) filename. They are all aligned with the first.

%

% Inputs:

% A series of images conforming to SPM data format (see 'Data Format'). The

% relative displacement of the images is stored in their header.

%

% Outputs:

% The routine uses information in their headers and writes the realigned

% image files to the same subdirectory with a prefix.

%__________________________________________________________________________

% Copyright (C) 1999-2011 Wellcome Trust Centre for Neuroimaging

% John Ashburner

% $Id: spm_reslice.m 4490 2011-09-14 16:22:27Z guillaume $

%__________________________________________________________________________

%

% The headers of the images contain a 4x4 affine transformation matrix 'M',

% usually affected by the `realignment' and `coregistration' modules.

% What these matrices contain is a mapping from the voxel coordinates

% (x0,y0,z0) (where the first voxel is at coordinate (1,1,1)), to

% coordinates in millimeters (x1,y1,z1).

%

% x1 = M(1,1)*x0 + M(1,2)*y0 + M(1,3)*z0 + M(1,4)

% y1 = M(2,1)*x0 + M(2,2)*y0 + M(2,3)*z0 + M(2,4)

% z1 = M(3,1)*x0 + M(3,2)*y0 + M(3,3)*z0 + M(3,4)

%

% Assuming that image1 has a transformation matrix M1, and image2 has a

% transformation matrix M2, the mapping from image1 to image2 is: M2\M1

% (ie. from the coordinate system of image1 into millimeters, followed

% by a mapping from millimeters into the space of image2).

%

% Several spatial transformations (realignment, coregistration,

% normalisation) can be combined into a single operation (without the

% necessity of resampling the images several times).

%__________________________________________________________________________

%

% Refs:

%

% Friston KJ, Williams SR, Howard R Frackowiak RSJ and Turner R (1995)

% Movement-related effect in fMRI time-series. Mag. Res. Med. 35:346-355

%

% W. F. Eddy, M. Fitzgerald and D. C. Noll (1996) Improved Image

% Registration by Using Fourier Interpolation. Mag. Res. Med. 36(6):923-931

%

% R. W. Cox and A. Jesmanowicz (1999) Real-Time 3D Image Registration

% for Functional MRI. Mag. Res. Med. 42(6):1014-1018

%__________________________________________________________________________

SVNid = '$Rev: 4490 $';

%-Say hello

%--------------------------------------------------------------------------

SPMid = spm('FnBanner',mfilename,SVNid);

%-Parameters

%--------------------------------------------------------------------------

if ~nargin || isempty(P), P = spm_select([2 Inf],'image'); end

if iscellstr(P), P = char(P); end

if ischar(P), P = spm_vol(P); end

def_flags = spm_get_defaults('realign.write');

% MG 23.1.2015

%--------------------------------------------------------------------------

def_flags.prefix = 'r';

% def_flags.prefix = flags.prefix;

%--------------------------------------------------------------------------

if nargin < 2

flags = def_flags;

else

fnms = fieldnames(def_flags);

for i=1:length(fnms)

if ~isfield(flags,fnms{i})

flags.(fnms{i}) = def_flags.(fnms{i});

end

end

end

if numel(flags.which) == 2

flags.mean = flags.which(2);

flags.which = flags.which(1);

elseif ~isfield(flags,'mean')

flags.mean = 1;

end

% MG 23.1.2015

%--------------------------------------------------------------------------

flags.mask=0;

flags.mean=0;

flags.interp=0;

flags.which=1;

flags.wrap=[1 1 0];

flags.prefix='tmp';

%--------------------------------------------------------------------------

%-Reslice

%--------------------------------------------------------------------------

reslice_images(P,flags);

% MG 23.1.2015

%--------------------------------------------------------------------------

resliced_name=[flags.prefix,'.nii'];

resliced_vol=spm_vol(resliced_name);

%--------------------------------------------------------------------------

fprintf('%-40s: %30s\n','Completed',spm('time')) %-#

%==========================================================================

%-function reslice_images(P,flags)

%==========================================================================

function reslice_images(P,flags)

% Reslice images volume by volume

% FORMAT reslice_images(P,flags)

% See main function for a description of the input parameters

if ~isfinite(flags.interp), % Use Fourier method

% Check for non-rigid transformations in the matrixes

for i=1:numel(P)

pp = P(1).mat\P(i).mat;

if any(abs(svd(pp(1:3,1:3))-1)>1e-7)

fprintf('\n Zooms or shears appear to be needed');

fprintf('\n (probably due to non-isotropic voxels).');

fprintf('\n These can not yet be done using the');

fprintf('\n Fourier reslicing method. Switching to');

fprintf('\n 7th degree B-spline interpolation instead.\n\n');

flags.interp = 7;

break

end

end

end

if flags.mask || flags.mean

spm_progress_bar('Init',P(1).dim(3),'Computing available voxels','planes completed');

x1 = repmat((1:P(1).dim(1))',1,P(1).dim(2));

x2 = repmat( 1:P(1).dim(2) ,P(1).dim(1),1);

if flags.mean

Count = zeros(P(1).dim(1:3));

Integral = zeros(P(1).dim(1:3));

end

if flags.mask, msk = cell(P(1).dim(3),1); end;

for x3 = 1:P(1).dim(3)

tmp = zeros(P(1).dim(1:2));

for i = 1:numel(P)

tmp = tmp + getmask(inv(P(1).mat\P(i).mat),x1,x2,x3,P(i).dim(1:3),flags.wrap);

end

if flags.mask, msk{x3} = find(tmp ~= numel(P)); end;

if flags.mean, Count(:,:,x3) = tmp; end;

spm_progress_bar('Set',x3);

end

end

nread = numel(P);

if ~flags.mean

if flags.which == 1, nread = nread - 1; end;

if flags.which == 0, nread = 0; end;

end

spm_progress_bar('Init',nread,'Reslicing','volumes completed');

[x1,x2] = ndgrid(1:P(1).dim(1),1:P(1).dim(2));

nread = 0;

d = [flags.interp*[1 1 1]' flags.wrap(:)];

for i = 1:numel(P)

if (i>1 && flags.which==1) || flags.which==2

write_vol = 1;

else

write_vol = 0;

end

if write_vol || flags.mean

read_vol = 1;

else

read_vol = 0;

end

if read_vol

if ~isfinite(flags.interp)

v = abs(kspace3d(spm_bsplinc(P(i),[0 0 0 ; 0 0 0]'),P(1).mat\P(i).mat));

for x3 = 1:P(1).dim(3)

if flags.mean

Integral(:,:,x3) = ...

Integral(:,:,x3) + ...

nan2zero(v(:,:,x3) .* ...

getmask(inv(P(1).mat\P(i).mat),x1,x2,x3,P(i).dim(1:3),flags.wrap));

end

if flags.mask

tmp = v(:,:,x3); tmp(msk{x3}) = NaN; v(:,:,x3) = tmp;

end

end

else

C = spm_bsplinc(P(i), d);

v = zeros(P(1).dim);

for x3 = 1:P(1).dim(3)

[tmp,y1,y2,y3] = getmask(inv(P(1).mat\P(i).mat),x1,x2,x3,P(i).dim(1:3),flags.wrap);

v(:,:,x3) = spm_bsplins(C, y1,y2,y3, d);

% v(~tmp) = 0;

if flags.mean

Integral(:,:,x3) = Integral(:,:,x3) + nan2zero(v(:,:,x3));

end

if flags.mask

tmp = v(:,:,x3); tmp(msk{x3}) = NaN; v(:,:,x3) = tmp;

end

end

end

if write_vol

VO = P(i);

try

VO.fname = spm_file([flags.prefix,'.nii']); % ZmÄ›na - definice mĂ­sta uloĹľenĂ­

catch

VO.fname = spm_file(P(i).fname, 'prefix',flags.prefix);

end

VO.dim = P(1).dim(1:3);

VO.dt = P(i).dt;

% VO.dt = [spm_type('int16') P(i).dt(2)];

VO.pinfo = P(i).pinfo;

VO.mat = P(1).mat;

VO.descrip = 'spm - realigned';

VO = spm_write_vol(VO,v);

end

nread = nread + 1;

end

spm_progress_bar('Set',nread);

end

if flags.mean

% Write integral image (16 bit signed)

%----------------------------------------------------------------------

Integral = Integral./Count;

PO = P(1);

PO = rmfield(PO,'pinfo');

PO.fname = spm_file(P(1).fname, 'prefix','mean');

PO.pinfo = [max(max(max(Integral)))/32767 0 0]';

PO.descrip = 'spm - mean image';

PO.dt = [spm_type('int16') spm_platform('bigend')];

spm_write_vol(PO,Integral);

end

spm_progress_bar('Clear');

%==========================================================================

%-function v = kspace3d(v,M)

%==========================================================================

function v = kspace3d(v,M)

% 3D rigid body transformation performed as shears in 1D Fourier space

% FORMAT v = kspace3d(v,M)

% v - image stored as a 3D array

% M - rigid body transformation matrix

%

% v - transformed image

%

% References:

% R. W. Cox and A. Jesmanowicz (1999)

% Real-Time 3D Image Registration for Functional MRI

% Magnetic Resonance in Medicine 42(6):1014-1018

%

% W. F. Eddy, M. Fitzgerald and D. C. Noll (1996)

% Improved Image Registration by Using Fourier Interpolation

% Magnetic Resonance in Medicine 36(6):923-931

[S0,S1,S2,S3] = shear_decomp(M);

d = [size(v) 1 1 1];

g = 2.^ceil(log2(d));

if any(g~=d)

tmp = v;

v = zeros(g);

v(1:d(1),1:d(2),1:d(3)) = tmp;

clear tmp;

end

% XY-shear

tmp1 = -sqrt(-1)*2*pi*([0:((g(3)-1)/2) 0 (-g(3)/2+1):-1])/g(3);

for j=1:g(2)

t = reshape( exp((j*S3(3,2) + S3(3,1)*(1:g(1)) + S3(3,4)).'*tmp1) ,[g(1) 1 g(3)]);

v(:,j,:) = real(ifft(fft(v(:,j,:),[],3).*t,[],3));

end

% XZ-shear

tmp1 = -sqrt(-1)*2*pi*([0:((g(2)-1)/2) 0 (-g(2)/2+1):-1])/g(2);

for k=1:g(3)

t = exp( (k*S2(2,3) + S2(2,1)*(1:g(1)) + S2(2,4)).'*tmp1);

v(:,:,k) = real(ifft(fft(v(:,:,k),[],2).*t,[],2));

end

% YZ-shear

tmp1 = -sqrt(-1)*2*pi*([0:((g(1)-1)/2) 0 (-g(1)/2+1):-1])/g(1);

for k=1:g(3)

t = exp( tmp1.'*(k*S1(1,3) + S1(1,2)*(1:g(2)) + S1(1,4)));

v(:,:,k) = real(ifft(fft(v(:,:,k),[],1).*t,[],1));

end

% XY-shear

tmp1 = -sqrt(-1)*2*pi*([0:((g(3)-1)/2) 0 (-g(3)/2+1):-1])/g(3);

for j=1:g(2)

t = reshape( exp( (j*S0(3,2) + S0(3,1)*(1:g(1)) + S0(3,4)).'*tmp1) ,[g(1) 1 g(3)]);

v(:,j,:) = real(ifft(fft(v(:,j,:),[],3).*t,[],3));

end

if any(g~=d), v = v(1:d(1),1:d(2),1:d(3)); end

%==========================================================================

%-function [S0,S1,S2,S3] = shear_decomp(A)

%==========================================================================

function [S0,S1,S2,S3] = shear_decomp(A)

% Decompose rotation and translation matrix A into shears S0, S1, S2 and

% S3, such that A = S0*S1*S2*S3. The original procedure is documented in:

% R. W. Cox and A. Jesmanowicz (1999)

% Real-Time 3D Image Registration for Functional MRI

% Magnetic Resonance in Medicine 42(6):1014-1018

A0 = A(1:3,1:3);

if any(abs(svd(A0)-1)>1e-7), error('Can''t decompose matrix'); end

t = A0(2,3); if t==0, t=eps; end

a0 = pinv(A0([1 2],[2 3])')*[(A0(3,2)-(A0(2,2)-1)/t) (A0(3,3)-1)]';

S0 = [1 0 0; 0 1 0; a0(1) a0(2) 1];

A1 = S0\A0; a1 = pinv(A1([2 3],[2 3])')*A1(1,[2 3])'; S1 = [1 a1(1) a1(2); 0 1 0; 0 0 1];

A2 = S1\A1; a2 = pinv(A2([1 3],[1 3])')*A2(2,[1 3])'; S2 = [1 0 0; a2(1) 1 a2(2); 0 0 1];

A3 = S2\A2; a3 = pinv(A3([1 2],[1 2])')*A3(3,[1 2])'; S3 = [1 0 0; 0 1 0; a3(1) a3(2) 1];

s3 = A(3,4)-a0(1)*A(1,4)-a0(2)*A(2,4);

s1 = A(1,4)-a1(1)*A(2,4);

s2 = A(2,4);

S0 = [[S0 [0 0 s3]'];[0 0 0 1]];

S1 = [[S1 [s1 0 0]'];[0 0 0 1]];

S2 = [[S2 [0 s2 0]'];[0 0 0 1]];

S3 = [[S3 [0 0 0]'];[0 0 0 1]];

%==========================================================================

%-function [Mask,y1,y2,y3] = getmask(M,x1,x2,x3,dim,wrp)

%==========================================================================

function [Mask,y1,y2,y3] = getmask(M,x1,x2,x3,dim,wrp)

tiny = 5e-2; % From spm_vol_utils.c

y1 = M(1,1)*x1+M(1,2)*x2+(M(1,3)*x3+M(1,4));

y2 = M(2,1)*x1+M(2,2)*x2+(M(2,3)*x3+M(2,4));

y3 = M(3,1)*x1+M(3,2)*x2+(M(3,3)*x3+M(3,4));

Mask = true(size(y1));

if ~wrp(1), Mask = Mask & (y1 >= (1-tiny) & y1 <= (dim(1)+tiny)); end

if ~wrp(2), Mask = Mask & (y2 >= (1-tiny) & y2 <= (dim(2)+tiny)); end

if ~wrp(3), Mask = Mask & (y3 >= (1-tiny) & y3 <= (dim(3)+tiny)); end

%==========================================================================

%-function vo = nan2zero(vi)

%==========================================================================

function vo = nan2zero(vi)

vo = vi;

vo(~isfinite(vo)) = 0;

function Index=find_string_in_cell_MG(str,cell_data)

% Function finds index of strin in the cell

IndexC = strfind(cell_data,str);

Index = find(not(cellfun('isempty',IndexC)));

function ME_merging_fcn(path_def,subject_data,opt,sub_id)

spm_jobman('initcfg');

pathdelim = '/';

cwd=path_def.new_folder;

sessions={

'func';

};

taskname = ['HCP'];

TEs=opt.TE;

NoOfEchoes=numel(TEs);

subjects=subject_data(:,1);

if size(subjects,2)==1

subjects(:,2)=subjects(:,1);

end

nsub=size(subjects,1);

nses=size(sessions,1);

ToDo.realign = 1;

ToDo.unwarp = 0;

ToDo.calcsumimage = 0;

ToDo.calcSNRcomp = 0;

ToDo.calctSNRcomp = 1;

outbrain_thr = 0.20;

inbrain_thr = 0.25;

tic

spm_defaults;

global defaults;

scsource = [mfilename('fullpath') '.m'];

scname= [mfilename '.m.txt'];

scdestination = fullfile(cwd,subjects{sub_id,1},scname);

copyfile(scsource,scdestination);

swd=fullfile(cwd,subjects{sub_id,1});

cd(cwd)

for ses=1:nses

disp('')

fprintf(2,['PROGRESS: sub: ',num2str(sub_id), '/', num2str(nsub),' ses: ',num2str(ses), '/', num2str(nses),'\n'])

if ToDo.realign == 1

%%%%%%%%%%%%%%%%%%%%%%%%%%%%%%%%%%%%%%%%%%%%%%%%%%%%%%%%%%%%

% Set up Realign

%%%%%%%%%%%%%%%%%%%%%%%%%%%%%%%%%%%%%%%%%%%%%%%%%%%%%%%%%%%%

P = [];

selected_scans = [];

direc{ses} = [cwd pathdelim subjects{sub_id,1} pathdelim sessions{ses,1}];

q=fullfile(cwd, subject_data{sub_id,1},subject_data{sub_id,2},subject_data{sub_id,3} );

suffix=opt.BOLD_suffix;

k=strfind(q,suffix);

q(k-1)=num2str(2);

P = strvcat(P,q);

for i=1:size(P,1)

selected_scans{i,1} = [P(i,:)];

end

if ToDo.unwarp == 1

ReaUnwPrep = 'u';

for ee=1:NoOfEchoes

RUW.data(ee).scans=selected_scans(ee);

RUW.data(ee).pmscan='';

end

RUW.eoptions = struct( 'quality', 0.9,...

'sep', 4,...

'fwhm', 5,...

'rtm', 0,...

'einterp', 2,...

'ewrap', [0 0 0],...

'weight', '');

RUW.uweoptions = struct( 'basfcn', [12 12],...

'regorder', 1,...

'lambda', 100000,...

'jm', 0,...

'fot', [4 5],...

'sot', [],...

'uwfwhm', 4,...

'rem', 1,...

'noi', 5,...

'expround', 'Average');

RUW.uwroptions = struct( 'uwwhich', [2 1],...

'rinterp', 4,...

'wrap', [0 0 0],...

'mask', 1,...

'prefix', 'u');

matlabbatch{1}.spm.spatial.realignunwarp = RUW;

spm_jobman('run',matlabbatch);

clear matlabbatch RUW P q selected_scans dfiles

else

ReaUnwPrep = 'r';

for ee=1:1

dfiles(ee)={selected_scans(ee)};

end

REW.data = dfiles;

REW.eoptions = struct( 'quality', 0.9,...

'sep', 4,...

'fwhm', 5,...

'rtm', 0,...

'interp', 2,...

'wrap', [0 0 0],...

'weight', '');

REW.roptions = struct( 'which', [2 1],...

'interp', 4,...

'wrap', [0 0 0],...

'mask', 1,...

'prefix', 'r');

matlabbatch{1}.spm.spatial.realign.estwrite = REW;

spm_jobman('run',matlabbatch);

clear matlabbatch REW P q selected_scans dfiles

direc{ses} = [cwd pathdelim subjects{sub_id,1} pathdelim sessions{ses,1}];

q=fullfile(cwd, subject_data{sub_id,1},subject_data{sub_id,2},subject_data{sub_id,3} );

suffix=opt.BOLD_suffix;

k=strfind(q,suffix);

q(k-1)=num2str(2);

for e = [1 3]

otherdirec{ses} = [cwd pathdelim subjects{sub_id,1} pathdelim sessions{ses,1}];

otherq = spm_select('FPList',otherdirec{ses},['^' subjects{sub_id,2} '_task-' taskname '.*_echo-' num2str(e) '_bold.nii$']);

otherq=fullfile(cwd, subject_data{sub_id,1},subject_data{sub_id,2},subject_data{sub_id,3} );

suffix=opt.BOLD_suffix;

k=strfind(otherq,suffix);

otherq(k-1)=num2str(e);

rewrite_mat_reslice(q,otherq)

clear otherdirec otherq

end

end

end

direc{ses} = [cwd pathdelim subjects{sub_id,1} pathdelim sessions{ses,1}];

tmp_data_name=subject_data{sub_id,3};

suffix=opt.BOLD_suffix;

k=strfind(tmp_data_name,suffix);

tmp_data_name(k-1:end)=[];

q = spm_select('FPList',direc{ses},['^r',tmp_data_name,'.*\.nii$']);

fv = spm_vol(q);

nfiles = size(fv,1);

nscans = nfiles/NoOfEchoes;

dim = fv(1).dim;

meY=cell(3);

for echo=1:NoOfEchoes

fileindices = ((echo-1)*nscans)+1:(echo*nscans);

[meY{echo}, ~] = spm_read_vols(fv(fileindices));

end

obv_ii=zeros(size(meY{1}(:,:,:,1)));

ibv_ii1e=zeros(size(meY{1}(:,:,:,1)));

ibv_ii=zeros(size(meY{1}(:,:,:,1)));

for scan=1:min(nscans,100)

for echo=1:NoOfEchoes

Yi = meY{echo}(:,:,:,scan);

meanY = mean(Yi(~isnan(Yi)));

othr=meanY*outbrain_thr;

ithr=meanY*inbrain_thr;

obv_ii = obv_ii + ((Yi<othr)&(~isnan(Yi)));

ibv_ii = ibv_ii + ((Yi>ithr)&(~isnan(Yi)));

if echo==1

ibv_ii1e = ibv_ii1e + ((Yi>ithr)&(~isnan(Yi)));

end

end

end

obv_indices = (obv_ii==round(min(nscans,100)*NoOfEchoes));

ibvAe_indices = (ibv_ii==round(min(nscans,100)*NoOfEchoes));

ibv1e_indices = (ibv_ii1e==round(min(nscans,100)));

ibv_indices = ibv1e_indices;

nvox = dim(1)*dim(2)*dim(3);

re_ibv_indices = reshape(ibv_indices,nvox,1);

re_obv_indices = reshape(obv_indices,nvox,1);

for echo=1:NoOfEchoes

re_meY{echo} = reshape(meY{echo},[],nscans);

end

for scan=1:nscans

for echo=1:NoOfEchoes

Yi = meY{echo}(:,:,:,scan);

bckg_sig(scan,echo) = mean(Yi(obv_indices));

end

end

for echo=1:NoOfEchoes

ref_sig(echo) = mean(bckg_sig(:,echo));

end

if ToDo.calcsumimage == 1

sumY=zeros(dim(1),dim(2),dim(3),nscans);

re_sumY=reshape(sumY,[],nscans);

end

if ToDo.calctSNRcomp == 1

tsnrY=zeros(dim(1),dim(2),dim(3),nscans);

re_tsnrY=reshape(tsnrY,[],nscans);

end

if ToDo.calcSNRcomp == 1

snrY=zeros(dim(1),dim(2),dim(3),nscans);

re_snrY=reshape(snrY,[],nscans);

end

nvox = dim(1)*dim(2)*dim(3);

nsegments = dim(3);

segsize = ceil(nvox/nsegments);

for seg=1:nsegments

segindices{seg} = ((seg-1)*segsize)+1:min((seg*segsize),nvox);

end

if ToDo.calctSNRcomp == 1

sumtSNR=zeros(nvox,1);

for echo=1:NoOfEchoes

for seg=1:nsegments

re_tSNR{echo}(segindices{seg},1) = mean(re_meY{echo}(segindices{seg},:),2,'omitnan')./std(re_meY{echo}(segindices{seg},:),0,2,'omitnan');

sumtSNR(segindices{seg},1) = sumtSNR(segindices{seg},1) + (re_tSNR{echo}(segindices{seg},1)*TEs(echo));

end

end

end

if ToDo.calcsumimage == 1

sumY=zeros(size(re_meY{1}));

for echo=1:NoOfEchoes

for seg=1:nsegments

sumY(segindices{seg})=sumY(segindices{seg})+re_meY{echo}(segindices{seg});

end

end

end

if ToDo.calctSNRcomp == 1

re_tsnrY=zeros(size(re_meY{1}));

for seg=1:nsegments

for echo=1:NoOfEchoes

re_tsnrY(segindices{seg},:) = re_tsnrY(segindices{seg},:)+(re_meY{echo}(segindices{seg},:).*repmat(re_tSNR{echo}(segindices{seg},1)*TEs(echo),[1 nscans]));

end

re_tsnrY(segindices{seg},:) = re_tsnrY(segindices{seg},:)./repmat(sumtSNR(segindices{seg},1),[1 nscans]);

end

end

[pathstr,name,ext]=fileparts(fv(1).fname);

varfname=fullfile(pathstr,'ProcessMultiEcho_vars.mat');

save(varfname,'NoOfEchoes','nscans','nfiles','fv','q','dim');

if ToDo.calctSNRcomp == 1

for echo=1:NoOfEchoes

tSNR{echo} = reshape(re_tSNR{echo},dim(1),dim(2),dim(3));

end

save(varfname,'tSNR','sumtSNR','-append');

end

if ToDo.calcSNRcomp == 1

for echo=1:NoOfEchoes

SNR{echo} = reshape(re_SNR{echo},dim(1),dim(2),dim(3));

end

save(varfname,'SNR','ref_sig','-append');

end

if ToDo.calctSNRcomp == 1

for echo=1:NoOfEchoes

Vo=fv(1);

fname=Vo.fname;

[pathstr,name,ext]=fileparts(fname);

Vo.fname=fullfile(pathstr,['tSNR_' num2str(echo) '_' name ext]);

Vo.descrip=['tSNR_' num2str(echo)];

Vo=spm_write_vol(Vo,tSNR{echo});

end

end

if ToDo.calcSNRcomp == 1

for echo=1:NoOfEchoes

Vo=fv(1);

fname=Vo.fname;

[pathstr,name,ext]=fileparts(fname);

Vo.fname=fullfile(pathstr,['SNR_' num2str(echo) '_' name ext]);

Vo.descrip=['SNR_' num2str(echo)];

Vo=spm_write_vol(Vo,SNR{echo});

end

end

if ToDo.calcsumimage == 1

sumY = reshape(re_sumY,dim(1),dim(2),dim(3),nscans);

data=sumY;

for ii=1:nscans

V(ii)=fv(ii);

fname=V(ii).fname;

[pathstr,name,ext]=fileparts(fname);

V(ii).fname=fullfile(pathstr,['sue_' name ext]);

V(ii).dim=size(data(:,:,:,ii));

V(ii).descrip='composite multiecho - sum';

spm_write_vol(V(ii),data(:,:,:,ii));

end

end

if ToDo.calcSNRcomp == 1

snrY = reshape(re_snrY,dim(1),dim(2),dim(3),nscans);

data=snrY;

for ii=1:nscans

V(ii)=fv(ii);

fname=V(ii).fname;

[pathstr,name,ext]=fileparts(fname);

V(ii).fname=fullfile(pathstr,['sne_' name ext]);

V(ii).dim=size(data(:,:,:,ii));

V(ii).descrip='composite multiecho - SNR weighted';

spm_write_vol(V(ii),data(:,:,:,ii));

end

end

if ToDo.calctSNRcomp == 1

tsnrY = reshape(re_tsnrY,dim(1),dim(2),dim(3),nscans);

data=tsnrY;

for ii=1:nscans

V(ii)=fv(ii);

fname=V(ii).fname;

[pathstr,name,ext]=fileparts(fname);

V(ii).fname=fullfile(pathstr,['tse_' name ext]);

V(ii).dim=size(data(:,:,:,ii));

V(ii).descrip='composite multiecho - tSNR weighted';

spm_write_vol(V(ii),data(:,:,:,ii));

end

end

clear meY snrY tsnrY sumY tSNR SNR re_meY re_tsnrY;

end

function rewrite_mat_reslice(files,otherfiles)

mujfile_mask = spm_vol(files);

mujfile_other = spm_vol(otherfiles);

for scan = 1:size(mujfile_other,1)

mujfile_other(scan).mat= mujfile_mask(scan).mat;

spm_get_space([mujfile_other(scan).fname ',' num2str(mujfile_other(scan).n)], mujfile_mask(scan).mat);

end

matlabbatch{1}.spm.spatial.realign.write.data = {otherfiles};

matlabbatch{1}.spm.spatial.realign.write.roptions.which = [2 0];

matlabbatch{1}.spm.spatial.realign.write.roptions.interp = 4;

matlabbatch{1}.spm.spatial.realign.write.roptions.wrap = [0 0 0];

matlabbatch{1}.spm.spatial.realign.write.roptions.mask = 1;

matlabbatch{1}.spm.spatial.realign.write.roptions.prefix = 'r';

spm_jobman('run',matlabbatch);

clear matlabbatch;

Reference:

1. Tzourio-Mazoyer, N. *et al.* Automated Anatomical Labeling of Activations in SPM Using a Macroscopic Anatomical Parcellation of the MNI MRI Single-Subject Brain. *NeuroImage* **15**, 273–289 (2002).
